# Synthesis, Biological, Spectroscopic and Computational Investigations of Novel *N*-Acylhydrazone Derivatives of Pyrrolo[3,4-*d*]pyridazinone as Dual COX/LOX Inhibitors

**DOI:** 10.3390/molecules28145479

**Published:** 2023-07-18

**Authors:** Jakub Mikus, Piotr Świątek, Patrycja Przybyła, Edward Krzyżak, Aleksandra Marciniak, Aleksadra Kotynia, Aleksandra Redzicka, Benita Wiatrak, Paulina Jawień, Tomasz Gębarowski, Łukasz Szczukowski

**Affiliations:** 1 Student Science Club of Medicinal Chemistry, Department of Medicinal Chemistry, Faculty of Pharmacy, Wroclaw Medical University, Borowska 211, 50-556 Wrocław, Poland; jakub.mikus@student.umw.edu.pl (J.M.); patrycja.przybyla@student.umw.edu.pl (P.P.); 2 Department of Medicinal Chemistry, Faculty of Pharmacy, Wroclaw Medical University, Borowska 211, 50-556 Wrocław, Poland; aleksandra.redzicka@umw.edu.pl; 3 Department of Basic Chemical Sciences, Faculty of Pharmacy, Wroclaw Medical University, Borowska 211a, 50-556 Wrocław, Poland; edward.krzyzak@umw.edu.pl (E.K.); aleksandra.marciniak@umw.edu.pl (A.M.); aleksandra.kotynia@umw.edu.pl (A.K.); 4 Department of Pharmacology, Faculty of Medicine, Wroclaw Medical University, Mikulicza-Radeckiego 2, 50-345 Wrocław, Poland; benita.wiatrak@umw.edu.pl; 5 Department of Biostructure and Animal Physiology, Division of Animal Anatomy, Faculty of Veterinary Medicine, Wroclaw University of Environmental and Life Sciences, Kożuchowska 1, 51-631 Wrocław, Poland; paulina.jawien@upwr.edu.pl (P.J.); tomasz.gebarowski@upwr.edu.pl (T.G.)

**Keywords:** AAG, ADME, cyclooxygenase, fluorescence spectroscopy, HSA, inflammation, lipoxygenase, molecular docking, NAH, pyridazinone

## Abstract

Secure and efficient treatment of diverse pain and inflammatory disorders is continually challenging. Although NSAIDs and other painkillers are well-known and commonly available, they are sometimes insufficient and can cause dangerous adverse effects. As yet reported, derivatives of pyrrolo[3,4-*d*]pyridazinone are potent COX-2 inhibitors with a COX-2/COX-1 selectivity index better than meloxicam. Considering that *N*-acylhydrazone (NAH) moiety is a privileged structure occurring in many promising drug candidates, we decided to introduce this pharmacophore into new series of pyrrolo[3,4-*d*]pyridazinone derivatives. The current paper presents the synthesis and in vitro, spectroscopic, and in silico studies evaluating the biological and physicochemical properties of NAH derivatives of pyrrolo[3,4-*d*]pyridazinone. Novel compounds **5a**-**c**–**7a**-**c** were received with high purity and good yields and did not show cytotoxicity in the MTT assay. Their COX-1, COX-2, and 15-LOX inhibitory activities were estimated using enzymatic tests and molecular docking studies. The title *N*-acylhydrazones appeared to be promising dual COX/LOX inhibitors. Moreover, spectroscopic and computational methods revealed that new compounds form stable complexes with the most abundant plasma proteins–AAG and HSA, but do not destabilize their secondary structure. Additionally, predicted pharmacokinetic and drug-likeness properties of investigated molecules suggest their potentially good membrane permeability and satisfactory bioavailability.

## 1. Introduction

The organism’s exposure to different harmful factors such as toxins, allergens, radiation, pathogens, or injury, activates the body’s immune system. Leukocytes, mainly neutrophils, are recruited to the damaged, malfunctioning, or infected tissue. Then, the release of cytokines, chemokines, and different transmitters and paracrine hormones such as tumor necrosis factor α (TNF-α) promotes tissue infiltration by other immune system cells such as macrophages, monocytes, or natural killer (NK) cells. These lead to the development of inflammation and other processes engaged in maintaining homeostasis, which is necessary to minimize tissue damage [1,2,3,4].

When considering plenty of diverse mediators engaged in initiating and promoting the inflammatory response, bioactive lipids such as prostaglandins (PGs) and leukotrienes (LTs) are probably among the best-known and well-described in the literature. These mediators are derived from arachidonic acid (AA) and are synthesized in a multi-step reaction catalyzed by cyclooxygenases (COXs) and lipoxygenases (LOXs), respectively [1,2,3,4,5,6,7].

Cyclooxygenase occurs in two isoforms—constitutive named COX-1 and inducible COX-2. These enzymes are responsible for the production of PGs, which take part in various physiological and pathophysiological processes such as inflammation, blood coagulation, protection of the gastric mucosa, and vasodilation or vasoconstriction [1,2,3,4,5,6,7,8]. On the other hand, LOXs are expressed in immune, epithelial, and tumor cells and catalyze the process of hydroperoxidation of polyunsaturated fatty acids (PUFAs), such as linoleic or arachidonic acid leading to the formation of LTs and lipoxins. Leukotrienes promote leukocyte chemotaxis and recruit different types of T cells. Therefore they have a significant role in a large number of inflammatory diseases with allergic etiology, such as neutrophilic or aspirin-induced asthma (AIA), allergic rhinitis, atopic dermatitis, osteoarthritis, and conjunctivitis [5,9,10,11].

Nonsteroidal and anti-inflammatory drugs (NSAIDs) inhibit the activity of COX and are commonly used to cure pain and inflammation. Unfortunately, long-term usage of these medicaments increases the risk of occurrence of side effects related especially to the gastrointestinal (GI) and cardiovascular (CV) systems [7,12,13,14,15,16,17,18]. Furthermore, chronic COX inhibition can elevate the LOX-dependent metabolism of AA. As a result, a higher level of LTs can cause airway inflammation and bronchoconstriction, which may lead to the development of the abovementioned AIA [9,10,11].

For these reasons, the simultaneous use of effective COX and LOX inhibitors seems to be a rational solution that can significantly increase the effectiveness and safety of the therapy [19,20,21]. Although many potent LOX inhibitors have been reported in the literature already, zileuton remains the only one approved for the treatment of different inflammatory disorders. What is worse, this drug presents poor pharmacokinetic properties and can cause liver dysfunction [9,10,22].

Therefore the discovery of new, effective, and safe anti-inflammatory agents could be groundbreaking for contemporary pharmacy and medicine. A particularly interesting trend in medicinal chemistry concerns the development of compounds with dual COX/LOX inhibitory activity, which can serve as potent anti-inflammatory drug candidates with a much-improved safety profile [19,20,21]. A widespread strategy used in the design and synthesis of new bioactive molecules relies on the introduction of different privilege structures or pharmacophores to the investigated molecules. By this, we mean a common, linear, or cyclic subunit, which can be recognized and bound by distinct enzymes and receptors and, for this reason, is present in numerous compounds [23].

The *N*-acylhydrazone (NAH) moiety is one of the most extensively studied privileged structures in medicinal chemistry nowadays. This substructure is present in a great number of potent compounds with various pharmacological activities such as anticancer, antimicrobial, antiviral, anti-platelet, antinociceptive, and anti-inflammatory [23,24,25,26,27,28,29,30,31,32,33,34,35]. It should be noted that *N*-acylhydrazone derivatives can affect different molecular targets and signaling pathways associated with inflammatory disorders. The literature reports diverse drug candidates possessing NAH residue, which act as COX and/or LOX inhibitors, transient receptor potential cation channel subfamily V member 1 (TRPV1) antagonists, and others that modulate the release and activity of cytokines and reveal antioxidant activity. The complex and wide-range anti-inflammatory effect of *N*-acylhydrazones can be explained by the relative acidity of the amide hydrogen and its ability to stabilize free radicals. Therefore, the NAH group can imitate the bis-allylic moiety of PUFAs such as AA [23,24,29,30,31,32,33,34,35].

**Figure 1:**
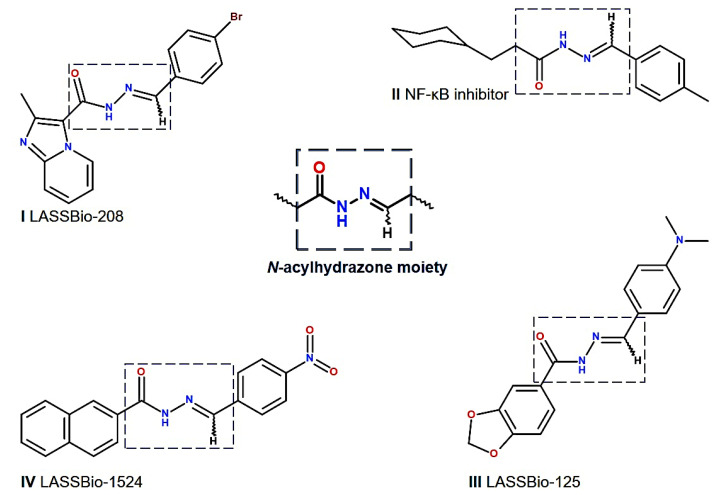
Examples of compounds build on the *N*-acylhydrazone framework with significant anti-inflammatory activity [23,30,34].

Figure 1 presents examples of *N*-**acylhydrazone** derivatives with promising analgesic and anti-inflammatory profiles. Compounds **I** (LASSBio-208) [30] and **III** (LASSBio-125) [30] showed significant analgesic activity in experiments performed in vivo. These compounds were more potent than dipyrone in acetic acid-induced mice models of abdominal constriction and indomethacin in the formalin test. What is worth noticing, these derivatives were active after oral administration [30]. On the other hand, compounds **II** [23] and **IV** (LASSBio-1524) [34] are characterized by potent anti-inflammatory properties because they inhibit the activity of nuclear factor kappa-light-chain-enhancer of activated B cells (NF-κB), where overexpression is associated with such inflammatory disorders as colitis or arthritis [23,34]. Moreover, by structural modifications of **IV**, a series of promising anti-TNF-α agents have been received [34]. These are just some of the many examples that prove the great importance of NAH derivatives in the design of new anti-inflammatory and analgesic drugs [23,29,30,31,32,33,34,35].

**Figure 2:**
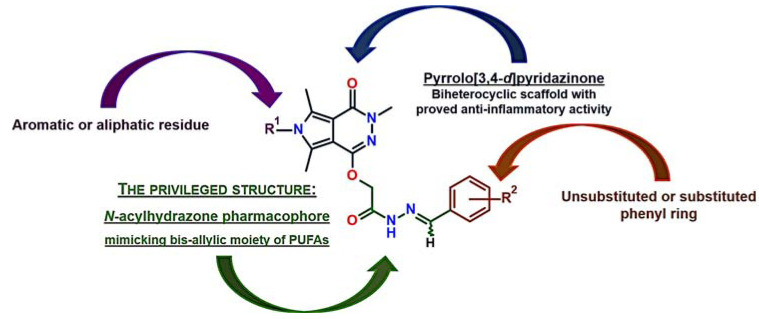
The concept of new *N*-acylhydrazone derivatives of pyrrolo[3,4-*d*]pyridazinone.

Our previous papers reported pyrrolo[3,4-*d*]pyridazinone derivatives with promising analgesic and anti-inflammatory activity in both in vitro and in vivo experiments. Compounds with 1,3,4-oxadiazole or 1,2,4-triazole moiety acted as potent COX inhibitors that spare gastric mucosa. Moreover, studied derivatives showed anti-oxidant activity and reduced the level of inflammatory mediators such as myeloperoxidase (MPO) and TNF-α [36,37,38,39,40,41]. Considering the above, we synthesized a series of the *N*-acylhydrazone derivatives of pyrrolo[3,4-*d*]pyridazinone. The general structure of title compounds is presented in Figure 2.

In the current paper, we report the synthesis, preliminary biological, and molecular docking studies describing novel compounds’ dual anti-COX/LOX activity and evaluation of their binding mode with the most abundant plasma proteins. Moreover, some computational studies have been carried out to estimate some of their pharmacokinetic properties.

## 2. Results

### 2.1. Chemistry

**Scheme 1:**
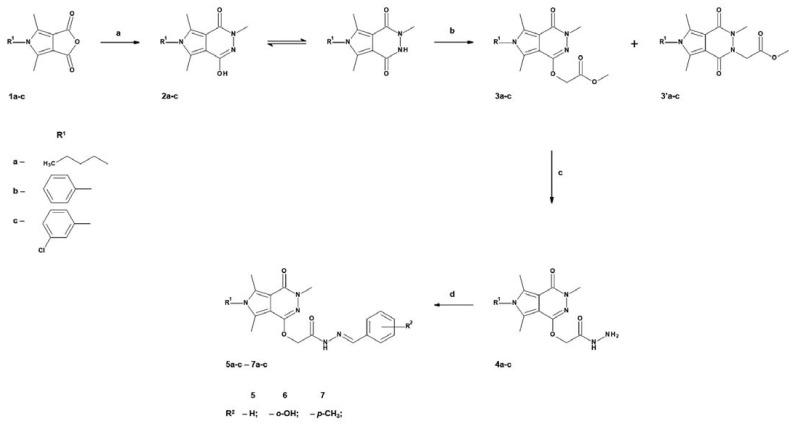
The synthesis of the intermediates **2a-c–4a-c** and the title compounds **5a-c–7a-c**. Reagents and reaction conditions: (**a**) methylhydrazine, MeCN, reflux ~5 h; (**b**) methyl chloroacetate, K_2_CO_3_, MeCN, reflux ~5 h; (**c**) hydrazine hydrate 98%, methanol, reflux ~3 h; (**d**) an appropriate aromatic aldehyde, a catalytic amount of acetic acid, ethanol, reflux ~3 h.

The objective of this study was the design and synthesis of new series of *N*-acylhydrazone derivatives of pyrrolo[3,4-*d*]pyridazinone. The synthesis of intermediates **2a-c–4a-c** and title compounds **5a-c–7a-c** is presented in Scheme 1. This section describes the synthetic pathway and spectroscopic properties of novel compounds **2c–4c** and nine final *N*-acylhydrazones **5a-c–7a-c**. The synthetic protocols and all spectroscopic and analytical data concerning compounds **1a**-**c**, **2a**-**b**, **3a**-**b**, and **4a**-**b** were reported in our previous manuscripts [36,42,43,44].

The first step of the presented synthesis relied on the formation of a new 6-(3-chlorophenyl)-3,5,7-trimethyl-2*H*-pyrrolo[3,4-*d*]pyridazine-1,4-dione **2c**. Therefore, the anhydride **1c** was refluxed in acetonitrile with an excessive amount of methylhydrazine. A formed white precipitate of the desired compound **2c** was left overnight, filtered off, washed with cold ethanol, and purified by the crystallization from this solvent. In the next step, the alkylation of 2c with methyl chloroacetate allowed the reception of ester derivatives 3c and 3′c. The 1H NMR spectra of these compounds displayed a characteristic 3-proton singlet at δ~3.69 ppm, corresponding with the methyl group of ester residue and a 2-proton singlet at δ~4.87 ppm belonging to methylene linker. Moreover, a characteristic band in the Fourier-transform infrared (FT-IR) spectrum of about 1762 cm^−1^ confirms the presence of the additional carbonyl group.

**Figure 3:**
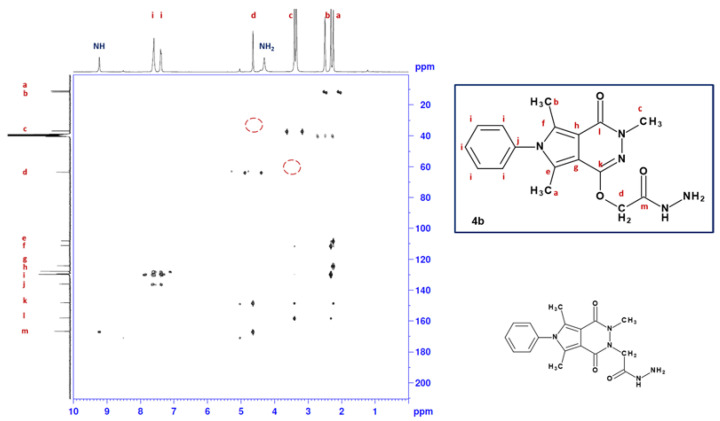
The heteronuclear multiple bond correlation (HMBC) spectrum of compound **4b** [36]. A red dashed line marks correlations between the methylene group and 3-CH_3_ substituent, which would have appeared in the case of *N*-isomer (structure below **4b**).

Because of the phenomenon of keto-enol tautomerism occurring in the structure 2c, a mixture of *N*- and *O*- isomers was received, which is in good agreement with our previous findings [36,44]. The isomers were separated using column chromatography (CC), giving products with high purity. According to our former experiences, we have distinguished and identified both ester derivatives based on ^1^H NMR spectra and heteronuclear multiple bond correlation (HMBC) experiments. In the case of *O*-isomer **3c**, protons belonging to the methyl groups in positions 5 and 7 of the pyrrolo[3,4-*d*]pyridazinone are not in the same electronic environment and, as a consequence, they are not chemically equivalent. These protons are not homotopic and have different resonance frequencies. Therefore, their signals in the ^1^H NMR spectrum appear as two separate 3-proton singlets (δ~2.27 ppm s, 3H, 7-CH_3_; δ~2.34 ppm s, 3H, 5-CH_3_). Moreover, the HMBC experiment provides the correlation of protons and carbons separated by at most four bounds. For this reason, in the case of every *O*-isomer of pyrrolo[3,4-*d*]pyridazinone, we can observe that the hydrogen and carbon atoms in the methylene group (O-CH_2_-C=O) do not correlate with those in the methyl substituent in the third position of the pyrrolo[3,4-*d*]pyridazinone scaffold. This is because these atoms are separated by six bonds. Figure 3 presents the exemplary HMBC spectrum of previously reported compound **4b** [36], which supports this theory. Red dashed lines mark correlations (between signals **c** and **d**) which would have appeared in the case of *N*-isomer.

As already mentioned, *O*-alkylation is privileged under the reaction conditions used. Therefore we managed to obtain mainly *O*-ester **3c** with a very good yield (>70%). On the other hand, *N*-isomer **3′c** was formed with poor efficiency (<10%). For this reason, all other compounds presented in this study are *O*-substituted derivatives of pyrrolo[3,4-*d*]pyridazinone.

In the next step, ester **3c** was refluxed with an excess of hydrazine hydrate (98%) in absolute ethanol, which led to the formation of appropriate hydrazide derivative **4c**. The characteristic absorption bands in the FT-IR spectrum of about 3248 and 3331 cm^−1^ indicate the presence of -NH- and -NH_2_ groups. Moreover, by analyzing the ^1^H NMR spectrum of **4c**, the lack of the 3-proton singlet of the ester methyl group and the presence of distinctive peaks near δ~4.34 and δ~9.22 belonging to the protons (-NH- and -NH_2_) of hydrazide residue can be noticed.

Finally, the already reported hydrazides **4a**-**b** and new hydrazide derivative **4c** were dissolved in hot ethanol with a catalytic amount of acetic acid. Subsequently, an equimolar quantity of different aromatic aldehydes was added. The progress of the reaction was controlled by the use of thin-layer chromatography (TLC). The mixture was refluxed till the substrates disappeared on TLC. After cooling, the formed precipitate was filtered off and crystallized from the appropriate solvent. This convenient and efficient reaction allowed us to receive a series of new *N*-acylhydrazone derivatives of pyrrolo[3,4-*d*]pyridazinone.

All crude intermediates and final products were purified by column chromatography or crystallization from the appropriate solvent. The structures of novel derivatives were established and confirmed by spectroscopic techniques: ^1^H NMR, ^13^C NMR, MS, FT-IR, and on the basis of their physicochemical properties. Supplementary Materials provides the spectra and formulas of all new compounds.

When considering the ^1^H NMR spectra of final compounds **5a-c–7a-c,** we can observe new, characteristic signals assigned to the azomethine moiety and additional peaks in the aromatic region. Moreover, a double-proton signal of the -NH_2_ group has not been registered. The presence of the azomethine group is also confirmed by stretching absorption bands of C–H in the range of 2970–2850 cm^−1^ and a C=N band of about 1550 cm^−1^ in the FT-IR spectra. Additionally, a strong band observed within 1710–1680 cm^−1^ is related to the carbonyl group of hydrazone moiety.

**Figure 4:**
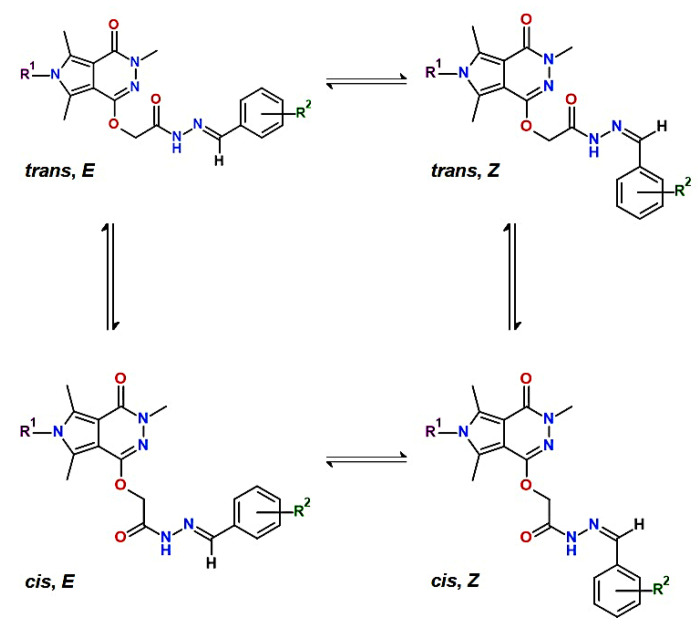
The structures of all possible isomers of *N*-acylhydrazone derivatives **5a-c–7a-c**.

It is worth noticing that in the case of ^1^H NMR spectra of title *N*-acylhydrazones **5a-c–7a-c**, the signals of protons belonging to the amidic nitrogen, azomethine group, methylene linker, and also Ar-OH group (in case of **6a**-**c**) are doubled. It can be explained by the fact that these compounds could exist as *Z*/*E* geometrical isomers about the C=N bond and cis/trans conformers due to hindered rotation on the C–N amide bond [45,46,47,48]. The structures of all possible configurational and conformational isomers of title hydrazones are presented in Figure 4.

Isomerization can be induced by light or by temperature. In the case of arylhydrazones, the *Z* isomers can be stabilized by an intramolecular hydrogen bond formed with an amidic nitrogen atom. The ^1^H NMR spectra were performed in dimethyl-*d*_6_ sulfoxide (DMSO-*d*_6_), a polar solvent that forms competitive hydrogen bonds. Therefore our compounds exist in DMSO-*d*_6_ solution only in the form of an *E* isomer, and no signals of *Z* configurational isomer were recorded. Nevertheless, the simultaneous presence of cis/trans conformers was confirmed by double signals [45,46,47,48]. Two peaks for methylene linker, azomethine group, and protons of hydroxyl substituent (in case of **6a**-**c**) and amide nitrogen were registered in the range δ~4.74–5.28; 7.99–8.44; 10.03–11.02 and 11.48–11.82 ppm, respectively. According to the literature, the signals with greater chemical shifts can be assigned to cis amide conformers [45].

### 2.2. Evaluation of Viability

The viability of the normal human dermal fibroblasts (NHDF) cell culture after 24 h incubation with the tested compounds **5a-c–7a-c** was assessed by carrying out the MTT assay. This test was performed according to ISO 10993 part 5, Appendix C. For all tested compounds, the IC_50_ values were calculated using four-parameter logistics. The results are shown in Table 1.

All examined derivatives did not show cytotoxic potential (cells viability over 70%); therefore, they were qualified for further in vitro experiments.

### 2.3. Cyclooxygenase (COX-1 and COX-2) and 15-Lipoxygenase (15-LOX) Inhibition Studies

#### 2.3.1. In Vitro Inhibition Assay

The COX-1 and COX-2 inhibitory activity of tested compounds **5a**-**c–7a**-**c** was assessed after 2 min incubation at 100 µM concentration using the Cayman’s COX Colorimetric Inhibitor Screening Assay Kit (Cat # 701050). Three worldwide known NSAIDs—meloxicam, celecoxib, and diclofenac—have been used as references. The impact of the title *N*-acylhydrazone derivatives and zileuton (standard LOX inhibitor) on the activity of 15-LOX was evaluated with an incubation time of 5 min according to the procedure given by the kit manufacturer—Cayman’s Lipoxygenase Inhibitor Screening Assay Kit (760700). Afterward, the IC_50_ values, i.e., concentrations at which 50% inhibition of enzyme activity occurred, and the COX-2/COX-1 selectivity ratios were calculated for each compound and reference drug. The results are shown in Table 2.

The studies have shown that the tested derivatives have an affinity to both isoforms of cyclooxygenase; however, they inhibit the activity of COX-2 much stronger than COX-1. Only compound **7c** does not affect the COX-1 isoform and acts as a selective COX-2 inhibitor. On the other hand, **6c** turned out to be the most potent COX-2 inhibitor, in case of which 50% inhibition of the activity of the enzyme occurred at the lowest concentration. It is worth noticing that all tested compounds are statistically significantly more effective COX-2 inhibitors than meloxicam. The COX-2/COX-1 selectivity index of all tested derivatives is also superior to this reference drug. In relation to the COX-1 isoform, the activity of tested compounds is rather similar to meloxicam. The differences are statistically significant only in the case of **5a**, **6a**, **5b**, and **6c**. The COX-2 inhibitory activity of title *N*-acylhydrazones is much lower compared to celecoxib. On the other hand, with reference to diclofenac, the examined derivatives revealed comparable affinity to COX-2 and significantly lower to COX-1 at the same time. Only **6c** was statistically significantly more potent in inhibiting COX-2 than diclofenac.

All tested compounds inhibited 15-LOX in the concentration range of 12.7–15.3 µM. Derivatives **5a** and **5c** showed statistically significantly greater 15-LOX inhibitory activity than zileuton. On the other hand, molecules **6a-c** and **7b** inhibited LOX-15 statistically significantly worse than zileuton.

#### 2.3.2. Molecular Docking Studies

To investigate how *N*-acylhydrazone derivatives of pyrrolo[3,4-*d*]pyridazinone interact with COX-1, COX-2, and 15-LOX molecular docking studies have been carried out as well. The crystal structure of COX-1 with co-crystalized meloxicam (PBD:4O1Z [49]), COX-2 with meloxicam (PBD: 4M11 [49]), and 15-LOX with ligand C8B (PBD: 4NRE [50]) were used for calculations. The docking results showed that for all studied compounds, the binding affinity is negative (Table 3). This indicates that stable complexes are formed. The docking scores for the interactions within the active site ranged from −9.4 to −5.9 kcal/mol for COX-1, −10.9 to −8.0 kcal/mol for COX-2, and −10.3 to −8.0 kcal/mol for 15-LOX.

When considering the interactions of title NAH derivatives with both isoforms of cyclooxygenase, the best results are gained for compounds of series **6**, with 2-OH substituent in the phenyl ring of *N*-acylhydrazone moiety, and the worst for compounds with 4-CH_3_ substituent in the phenyl ring (series **7**), i.e., series **6** > series **5** > series **7**. Moreover, compounds of the **c** series, with 3-chlorophenyl residue in position 6 of the pyrrolo[3,4-*d*]pyridazinone core, interact more strongly than compounds with phenyl and n-butyl substituent, i.e., series **c** > series **b** > series **a**. For interactions with lipoxygenase, the binding affinity is more negative for series **6** and **7** than for series **5**, in series **a**, and also for series **5**, **7** than **6**, in series **b** and series **c** (Table 3).

**Figure 5:**
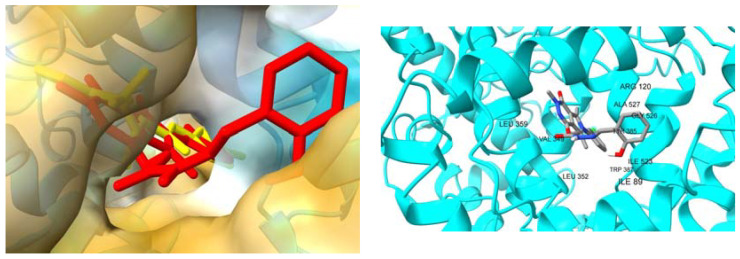
The location of **6c** (red) and meloxicam (yellow) in the COX-1 pocket (**left**). Docking pose of **6c** (**right**).

Figure 5 shows the location of compound **6c** (with the most negative binding affinity; red) and meloxicam (yellow) in the COX-1 active site. Acid residues involved in the stabilization of the COX-1/**6c** complex are also presented. The 6-(3-chlorophenyl)-3,5,7-trimethylpyrrolo[3,4-*d*]pyridazinone scaffold interacts with Ala527, Leu352 via π-sigma contacts, with Tyr385, Trp387, Val349, Ala527, Leu352, Ile527, Leu359 via π-alkyl or contacts alkyl and with Gly526 via π-π-stacking interactions. The phenyl group of *N*-acylhydrazone moiety interacts by π-sigma contacts with Ile89 and by π-cation with Arg120. No hydrogen bonds were found.

**Figure 6:**
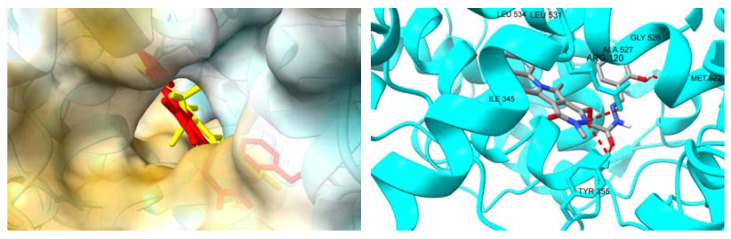
The location of **6b** (red) and meloxicam (yellow) in the COX-2 pocket (**left**). Docking pose of **6b** (**right**). Hydrogen bonds are marked in red.

As shown in Table 3, the binding affinities for interactions with COX-2, in which the active pocket is slightly larger than in the case of COX-1, are lower than those calculated for COX-1. This means that the forming complexes are better stabilized. Figure 6 presents the location of compound **6b** (with the most lower binding affinity; red) and meloxicam (yellow) in the COX-2 cavity. Acid residues involved in the stabilization of the COX-2/**6b** complex are also shown. It is stabilized by the formation of four hydrogen bonds between Tyr355 and the carbonyl of hydrazone moiety (2.59 Å) and carbonyl of pyrrolo[3,4-*d*]pyridazinone core (2.99 Å), Arg120 and nitrogen atom of pyrrolo[3,4-*d*]pyridazinone (3.17 Å), and Met522 and hydroxyl substituent in phenyl ring (2.06 Å). The hydrogen bonds with Arg120 and Tyr355 are observed in the binding models of well-known COX-2 inhibitors, such as naproxen or meloxicam [49]. Complex COX-2/**6b** is also stabilized by hydrophobic interactions (π-alkyl) with Leu531, Leu534, Ile345, Arg120, and Ala527.

**Figure 7:**
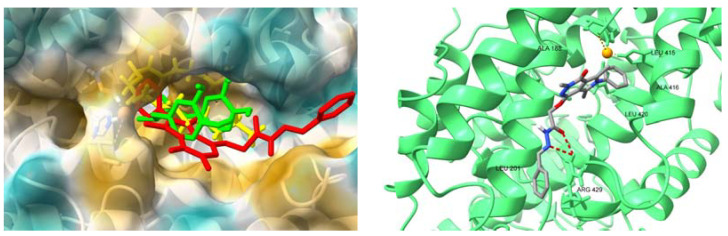
The location of **5b** (red), nordihydroguaiaretic acid (green), and ligand C8B (yellow) into the 15-LOX channel (**right**). Docking pose of **5b** (**left**). Hydrogen bonds are marked in red.

Figure 7 presents the position of compound **5b** (with the lowest binding energy; red), well-known natural 15-LOX inhibitors nordihydroguaiaretic acid (green) [51,52], and native ligand from 4NRE PDB structure, ligand C8B (yellow) [50]. Acid residues involved in the stabilization of the 15-LOX/**5b** complex are also shown. The complex is stabilized by the formation of two hydrogen bonds between Arg429 and oxygen and nitrogen atoms in *N*-acylhydrazone moiety (2.90 Å, 2.65 Å). The 6-phenyl-3,5,7-trimethylpyrrolo[3,4-*d*]pyridazinone core is oriented to the inside of the U-shape channel. Compound **5b** does not directly interact with catalytic iron or amino acid residues coordinated to Fe^2+^: His373, His378, His553, Ile676. Several hydrophobic interactions, π-alkyl, with Ala188, Ala416, Leu415, and Leu420 are observed. The analysis of these results suggests that the potential 15-LOX inhibitory activity of title NAH derivatives relies not on the redox mechanism but rather on competing with the substrate for binding to the active site of the enzyme [51,53,54,55,56].

### 2.4. Interactions with Plasma Proteins

After administration, drugs are bound and transported by blood proteins which affect their pharmacokinetics in vivo. Therefore, we performed experiments estimating the binding mode of title *N*-acylhydrazone derivatives with human serum albumin (HSA) and alpha-1-acid glycoprotein (AAG), which are the most abundant plasma proteins. The interactions between new compounds and HSA and AAG were monitored by optical techniques such as CD, fluorescence spectroscopy, and molecular docking studies [57]. Since the structures of all compounds are very similar and studies of interactions with proteins are time-consuming and laborious, we decided to conduct these preliminary experiments for derivatives from the **5** series **5a**-**5c**. Moreover, we decided to conduct a study of the interaction with plasma proteins for compounds **5a**-**5c**, i.e., those with unsubstituted phenyl ring in NAH moiety, because we found that the R^1^ substituent may potentially have the greatest impact on the interaction with macromolecules. This substituent most differentiates this group of compounds in terms of structure.

#### 2.4.1. The Fluorescence Spectroscopic Studies

**Figure 8:**
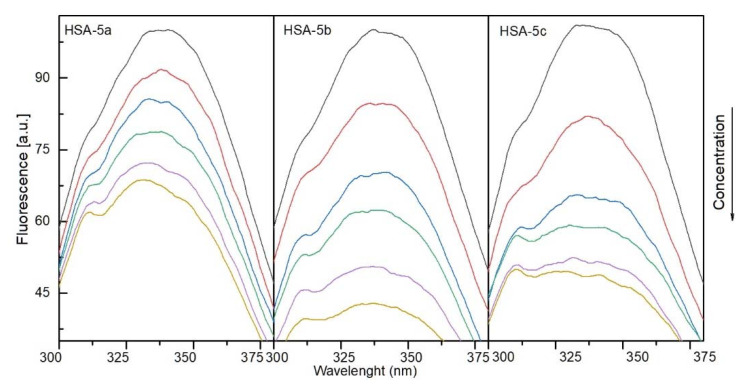
Fluorescence quenching spectra of human serum albumin (HSA) in the presence of different concentrations of compounds **5a**, **5b**, and **5c** (T = 297 K, λ_ex_ = 280 nm). The concentration of HSA was 1.0 µM, and the concentrations of **5a**, **5b**, and **5c** were 0, 0.4, 0.8, 1.2, 1.6, and 2.0 µM.

**Figure 9:**
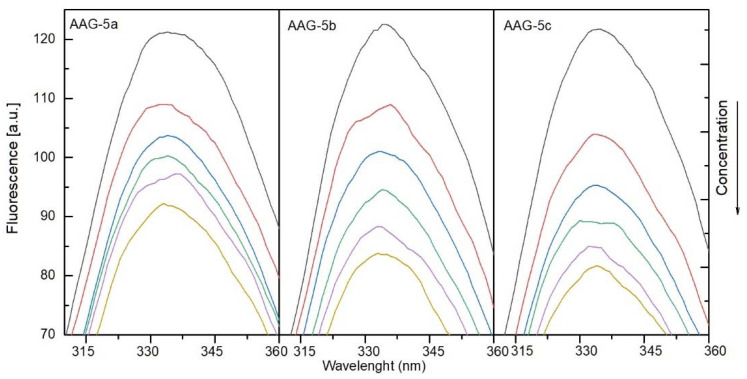
Fluorescence quenching spectra of alpha-1-acid glycoprotein (AAG) in the presence of different concentrations of compounds **5a**, **5b**, and **5c** (T = 297 K, λ_ex_ = 280 nm). The concentration of AAG was 1.0 µM, and the concentrations of **5a**, **5b**, and **5c** were 0, 0.4, 0.8, 1.2, 1.6, and 2.0 µM.

The investigation of fluorescence spectroscopy measurements allows us to characterize the interaction between the studied compounds and major blood plasma proteins, such as human serum albumin (HSA) and α1-acid glycoprotein (AAG). The sequences of these proteins contain the tryptophan (Trp), tyrosine (Tyr), and phenylalanine (Phe) residues [58,59]. These amino acids are directly responsible for the fluorescence effect, but the most significant and vulnerable impact comes from Trp residues. The fluorescence titration study is a commonly used method to evaluate the binding of small molecules with plasma proteins. The fluorescence quenching for HSA and AAG interaction is presented in Figure 8 and Figure 9, respectively.

After the addition of each portion of the studied compound to the protein solution, a decrease in fluorescence intensity was detected in all analyzed systems. Moreover, the slight shift of maximum peak occurred towards shorter wavelengths, and it is connected with variations in the chromophore surrounding [60]. Fluorescence quenching takes place due to the collision of the fluorophore and molecule diffuse in the solution (collide one with each other with a quencher but without complex formation) or due to the formation of a supramolecular complex, or due to the loss of energy of an excited state in non-radiation mode [61,62]. The fluorescence studies allow us to identify a kind of interaction mechanism in the solutions between protein and ligand, which may be dynamic or static. The temperature dependence experiments allow us to clarify which mechanism of interaction took place. The analysis was performed using the Stern–Volmer Equation (1) with inner filter correction (2) [62,63]:

(1) $$F_{corr}=F_{obs}10^{\frac{\left(A_{ex}+A_{em}\right)}{2}}$$

(2) $$\frac{F_{0}}{F}=1+k_{q}\tau \left[Q\right]=1+K_{SV}$$

where *F*_0_ is the steady-state fluorescence intensities at the maximum wavelength without a quencher, *F* is the steady-state fluorescence intensities at the maximum wavelength with the quencher, *k_q_* is the quenching rate constant of the molecule, *τ0* is the average lifetime of the molecule, [*Q*] is the concentration of quencher, *K_sv_* is the Stern–Volmer constant, *F_corr_* is the corrected fluorescence intensities, and *F_obs_* is the observed fluorescence intensities. *A_ex_* is the absorbance at excitation wavelengths, and *A_em_* is the absorbance at emission wavelengths.

The Stern–Volmer (*K_sv_*) and the quenching rate constants (*k_q_*) are obtained by linear fitting, and data are collected in Table 4 and Table 5, respectively, for HSA and AAG measurements. During the higher temperature, the values of *K_sv_* become lower. Moreover, the values of *k_q_* (~10^13^ dm^3^·mol^−1^·s^−1^) constants are higher than the maximum scattering collision quenching constant (2 × 10^10^ dm^3^·mol^−1^·s^−1^) [64]. These findings suggest that the interaction between blood plasma proteins (HSA, AAG) with **5a**, **5b**, and **5c** is a rather static quenching mechanism and indicates forming ground-state complexes.

**Figure 10:**
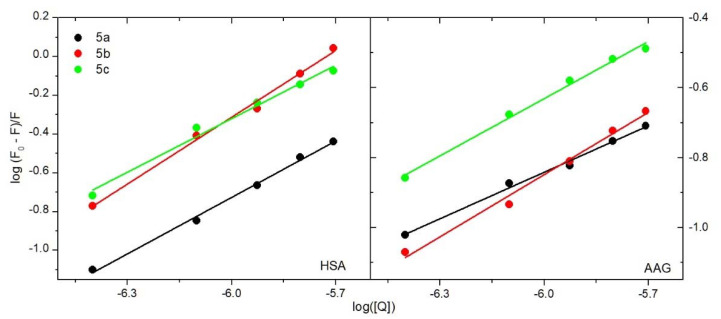
Double logarithm regression plots for quenching of human serum albumin (HSA) (**left**) and alpha-1-acid glycoprotein (AAG) (**right**) by compounds **5a**, **5b**, **5c**.

The further analysis of fluorescence data enables us to determine the number of the binding site (*n*) and binding constants (*K_b_*) by following Equation (3):

(3) $$log\frac{F_{0}-F}{F}=logK_{b}+nlog[Q]$$

where *F_0_* is fluorescence intensities at the maximum wavelength without a quencher, *F* is fluorescence intensities at the maximum wavelength with the quencher, both of them with respect to the inner filter effect correction, and the [*Q*] is the concentration of the quencher. The plots of double logarithm regression are fitting by linear function for all studied compounds (Figure 10).

The stoichiometry of the complexes formation converges to one, which means that only one binding site in protein is occupied by *N*-acylhydrazones. The values of binding constants (K_b_) for HSA complexes determined at 297 K with **5a** and **5c** are similar and equal to 1.26 × 10^5^ dm^3^mol^−1^ and 1.62 × 10^5^ dm^3^mol^−1^, respectively. However, for the HSA/5b complex, the value was determined to be 38.02 × 10^5^ dm^3^mol^−1^, which means that this compound exhibits a higher affinity to protein. With increasing temperature to 303 K and 308 K, the K_b_ constants decreased, which was observed in earlier drug–albumin interaction studies and confirms the ligand–protein complexes formation [65,66,67,68]. The formation of complexes with AAG is less thermodynamic stable than with has. The stability constants are presented in Table 5. The lowest affinity exhibits a **5a** compound where K_b_ = 6.61 dm^3^mol^−1^, while constants for complexes with **5b** and **5c** at 297 K are 51.28 dm^3^mol^−1^, 43.65 dm^3^mol^−1^, respectively. All studied *N*-acylhydrazones formed more stable complexes with HSA than with AAG.

The correlation between the stability constant of complexes *K_b_* and standard thermodynamic parameters enthalpy change (Δ*H*°), and entropy change (Δ*S*°) was determined by the van’t Hoff Equation (4):

(4) $$logK_{b}=-\frac{∆H^{{}^{\circ}}}{RT}+\frac{∆S^{{}^{\circ}}}{R}$$

where *R* is the universal gas constant; the free energy change (Δ*G*°) can be calculated by following Equation (5):

(5) $$∆G^{{}^{\circ}}=∆H^{{}^{\circ}}-T∆S^{{}^{\circ}}=-RTlnK_{b}$$

The binding mode can be provided by analysis of the thermodynamic parameters. The positive values of enthalpy and entropy (Δ*H*°, Δ*S*° > 0) support the hydrophobic character of interaction, or when enthalpy is near zero and the entropy is positive (Δ*H*°~0, Δ*S*° > 0), it indicates electrostatic contacts. The case where both enthalpy and entropy are negative values (Δ*H*°, Δ*S*° < 0) implies the van der Waals forces and hydrogen bonding [69,70]. Therefore, intermolecular complexes of *N*-acylhydrazones with proteins: HSA and AAG are mainly throughout van der Waals forces and weak hydrogen bonding, where the enthalpy and entropy established negative values (Table 4 and Table 5). Additionally, obtained results suggest that the binding of **5a**, **5b**, and **5c** with blood plasma proteins (HSA, AAG) are spontaneous processes due to the negative Gibbs free energy change (Δ*G*°) (Table 4 and Table 5).

#### 2.4.2. Binding Site Studies with HSA and AAG

**Figure 11:**
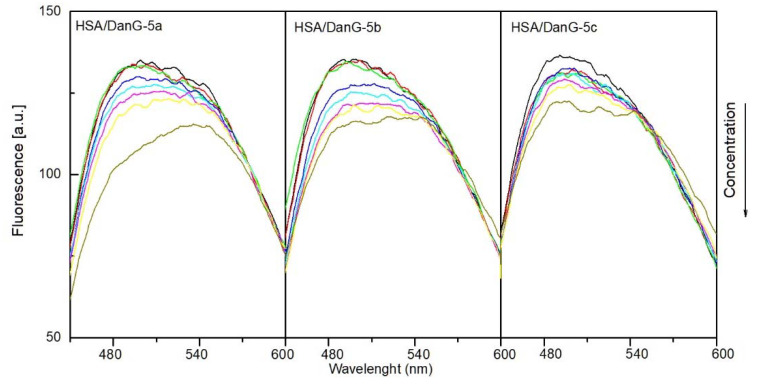
The fluorescence spectra for the human serum albumin (HSA), dansylated amino acids, and analyzed compounds **5a**, **5b**, and **5c**: HSA/DanG systems.

**Figure 12:**
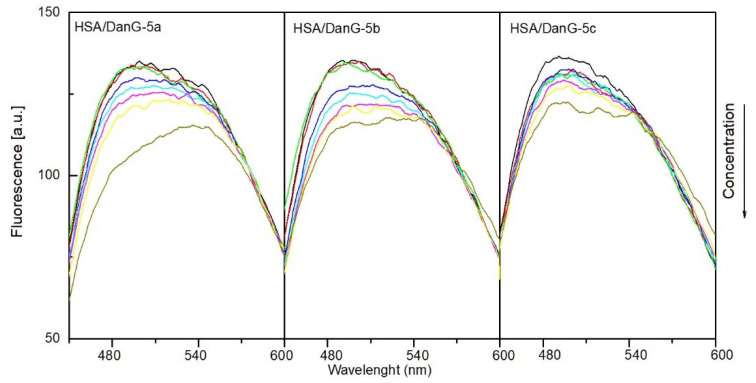
The fluorescence spectra for the human serum albumin (HSA), dansylated amino acids, and analyzed compounds **5a**, **5b**, and **5c**: HSA/DanF systems.

HSA, the most important transport protein in our body, has two main binding sites with high affinity to drug binding: drug site 1 in subdomain IIA and drug site 2 in subdomain IIIA [71,72,73]. To determine which site the test compounds bind to, the analysis with markers having an affinity for one of the binding sites can be used. Dansylated amino acids turn out to be good for this type of research [73]. In our study, we used dansyl-L-glycine (DanG) and dansyl-L-phenylalanine (DanF) as drug site 1 and drug site 2 markers, respectively. DanG and DanF bound with HSA show the phenomenon of fluorescence at the excitation wave equal to 350 nm. Under these conditions, the fluorescence spectrum is recorded with a band maximum at approximately 510 nm for DanG and 505 nm for DanF, respectively. We conducted a study in which we introduced successive portions of the analyzed compounds to a solution containing equimolar amounts of HSA and each marker. We increased the molar ratio of the reactants from 1:1:0 to 1:1:10. The spectra obtained for all six analyzed systems are shown in Figure 11 and Figure 12. The intensity of fluorescence decreases during the addition of successive portions of the analyzed compounds. This proves the replacement of the dansylated amino acid molecules by the tested compounds and confirms that **5a**, **5b**, and **5c** bind to the albumin molecule.

Based on the obtained results, it is possible to calculate the percentage of replacement of DanG or DanF in the complex with albumin by the tested compounds and thus compare the effectiveness of this reaction for all analyzed molecules. For this purpose, the following Formula (6) was used [72], and the results obtained are summarized in Table 6.

(6) $$percentage\;of\;replacement=\frac{F_{0}-F}{F_{0}}\cdot 100\%$$

From the results presented in Table 6, it can be seen that better replacement in HSA complexes is observed in the case of DanF. This means that the tested compounds bind with greater affinity to drug site 2 in the albumin molecule. The strongest impact was observed for the **5b** compound. In the case of DanG and binding to drug site 1, the obtained results are two times smaller. The **5c** binds the weakest to both binding sites.

Orosomucoid has seven binding sites in its molecule, but there is only one site where the drugs bind [74]. A very good fluorescent marker for binding site study in the case of AAG is quinalidine red (QR). Similar to the markers used for HSA, this marker exhibits a fluorescent phenomenon when bound to a protein molecule. When it is displaced from the complex with AAG by another compound, the fluorescence intensity decreases significantly [75].

**Figure 13:**
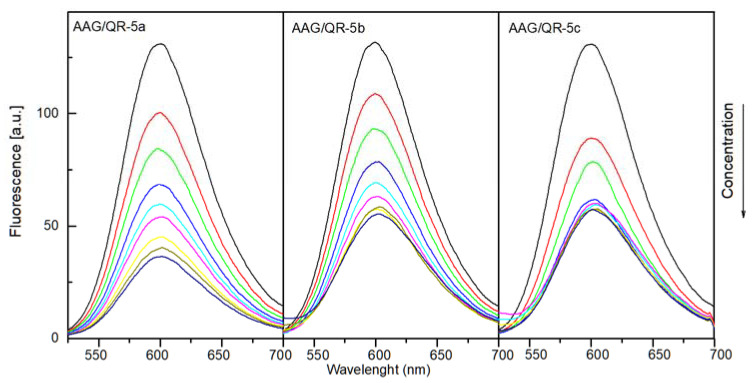
The fluorescence spectra for alpha-1-acid glycoprotein (AAG), quinalidine red, and analyzed compounds **5a**, **5b**, and **5c**.

Similar to HSA systems, we introduced successive portions of the analyzed **5a**, **5b**, and **5c** to a solution containing equimolar amounts of orosomucoid and QR. We used a 500 nm excitation wavelength. In these conditions, the maximum emission spectra were located near 600 nm. We increased the molar ratio of the reactants from 1:1:0 to 1:1:10. The spectra obtained for the three analyzed systems are shown in Figure 13. The intensity of fluorescence decreases during the addition of successive portions of the analyzed compounds. This proves the replacement of quinalidine red molecules by the tested compounds and confirms that **5a**, **5b**, and **5c** bind to the AAG.

In the case of testing the interaction of AAG with the tested compounds, the percentage of replacement of the QR marker in the complex with the protein by **5a**, **5b**, and **5c** was also calculated using Equation (6). Obtained results are collected in Table 7.

From the results presented above, it can be concluded that the tested compounds bind with good affinity to the drug site in the orosomucoid molecule. The strongest bond is for compound **5a**, while the weakest is for **5c**.

#### 2.4.3. Circular Dichroism Spectroscopy

**Figure 14:**
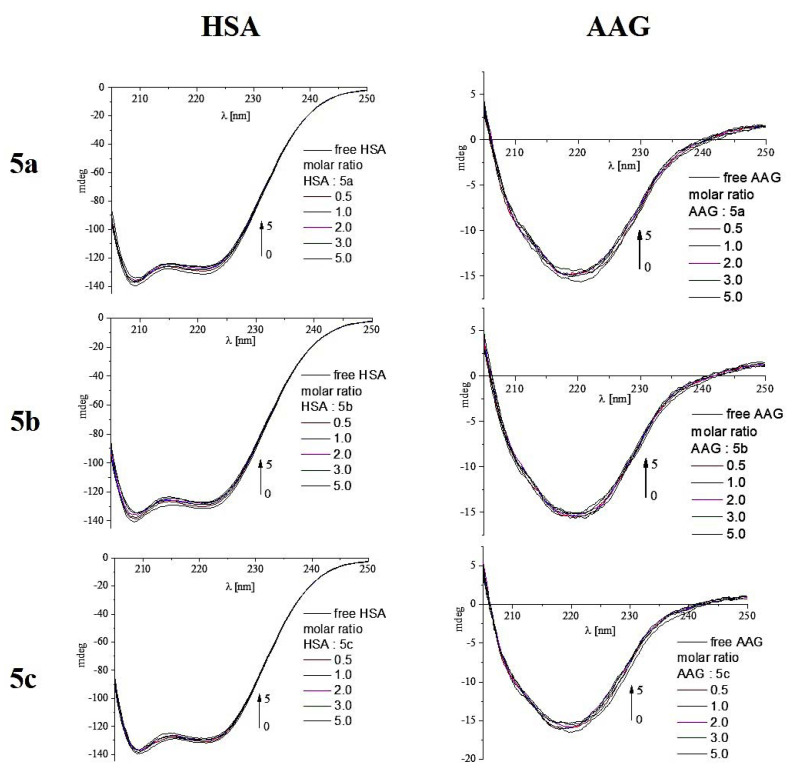
Circular dichroism spectra of the analyzed proteins: human serum albumin (HSA) and alpha-1-acid glycoprotein (AAG) in the absence and presence of analyzed compounds **5a**, **5b**, and **5c**.

When the small molecules interact with plasma proteins, changes in circular dichroism spectra are observed [76]. Specific secondary structures such as α-helix or β-sheet have a characteristic band on the spectrum [77]. For the first structure, two negative peaks near 209 and 220 nm are observed, while for the β-sheet, the negative band around 215 nm is present. We measured the CD spectra for proteins in the absence and presence of **5a**, **5b**, and **5c** (Figure 14). We analyzed the obtained results using the CD Multivariate SSE program, and they are summarized in Table 8 and Table 9.

As shown in Figure 14, the addition of each successive portion of the analyzed compounds influences the appearance of the CD spectra for the studied proteins. On the HSA spectra, two negative α-helix bands are present (Figure 14), which is typical for this protein. The observed changes during the addition of successive portions of the tested **5a**, **5b**, and **5c** are not significant. There is a slight reduction in the percentage of α-helix (Table 8), which is the dominant structure in protein. The largest change, equal to 2.4%, was observed for **5b,** and the smallest for **5c** (1.6%). However, it can be assumed that all analyzed compounds do not destabilize the protein secondary structure.

For AAG, one negative band near 220 nm is observed on the spectra (Figure 14). With the increase in the concentration of **5a**, **5b**, and **5c,** the course of the spectra changes slightly, which is confirmed by the analysis of the CD results (Table 9). The structure of the protein consists of about 30% of β-sheet and 20% of α-helix. Changes in the percentage of both forms do not exceed 2%. Loss of percentage of α-helix causes an increase in the content of β-sheet. The greatest changes can be observed for compound **5c**. However, similar to the HSA systems, it can be concluded that the binding of **5a**, **5b**, and **5c** to the AAG does not significantly affect its structure.

#### 2.4.4. Interactions with HSA and AAG–Molecular Docking Studies

Fluorescence studies in the presence of the binding site markers DanG and DanF indicate that compounds **5a**-**c** have a greater affinity for binding site 2 in subdomain IIIA. To determine how **5a**-**c** interact with HSA in the pocket of site 2, molecular docking studies were performed. The crystal structure of human serum albumin with co-crystalized dansyl-L-phenylalanine (PDB entry 2XW0 [73]) was used for docking. The binding affinity for interactions of compounds **5a**-**c**, and also **6a**-**c**, and **7a**-**c** with HSA, are listed in Table 10. For all compounds, the stable complex with HSA is formed with negative binding affinity. The calculated values are in the range from −8.2 kcal/mol to −9.3 kcal/mol. The differences are not major. The stability of the complex is improved by the phenyl group instead of the alkyl chain and the substituents on the aromatic ring.

**Figure 15:**
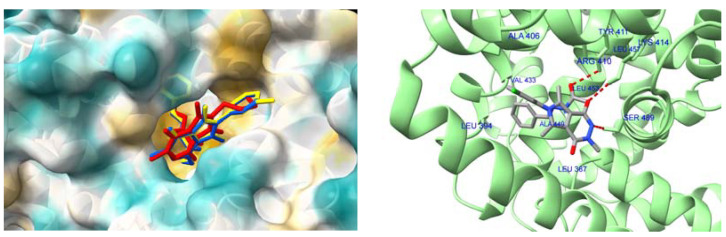
The position of **5a** (yellow), **5b** (blue), and **5c** (red) into human serum albumin (HSA) site 2 (**left**) and docking pose of **5c** (**right**). Hydrogen bonds are marked in red.

The position of compounds **5a**-**c** into a binding pocket, and the docking pose of **5c** are presented in Figure 15.

Three hydrogen bonds are found: Lys414 with an oxygen atom (2.82 Å), Tyr411 with a carbonyl group of *N*-acylhydrazone moiety (3.15 Å), and Ser489 with nitrogen atom N5 of pyrrolo[3,4-*d*]pyridazinone scaffold (2.85 Å). Several hydrophobic interactions are observed. The phenyl ring of the *N*-acylhydrazone group and 6-(3-chlorophenyl)-3,5,7-trimethylpyrrolo[3,4-*d*]pyridazinone moiety, interacts by π-alkyl contacts with Val433, Ala449, Leu453 and Leu394, Ala406, Arg410, respectively. Moreover, π-cation interactions between Arg410 and pyrrolo[3,4-*d*]pyridazinone core are found. Compounds **5a** and **5b** interact with HSA into site 2 in a very similar way. Hydrogen bonds with Lys414, Tyr 411, and Ser489 are found. The π-alkyl, π-cation contacts with pyrrolo[3,4-*d*]pyridazinone and *N*-acylhydrazone groups are observed.

**Figure 16:**
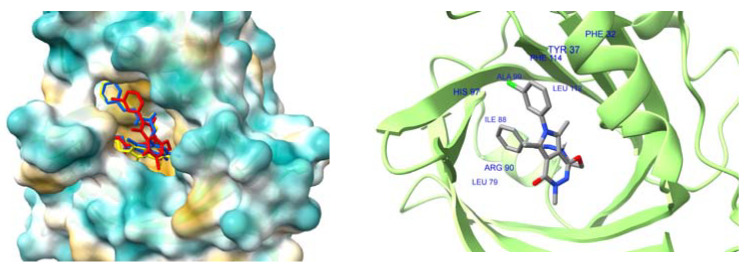
The position of **5a** (yellow), **5b** (blue), and **5c** (red) into the alpha-1-acid glycoprotein (AAG cavity) (**right**) and docking pose of **5c** (**left**).

To determine the mechanism of interaction between **5a**-**c** compounds and α1-acid glycoprotein, the crystal structure PDB entry 3KQ0 [78] was used for docking. The large AAG cavity is divided into three partially overlapping subsites, where basic, acidic, and neutral drugs can bind. Molecular docking showed that for all compounds, the binding affinity is negative (Table 10). The differences are not major. The calculated values are in the range from −8.5 kcal/mol to −9.7 kcal/mol. Series **5** forms slightly more stable complexes than series **6** and **7**, and series **b**, **c** then series **a**. The location of **5a**-**c** into the cavity and the docking pose of compound **5c** (the most negative binding affinity) are presented in Figure 16.

No hydrogen bonds were found. The phenyl ring of *N*-acylhydrazone moiety is involved in the π-alkyl interactions with Leu79, Leu112, Ile88, and Ala99. A 3-chlorophenyl substituent interacts by π-π stacked contacts with Tyr37 and Phe32 and via π-alkyl interactions with His97 and Phe114. Complex AAG-**5c** is additionally stabilized by π-cation interactions with Arg90.

### 2.5. In Silico Absorption Distribution Metabolism and Excretion (ADME) Pharmacokinetic and Drug-Likeness Predictions

The SwissADME server (http://www.swissadme.ch/ accessed on 25 May 2023) was used to predict the pharmacokinetic properties and drug-likeness of the *N*-acylhydrazone derivatives **5a**-**c**–**7a**-**c**.

The physicochemical characteristics of new compounds with reference to Lipinski’s rule of five (Ro5) are shown in Table 11. All studied compounds meet the conditions of the Ro5 without any violation. These results indicate that title derivatives could probably show good oral bioavailability and membrane permeability [79].

Data collected in Table 12 clearly show that all compounds are expected to be highly absorbed through the gastrointestinal tract, but they are not able to cross the blood-brain barrier (BBB). Their water solubility is rather moderate.

All studied derivatives do not violate the descriptors of Veber’s rule. Moreover, low values of topological polar surface area (TPSA) (<140 Å^2^) and promising bioavailability scores indicate their good membrane permeability (Table 13) [80,81].

**Figure 17:**
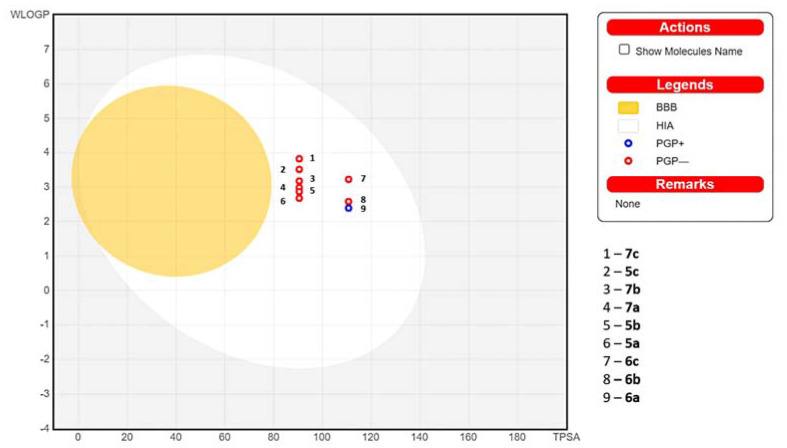
The BOILED-Egg diagram (generated by the SwissADME server accessed on 25 May 2023) for all investigated *N-*acylhydrazone derivatives of pyrrolo[3,4-*d*]pyridazinone.

Figure 17 presents the BOILED-Egg diagram for all investigated compounds. Molecules located in the BOILED-Egg’s white are supposed to be passively absorbed in the GI system, while those located in the yolk are able to passively permeate through the BBB.

## 3. Discussion

The objective of the present study was the synthesis, in vitro and in silico evaluation of the biological activity of new series of *N*-acylhydrazone derivatives of pyrrolo[3,4-*d*]pyridazinone. All compounds did not show cytotoxicity in the carried-out MTT assay. Therefore, their anti-inflammatory activity and pharmacokinetic properties could have been further examined using enzymatic, computational, and spectroscopic techniques.

According to the results of in vitro and molecular docking studies, title *N*-acylhydrazone derivatives showed promising dual COX/LOX inhibitory activity comparable to the commonly known and approved anti-inflammatory drugs used as a reference. In the performed enzymatic assay, all examined compounds, besides **7c**, inhibited both isoforms of cyclooxygenase. However, their affinity towards the inducible form COX-2 was significantly higher than COX-1. The abovementioned derivative **7c** acted as a selective COX-2 inhibitor. The COX-1 inhibitory activity of studied compounds was similar to meloxicam. Nevertheless, it is worth noticing that they inhibited the COX-2 isoform more efficiently than this reference drug. Consequently, new derivatives are characterized by a much better COX-2/COX-1 selectivity index than meloxicam and diclofenac. On the other hand, the COX-2 inhibitory activity of novel compounds was lower compared to celecoxib. These results are supported by molecular docking studies, which showed that for all compounds, the binding affinity is negative, indicating the formation of stable complexes. For interactions with COX-2, where the active pocket is slightly larger than in the case of COX-1, the values of binding energy were lower than for interactions with the constitutive isoform of cyclooxygenase. This stays in good agreement with the results of the enzymatic assay. For interactions with both COX isoenzymes, the best results were obtained for compounds of series **6**, with 2-OH substituent in the phenyl ring of *N*-acylhydrazone moiety. This hydroxyl group is engaged in the formation of hydrogen bonds inside the active site of the enzyme. Moreover, derivatives with a 3-chlorophenyl substituent in position 6 of the pyrrolo[3,4-*d*]pyridazinone core (series **c**) form more stable complexes than those with phenyl or n-butyl substituent. These findings correlate well with the results of in vitro experiment, where derivative **6c** is characterized by the lowest IC_50_ values. Title molecules take a position in the binding pocket of COX-1 and COX-2, very similar to meloxicam, and the formed complex is stabilized by interactions of amino acids with pyrrolo[3,4-*d*]pyridazinone scaffold and *N*-acylhydrazone moiety. Moreover, binding with the active site of COX-2 is additionally stabilized by the formation of hydrogen bonds.

When analyzing these results, we can notice some significant differences in COX inhibitory activity of title *N*-acylhydrazones and previously reported 1,3,4-oxadiazole and 1,2,4-triazole derivatives of pyrrolo[3,4-*d*]pyridazinone. First, only one *N*-acylhydrazone derivative, **7c**, did not show affinity towards the constitutive isoform COX-1. In the case of 1,3,4-oxadiazole and 1,2,4-triazole derivatives, there was a higher percentage of selective COX-2 inhibitors. As mentioned above, the active site of COX-1 is smaller than that of COX-2 and, therefore, does not bind large molecules. As a consequence, *N*-acylhydrazones can have easier access to the binding pocket of the COX-1 enzyme because these compounds are smaller, less spacious, and more labile than rather rigid 1,3,4-oxadiazole and 1,2,4-triazole analogs. Moreover, title compounds are characterized by better COX-2 inhibitory activity and a superior COX-2/COX-1 inhibitory ratio than 1,3,4-oxadiazole and 1,2,4-triazole derivatives of pyrrolo[3,4-*d*]pyridazinone. These can be explained again by the fact that the molecules presented in the current study are smaller, and their complexes with COX-2 are additionally stabilized by hydrogen bonds formed with, among others, acylhydrazone moiety.

The ability of new *N*-acylhydrazones to inhibit 15-LOX was also estimated. According to the enzymatic assay, their activity was comparable to zileuton, which is so far the only approved 15-LOX inhibitor. The results of molecular docking studies show that the values of binding energy for all compounds are negative. Examined derivatives take a position in the active site of 15-LOX, similar to natural inhibitor nordihydroguaiaretic acid. Moreover, the complex is additionally stabilized by the formation of two hydrogen bonds between Arg429 and oxygen and nitrogen atoms in an *N*-acylhydrazone moiety. Nevertheless, title compounds do not interact with the catalytic iron atom Fe^2+^ in the binding pocket of the enzyme. Their 15-LOX inhibitory activity relies not on the redox mechanism but on competing with the natural ligand for the binding site of the enzyme.

Drugs administered orally are absorbed in the GI system and transported with blood proteins before they reach their molecular target. Cyclooxygenases and lipoxygenases are enzymes located intracellularly, so COX/LOX inhibitors should have good membrane permeability. Therefore the binding mode of title compounds with HSA and AAG and some of their pharmacokinetic properties were described in this paper.

The performed spectroscopic studies demonstrated that *N*-acylhydrazone derivatives of pyrrolo[3,4-*d*]pyridazinone interact with blood plasma proteins—HSA and AAG—in a static quenching mechanism forming ground-state complexes in the stoichiometry converging to one, which indicates that only one binding site in protein is occupied by the compound. The *N*-acylhydrazones interact with these proteins mainly through the van der Waals forces and weak hydrogen bonding. The complexes formed with HSA are more thermodynamic stable than those with AAG. Moreover, CD spectroscopic investigations revealed that the binding of title compounds with HSA and AAG neither significantly affects nor destabilizes the proteins’ secondary structure. Molecular docking studies confirmed that the analyzed derivatives form stable complexes with HSA and AAG with negative binding affinity. These results can suggest good distribution and potentially a long half-life in vivo of title N-acylhydrazones.

Moreover, according to the computational predictions carried out, all studied compounds fulfilled the descriptors of Lipinski’s Ro5 and Veber rule. Therefore they are expected to be easily absorbed through the GI tract, but they are not supposed to cross the BBB. Additionally, low values of TPSA of new *N*-acylhydrazone derivatives of pyrrolo[3,4-*d*]pyridazinone can suggest their good membrane permeability.

Taking all the above into consideration, we can suppose that due to the significant anti-COX activity and promising COX-2/COX-1 inhibitory ratio, the *N*-acylhydrazone derivatives of pyrrolo[3,4-*d*]pyridazinone could probably serve as potent anti-inflammatory agents deprived of adverse effects related to the GI and CV systems. Moreover, dual COX/LOX inhibitory activity may contribute to the enhanced anti-inflammatory activity and safety profile of these drugs because inhibition of the LOX reduces the risk of bronchoconstriction and the occurrence of AIA. According to pharmacokinetic and drug-likeness predictions, we can expect that these compounds could be administered orally. Nevertheless, all these hypotheses can only be verified by in vivo experiments planned in the future.

Moreover, it is worth mentioning that *N*-acylhydrazone derivatives could be easily further modified into new series of compounds. Cyclic analogs of the title derivatives could be received in reaction with, for example, acetic or thioglycolic acid or with chloramine T. Such modifications may be helpful in the development of the next series of anti-inflammatory derivatives of pyrrolo[3,4-*d*]pyridazinone.

## 4. Materials and Methods

### 4.1. Chemistry

#### 4.1.1. Instrumentation and Chemicals

All the chemicals, solvents, and reagents necessary for synthesis, purification, and other experiments were delivered by commercially available suppliers (Alchem, Wrocław, Poland; Chemat, Gdańsk, Poland; Archem, Łany, Poland) and were used without any further purification. All dry solvents were received according to the standard procedures. The reactions’ progress was monitored using the thin-layer chromatography (TLC) technique on silica-gel-60-F254-coated TLC plates. Ethyl acetate or ethyl acetate:methanol (9:1) mixtures were used as eluents. The plates were analyzed in UV light at 254 or 366 nm. The melting points of new compounds were determined on the Electrothermal Mel-Temp 1101D apparatus (Cole-Parmer, Vernon Hills, IL, USA) using the open capillary method and were uncorrected. The ^1^H NMR (300 MHz) and ^13^C NMR (75 MHz) spectra were recorded on the Bruker 300 MHz NMR spectrometer (Bruker Analytische Messtechnik GmbH, Rheinstetten, Germany). The samples were dissolved in CDCL_3_ or DMSO-*d*_6_. Tetramethylsilane (TMS) was used as an internal reference, and chemical shifts (δ) were reported in parts per million (ppm). The mass spectra (MS) were recorded using the Bruker Daltonics Compact ESI-Mass Spectrometer (Bruker Daltonik, GmbH, Bremen, Germany), operating in the positive ion mode. The analyzed compounds were dissolved in a methanol–chloroform mixture. The infrared (IR) spectra were determined on the Nicolet iS50 FT-IR Spectrometer (Thermo Fisher Scientific, Waltham, MA, USA). The samples were applied as solids, and the frequencies were reported in cm^−1^. The BIOVIA Draw 2021 was used to draw 2D structures of synthesized compounds. All newly reported derivatives were purified by crystallization or using CC and were determined to have purity over 95% unless stated otherwise.

#### 4.1.2. Chemical Synthesis

The synthetic protocols and experimental data for compounds **1a**-**c, 2a**-**b–4a**-**b,** and all other intermediates have been reported in our previous papers [36,42,43,44].

##### The Synthetic Procedure for 6-(3-chlorophenyl)-3,5,7-trimethyl-2*H*-pyrrolo[3,4-*d*]pyridazine-1,4-dione (**2c**):

1-(3-chlorophenyl)-2,5-dimethyl-3,4-pyrroledicarboxylic acid anhydride **1c** (20 mmol) was suspended in 50 mL of acetonitrile in a round bottom flask, and the mixture was warmed up till the anhydride dissolved. Subsequently, 2.62 mL (50 mmol) of methylhydrazine was added, and the mixture was refluxed for 5 h and left overnight. The formed white precipitate was filtered off and washed with cold ethanol, giving the desired 6-(3-chlorophenyl)-3,5,7-trimethyl-2H-pyrrolo[3,4-d]pyridazine-1,4-dione **2c**.

- **2c:** 6-(3-chlorophenyl)-3,5,7-trimethyl-2-[H]-pyrrolo[3,4-*d*]pyridazine-1,4-dione

Yield: 66.69%; m.p.: 272–273 °C;

FT-IR (selected lines, γ_max_, cm^−1^): 3049 (C-H arom.), 2919 (C-H aliph.), 1615 (C=O); ^1^H NMR (300 MHz, DMSO-*d*_6_) δ: 2.26 (s, 3H, 7-CH3), 2.29 (s, 3H, 5-CH_3_), 3.29 (s, 3H, 3-CH_3_), 7.40–7.41 (m, 1H, Ar-H), 7.61–7.66 (m, 3H Ar-H); ^13^C NMR (75 MHz, DMSO-*d*_6_) δ: 11.53, 111.55, 127.44, 128.50, 130.04, 131.78, 134.38, 137.69; HR-MS (ESI-MS) (*m*/*z*): calcd. for C_15_H_14_ClN_3_O_2_ [L+H]^+^: 304.0847; found: 304.0837;

##### The Synthetic Procedure for methyl 2-[6-(3-chlorophenyl)-3,5,7-trimethyl-4-oxo-Pyrrolo[3,4-*d*]pyridazin-1-yl]oxyacetate (**3c**):

The mixture of 6-(3-chlorophenyl)-3,5,7-trimethyl-2H-pyrrolo[3,4-*d*]pyridazine-1,4-dione **2c** (10 mmol) and K_2_CO_3_ (20 mmol) was suspended in 50 mL of acetonitrile in a round bottom flask. Then, ~1 mL of methyl chloroacetate (11 mmol) was added, and the mixture was refluxed for about 5 h. Afterward, the solvent was removed in a vacuum. The crude solid was dissolved in chloroform and filtered. The excess of CHCl_3_ was distilled off under reduced pressure, and the residue was purified by CC using ethyl acetate as an eluent. The fractions containing product **3c** (R_f_ = 0.74) were combined and evaporated to give a white-yellowish solid of methyl 2-[6-(3-chlorophenyl)-3,5,7-trimethyl-4-oxo-pyrrolo[3,4-*d*]pyridazin-1-yl]oxyacetate **3c**.

- **3c:** Methyl 2-[6-(3-chlorophenyl)-3,5,7-trimethyl-4-oxo-pyrrolo[3,4-*d*]pyridazin-1-yl]oxyacetate

Yield: 72.77%; m.p.: 128–130 °C;

FT-IR (selected lines, γ_max_, cm^−1^): 3049, 3028 (C-H arom.), 2938, 2919, (C-H aliph.); 1762, 1639 (C=O); ^1^H NMR (300 MHz, DMSO-*d*_6_) δ: 2.27 (s, 3H, 7-CH_3_), 2.34 (s, 3H, 5-CH_3_), 3.38 (s, 3H, 3-CH_3_), 3.69 (s, 3H, (CO)-*O*-CH_3_), 4.87 (s, 2H, *O*-CH_2_), 7.42–7.44 (m, 1H, Ar-H), 7.62–7.68 (m, 3H, Ar-H); ^13^C NMR (75 MHz, DMSO-*d*_6_) δ: 11.50, 12.09, 37.17, 52.23, 62.91, 108.37, 111.67, 124.67, 127.45, 130.15, 130.53, 131.84, 134.42, 137.80, 148.10, 158.35, 169.21 HR-MS (ESI-MS) (*m*/*z*): calcd. for C_18_H_18_ClN_3_O_4_ [L+H]^+^: 376.1059; found: 376.1057;

##### The Synthetic Procedure for 2-[6-(3-chlorophenyl)-3,5,7-trimethyl-4-oxo-pyrrolo[3,4-*d*]pyridazin-1-yl]oxyacetohydrazide **(4c)**:

Methyl ester derivative **3c** (10 mmol) was refluxed with an excessive amount of hydrazine hydrate (98%) (~5 mL; 100 mmol) in 75 mL of absolute ethanol for 3 h. The formed precipitate was filtered off, washed carefully with cold ethanol, dried, and purified by crystallization from this solvent giving a white, crystalline solid of 2-[6-(3-chlorophenyl)-3,5,7-trimethyl-4-oxo-pyrrolo[3,4-*d*]pyridazin-1-yl]oxyacetohydrazide **4c**.

- **4c:** 2-[6-(3-chlorophenyl)-3,5,7-trimethyl-4-oxo-pyrrolo[3,4-*d*]pyridazin-1-yl]oxyacetohydrazide

Yield: 84.12%; m.p.: 167–169 °C;

FT-IR (selected lines, γ_max_, cm^−1^): 3331, 3248 (*N*H_2_ and *N*H), 3042, 3019 (C-H arom.), 2935 (C-H aliph.) 1629 (C=O); ^1^H NMR (300 MHz, DMSO-*d*_6_) δ:2.26 (s, 3H, 7-CH_3_), 2.33 (s, 3H, 5-CH_3_), 3.40 (s, 3H, 3-CH_3_), 4.33–4.35 (m, 2H, -*N*H_2_), 4.63 (s, 2H, *O*-CH_2_), 7.39–7.41 (m, 1H, Ar-H), 7.62–7.67 (m, 3H, Ar-H) 9.22 (s, 1H, *N*H); ^13^C NMR (75 MHz, DMSO-*d*_6_) δ: 11.50, 12.16, 37.22, 64.16, 108.99, 111.77, 124.66, 127.42, 128.43, 130.07, 131.84, 134.39, 137.93, 149.01, 158.41, 167.05; HR-MS (ESI-MS) (*m*/*z*): calcd. for C_17_H_18_ClN_5_O_3_ [L+H]^+^: 376.1171; found: 376.1154;

##### The General Procedure for Preparation of Title *N*-acylhydrazones **(5a-c–7a-c)**

Hydrazide derivatives **4a**-**c** (2 mmol) were suspended in 25 mL of ethanol with a catalytic amount of acetic acid. The mixture was warmed up in order to dissolve the substrate. Subsequently, an equimolar quantity of adequate aromatic aldehyde was added. The mixture was refluxed for about 2–3 h till the substrates disappeared on TLC. After cooling, the desired *N*-acylhydrazone precipitate was filtered off, washed with ethanol, and crystallized from the appropriate solvent.

- **5a**: *N*-[(*Z*/*E*)-benzylideneamino]-2-(6-butyl-3,5,7-trimethyl-4-oxo-pyrrolo[3,4-*d*]pyridazin-1-yl)oxy-acetamide

Yield: 85.67% m.p.: 210–212 °C

FT-IR (selected lines, γmax, cm−1): 3209 (-*N*H), 3070 (C-H arom.), 2956, 2931 (C-H aliph.), 2868 (C-H azomethine), 1683, 1620 (C=O), 1549 (C=N); ^1^H NMR (300 MHz, DMSO-*d*_6_) δ: 0.92–0.94 (t, 3H, CH_3_-CH_2_-CH_2_-CH-), 1.31–1.33 (m, 2H, CH_3_-CH_2_-CH_2_-CH-), 1.58 (m, 2H, CH_3_-CH_2_-CH_2_-CH-), 2.53–2.54 (s, 3H, 7-CH_3_), 2.58–2.59 (s, 3H, 5-CH_3_), 3.32–3.33 (s, 3H, 3-CH_3_), 3.97 (m, 2H, CH_3_-CH_2_-CH_2_-CH-), 4.76 (*trans*) and 5.24 (*cis*) (2s, 2H, -*O*-CH_2_-); 7.41 (m, 3H, Ar-H), 7.65–7.66 (m, 2H, Ar-H), 7.99 (*trans*) and 8.22 (*cis*) (2s, 1H, *N*=CH), 11.54 (*trans*, *cis*) (s, 1H, *N*H) ^13^C NMR (75 MHz, DMSO-*d*_6_*)* δ: 10.65, 11.28, 13.99, 19.92, 32.06, 32.06, 37.14, 43.80, 62.93, 108.29, 111.29, 123.40, 127.28, 127.53, 129.00, 129.28, 130.39, 134.43, 144.10, 158.46, 169.12; HR-MS (ESI-MS) (*m*/*z*): calcd. for C_22_H_27_N_5_O_3_ [L+H]^+^: 410.2187; found: 410.2205;

- **6a**: 2-(6-butyl-3,5,7-trimethyl-4-oxo-pyrrolo[3,4-*d*]pyridazin-1-yl)oxy-*N*-[(*Z*/*E*)-(2-hydroxyphenyl)methyleneamino]acetamide

Yield: 84.91% m.p.: 225–227 °C;

FT-IR (selected lines, γmax, cm^−1^): 3223 (-*N*H), 3167 (-OH), 3051 (C-H arom.), 2963, 2933 (C-H aliph.), 2860 (C-H azomethine), 1703, 1616 (C=O), 1547 (C=N); ^1^H NMR (300 MHz, DMSO-*d*_6_) δ: 0.88–0.93 (t, 3H, CH_3_-CH_2_-CH_2_-CH-), 1.28–1.35 (m, 2H, CH_3_-CH_2_-CH_2_-CH-), 1.53–1.63 (m, 2H, CH_3_-CH_2_-CH_2_-CH-), 2.48 (s, 3H, 7-CH_3_), 2.58 (s, 3H, 5-CH_3_), 3.35 (s, 3H, 3-CH_3_), 3.94–3.98 (s, 2H, CH_3_-CH_2_-CH_2_-CH-), 4.79 (*trans*) and 5.21 (*cis*) (2s, 2H, -*O*-CH_2_-); 6.82–6.91 (m, 2H, Ar-H), 7.20–7.30 (m,1H, Ar-H), 7.50–7.53 (*trans*) and 7.64–7.66 (*cis*) (2m, 1H, Ar-H), 8.29 (*trans*) and 8.44 (*cis*) (2s, 1H, *N*=CH), 10.03 (*trans*) and 11.02 (*cis*) (2s, 1H, Ar-*O*H), 11.48 (*trans*) and 11.81 (*cis*) (2s, 1H, *N*H); ^13^C NMR (75 MHz, DMSO-*d*_6_) δ: 10.64, 11.27, 11.32, 13.98, 19.91, 32.05, 37.13, 43.789, 62.92, 64.02, 108.03, 108.27, 111.243, 111.29, 116.59, 116.81, 119.09, 119.87, 120.48, 123.39, 123.53, 126.78, 128.98, 129.12, 129.71, 131.62, 131.87, 141.70, 147.94, 148.56, 148.70, 156.84, 157.75, 158.46, 164.40, 168.77; HR-MS (ESI-MS) (*m*/*z*): calcd. for C_22_H_27_N_5_O_4_ [L+H]^+^: 426.2136; found: 426.2145

- **7a**: 2-(6-butyl-3,5,7-trimethyl-4-oxo-pyrrolo[3,4-*d*]pyridazin-1-yl)oxy-*N*-[(*Z*/*E*)-*p*-tolylmethyleneamino]acetamide

Yield: 82.03% m.p.: 209–211 °C

FT-IR (selected lines, γmax, cm^−1^): 3208 (*N*H), 3046 (C-H arom.), 2961, 2932 (C-H aliph.), 2857 (C-H azomethine), 1694, 1616 (C=O), 1547 (C=N); ^1^H NMR (300 MHz, DMSO-*d*_6_) δ: 0.88–0.93 (t, 3H, CH_3_-CH_2_-CH_2_-CH-), 1.32–1.38 (m, 2H, CH_3_-CH_2_-CH_2_-CH-), 1.53–1.63 (m, 2H, CH_3_-CH_2_-CH_2_-CH-), 2.31 (s, 3H, Ar-CH_3_), 2.48 (s, 3H, 7-CH_3_), 2.58 (s, 3H, 5-CH_3_), 3.35 (s, 3H, 3-CH_3_), 3.93–3.98 (s, 2H, CH_3_-CH_2_-CH_2_-CH-), 4.75 (*trans*) and 5.23 (*cis*) (2s, 2H, -*O*-CH_2_-); 7.21–7.24 (m, 2H, Ar-H), 7.53–7.58 (m, 2H, Ar-H), 7.95 (*trans*) and 8.18 (*cis*) (2s, 1H, *N*=CH), 11.47 (*trans*) and 11.52 (*cis*) (2s, 1H, *N*H); ^13^C NMR (75 MHz, DMSO-*d*_6_) δ: 10.64, 11.27, 13.99, 19.92, 21.46, 32.06, 37.14, 43.79, 108.29, 111.29, 123.39, 127.25, 127.53, 128.99, 129.88, 129.96, 131.74, 140.16, 144.17, 148.72, 158.46, 169.00; HR-MS (ESI-MS) (*m*/*z*): calcd. for C_23_H_29_N_5_O_3_ [L+H]+: 424.2343; found: 424.2363;

- **5b:** *N*-[(*Z*/*E*)-benzylideneamino]-2-(3,5,7-trimethyl-4-oxo-6-phenyl-pyrrolo[3,4-*d*]pyridazin-1-yl)oxy-acetamide

Yield: 82.30% m.p.: 274–275 °C

FT-IR (selected lines, γmax, cm^−1^): 3221 (*N*H), 3103 (C-H arom.), 2970, 2907 (C-H aliph.), 2854 (C-H azomethine), 1702, 1616 (C=O), 1545 (C=N); ^1^H NMR (300 MHz, DMSO-*d*_6_) δ: 2.27 (s, 3H, 7-CH_3_), 2.31 (s, 3H, 5-CH_3_), 3.36 (s, 3H, 3-CH_3_), 4.79 (*trans*) and 5.28 (*cis*) (2s, 2H, -*O*-CH_2_-); 7.41 (m, 5H, Ar-H), 7.58–7.67 (m, 5H, Ar-H), 7.99 (*trans*) and 8.23 (*cis*) (2s, 1H, *N*=CH), 11.55 (*trans*) and 11.59 (*cis*) (2s, 1H, *N*H); ^13^C NMR (75 MHz, DMSO-*d_6_*) δ: 11.54, 12.12, 37.25, 63.22, 64.68, 108.94, 111.86, 124.50, 127.29, 127.57, 128.32, 129.28, 129.86, 130.07, 130.27, 130.39, 134.96, 136.66, 143.96, 147.36, 148.82, 158.55, 164.63, 169.00; HR-MS (ESI-MS) (*m*/*z*): calcd. for C_24_H_23_N_5_O_3_ [L+H]^+^: 430.1874; found: 430.1892;

- **6b:** *N*-[(*Z*/*E*)-(2-hydroxyphenyl)methyleneamino]-2-(3,5,7-trimethyl-4-oxo-6-phenyl-pyrrolo[3,4-*d*]pyridazin-1-yl)oxy-acetamide

Yield: 85.87% m.p.: 214–217 °C

FT-IR (selected lines, γmax, cm^−1^): 3225 (*N*H), 3133 (*O*H), 3062 (C-H arom.), 2909 (C-H aliph.), 2856 (C-H azomethine), 1712, 1615 (C=O), 1544 (C=N); ^1^H NMR (300 MHz, DMSO-*d*_6_) δ: 2.27 (s, 3H, 7-CH_3_), 2.32 (s, 3H, 5-CH_3_), 3.37 (s, 3H, 3-CH_3_), 4.82 (*trans*) and 5.24 (*cis*) (2s, 2H, -*O*-CH_2_-); 6.88–6.90 (m, 2H, Ar-H), 7.20–7.28 (m, 1H, Ar-H), 7.38–7.74 (m, 2H, Ar-H), 7.50–7.57 (m, 4H, Ar-H), 8.29 (*trans*) and 8.45 (*cis*) (2s, 1H, *N*=CH), 10.03 (*trans*) and 11.01 (*cis*) (2s, 1H, Ar-*O*H), 11.49 (*trans*) and 11.82 (*cis*) (2s, 1H, *N*H); ^13^C NMR (75 MHz, DMSO-*d*_6_) δ: 11.54, 12.11, 12.16, 37.24, 62.98, 64.27, 108.53, 108.73, 111.73, 116.59, 116.83, 119.06, 119.80, 119.88, 120.48, 124.49, 124.73, 126.73, 128.31, 129.68, 129.89, 130.07, 130.16, 130.28, 131.63, 131.88, 136.51, 141.84, 148.09, 148.86, 156.60, 157.73, 158.48, 164.38, 169.00; HR-MS (ESI-MS) (*m*/*z*): calcd. for C_24_H_23_N_5_O_4_ [L+H]^+:^ 446.1823; found: 446.1804;

- **7b**: *N*-[(*Z*/*E*)-*p*-tolylmethyleneamino]-2-(3,5,7-trimethyl-4-oxo-6-phenyl-pyrrolo[3,4-*d*]pyridazin-1-yl)oxy-acetamide

Yield: 77.76% m.p.: 271–272 °C

FT-IR (selected lines, γmax, cm^−1^): 3205 (*N*H), 3160, 3057 (C-H arom.), 2978, 2915 (C-H aliph.), 1697, 1615 (C=O), 1548 (C=N); ^1^H NMR (300 MHz, DMSO-*d*_6_) δ: 2.27 (s, 3H, 7-CH_3_), 2.32 (s, 6H, 5-CH_3_, Ar-CH_3_), 3.38 (s, 3H, 3-CH_3_), 4.78 (*trans*) and 5.26 (*cis*) (2s, 2H, -*O*-CH_2_-); 7.22–7.25 (m, 2H, Ar-H), 7.38–7.41 (m, 2H, Ar-H), 7.55–7.61 (m, 5H, Ar-H), 7.95 (*trans*) and 8.18 (*cis*) (2s, 1H, *N*=CH), 11.48 (*trans*) and 11.52 (*cis*) (2s, 1H, *N*H); ^13^C NMR (75 MHz, DMSO-*d*_6_) δ: 11.54, 12.14, 21.47, 63.22, 109.18, 111.86, 124.75, 127.27, 128.33, 129.88, 130.28, 132.04, 136.90, 140.31, 144.44, 147.60, 148.82, 158.79, 169.00; MS (ESI-MS) (*m*/*z*): calcd. for C_25_H_25_N_5_O_3_ [L+H]^+^: 444.2030; found: 444.2059;

- **5c:** *N*-[(*Z*/*E*)-benzylideneamino]-2-[6-(3-chlorophenyl)-3,5,7-trimethyl-4-oxo-pyrrolo[3,4-*d*]pyridazin-1-yl]oxyacetamide

Yield: 73.49%; m.p.: 273–275 °C;

FT-IR (selected lines, γ_max_, cm^−1^): 3214 (*N*H), 3138 (C-H arom.), 2977, 2912 (C-H aliph.), 2856 (C-H azomethine), 1712, 1620 (C=O), 1550 (C=N); ^1^H NMR (300 MHz, DMSO-*d*_6_) δ: 2.30 (s, 3H, 7-CH_3_), 2.34 (s, 3H, 5-CH_3_), 3.38 (s, 3H, 3-CH_3_), 4.81 (*trans*) and 5.29 (*cis*) (2s, 2H, -*O*-CH_2_-); 7.42–7.66 (m, 9H, Ar-H), 8.00 (*trans*) and 8.23 (*cis*) (2s, 1H, *N*=CH), 11.56 (*trans*) and 11.61 (*cis*) (2s, 1H, *N*H); ^13^C NMR (75 MHz, DMSO-*d*_6_) δ: 11.46, 12.06, 37.24, 63.08, 108.85, 111.80, 124.63, 127.27, 127.42, 128.45, 129.26, 130.11, 130.37, 131.81, 134.41, 137.89, 144.14, 148.66, 158.41, 168.98; HR-MS (ESI-MS) (*m*/*z*): calcd. for C_24_H_22_ClN_5_O_3_ [L+H]^+^: 464.1484; found: 464.1465;

- **6c:** 2-[6-(3-chlorophenyl)-3,5,7-trimethyl-4-oxo-pyrrolo[3,4-*d*]pyridazin-1-yl]oxy-*N*-[(*Z*/*E*)-(2-hydroxyphenyl)methyleneamino]acetamide

Yield: 68.91%; m.p.: 230–232 °C;

FT-IR (selected lines, γ_max_, cm^−1^): 3225 (*N*H), 3090 (*O*H), 2917 (C-H aliph.), 2862 (C-H azomethine), 1698, 1616 (C=O), 1550 (C=N); ^1^H NMR (300 MHz, DMSO-*d*_6_) δ: 2.29 (s, 3H, 7-CH_3_), 2.34 (s, 3H, 5-CH_3_), 3.37 (s, 3H, 3-CH_3_), 4.82 (*trans*) and 5.25 (*cis*) (2s, 2H, -*O*-CH_2_-); 6.88–6.91 (m, 2H, Ar-H), 7.23 (m, 1H, Ar-H), 7.42–7.51 (m, 1H, Ar-H), 7.65 (m, 4H, Ar-H), 8.30 (*trans*) and 8.47 (*cis*) (2s, 1H, *N*=CH), 10.03 (*trans*) and 11.00 (*cis*) (2s, 1H, Ar-*O*H), 11.49 (*trans*) and 11.83 (*cis*) (2s, 1H, *N*H); ^13^C NMR (75 MHz, DMSO-*d*_6_) δ: 11.47, 12.06, 37.24, 111.80, 116.81, 119.10, 119.87, 120.48, 124.65, 124.78, 127.42, 128.46, 130.12, 130.20, 130.32, 131.83, 134.42, 137.90, 141.72, 147.92, 148.52, 148.67, 156.84, 157.74, 158.41, 164.27, 168.65; HR-MS (ESI-MS) (*m*/*z*): calcd. for C_24_H_22_ClN_5_O_4_ [L+H]^+^: 480.1433; found: 480.1415;

- **7c:** 2-[6-(3-chlorophenyl)-3,5,7-trimethyl-4-oxo-pyrrolo[3,4-*d*]pyridazin-1-yl]oxy-*N*-[(*Z*/*E*)-*p*-tolylmethyleneamino]acetamide

Yield: 72.77%; m.p.: 248–250 °C;

FT-IR (selected lines, γ_max_, cm^−1^): 3207 (*N*H), 3098 (C-H arom.), 2938, 2912 (C-H aliph.), 2856 (C-H azomethine), 1701, 1617 (C=O), 1547 (C=N); ^1^H NMR (300 MHz, DMSO-*d*_6_) δ: 2.29 (s, 3H, 7-CH_3_), 2.32 (s, 3H, Ar-CH_3_), 2.34 (s, 3H, 5-CH_3_), 3.37 (s, 3H, 3-CH_3_), 4.79 (*trans*) and 5.27 (*cis*) (2s, 2H, -*O*-CH_2_-); 7.22–7.25 (m, 2H, Ar-H), 7.40–7.42 (m, 1H, Ar-H), 7.54–7.66 (m, 5H, Ar-H), 7.96 (*trans*) and 8.19 (*cis*) (2s, 1H, *N*=CH), 11.48 (*trans*) and 11.54 (*cis*) (2s, 1H, *N*H); ^13^C NMR (75 MHz, DMSO-*d*_6_) δ:11.47, 12.05, 21.46, 37.23, 63.10, 108.87, 111.80, 124.63, 127.25, 127.42, 127.52, 128.45, 129.86, 130.08, 130.17, 131.73, 131.81, 134.41, 137.90, 140.16, 144.22, 148.68, 158.41, 164.21, 168.87; HR-MS (ESI-MS) (*m*/*z*): calcd. for C_25_H_24_ClN_5_O_3_ [L+H]^+^: 478.1640; found: 478.1642;

### 4.2. Biological Evaluation

#### 4.2.1. Cell Line and Culture Conditions

Normal human dermal fibroblasts (NHDF) were purchased from the American Type Culture Collection (ATCC) for cytotoxicity evaluation of the tested compounds. The cells were grown in Dulbecco’s Modified Eagle Medium DMEM with 4.5 g/mL glucose without phenol red supplemented with 10% FBS and 2 mM *L*-glutamine, and 25 μg/mL gentamicin. The NHDF cells were cultured under standard conditions (5% CO_2_, 37 °C, and 95% humidity) with evaluation morphology and confluence at least twice a week. For the assay, the cells were used only in a logarithmic growth phase, and the TrypLE solution was used for 2 min at 37 °C to detach cells. Then, the TrypLE solution was inactivated by adding a culture medium, and the suspension of cells was collected into a tube and centrifuged for 5 min at 1000 rpm. Then, the supernatant was removed, fresh medium was added, and the pellet was resuspended and calculated using the Bürcker chamber. The suspension of cells was prepared to allow seeding 10,000 cells/well density and incubated for 24 h to regenerate in CO_2_-incubator.

#### 4.2.2. Tested Compounds

The tested compounds were dissolved in DMSO to a final concentration of 10 mM and stored at −20 °C. Before use, the compound solution was kept at room temperature until thawing, and then, samples with 3 different concentrations of compounds were prepared (100 µM, 50 µM, and 10 µM) for each compound. The tested concentrations of compounds were prepared in a culture medium. At the highest concentration of each compound, the DMSO content did not exceed 1%.

#### 4.2.3. Cytotoxicity Assay

The evaluation of the cytotoxicity of the newly synthesized compounds was carried out in accordance with the ISO 10,993 part V standard by performing the MTT test. After seeding at a density of 10,000 cells per well and adhering overnight, the NHDF cells were treated with the test compounds for 24 h in a CO_2_ incubator. The supernatant was then removed, the cells were washed with PBS, a 1mg/mL solution of MTT dissolved in MEM was added, and the cultures were re-incubated for 2 h in a CO_2_ incubator. The medium was then removed, and for a further 30 min, the formazan crystals were dissolved in 100 µL of isopropanol. The absorbance was measured with a Varioskan Go microplate reader at a wavelength of 570 nm.

#### 4.2.4. Evaluation of COX-1, COX-2 and 15-LOX Inhibitory Activity

Cayman’s COX Colorimetric Inhibitor Screening Assay Kit (Item No. 701050) and Cayman’s Lipoxygenase Inhibitor Screening Assay Kit (Item No. 760700) (Cayman Chemical, 1180 East Ellsworth Road, Ann Arbor, MI 48108, USA) were used to evaluate the inhibitory activity of investigated compounds. For the evaluation of COX-1 and COX-2 inhibitory activity, each sample was prepared in triplicate at a concentration of 100 µM. To assess anti-COX-1 and anti-COX-2 activity, the assay was started with a 2 min incubation at room temperature. Then, the peroxidase activity was measured with a Varioskan Go microplate reader at a wavelength of 590 nm. In contrast, in evaluating anti-15-LOX activity, each sample was prepared in duplicate at a concentration of 100 µM. For the evaluation of 15-LOX inhibitory activity, the assay was started with a 5 min incubation at room temperature, and then activity was measured with a Varioskan Go microplate reader at 490 nm. The test detected and measured hydroperoxides produced in the lipoxygenation reaction using purified LOX.

The results are presented as IC_50_ values, i.e., the concentration at which 50% inhibition of enzyme activity occurred for COX-1, COX-2, and 15-LOX, respectively. The IC_50_ values were calculated according to the instructions of the manufacturer’s kits. In the case of the cyclooxygenase inhibitory activity assessment, meloxicam, diclofenac, and celecoxib were used as references. For the evaluation of lipoxygenase inhibitory activity, zileuton was used as a reference compound. All further details concerning the conducted experiments are described in the instruction provided by the supplier.

#### 4.2.5. Statistical Analysis

The IC_50_ values (concentration that inhibits 50% of cell viability) for the cytotoxicity results were estimated by non-linear regression using the relationship of the biological effect to the molar concentrations of the compounds (four-parameter Hill logistic model). For enzyme assays, the IC_50_ was calculated according to the procedure provided by the kit manufacturer. The significance was set at *p* < 0.05. Based on the tests performed, the test power was calculated to be greater than 80%.

### 4.3. Molecular Docking Studies

The structure of the studied compounds was optimized using DFT functional with B3LYP/6-31+G (d.p) basic set [82,83,84]. Calculations were carried out using the Gaussian 2016 C.01 software package [85]. The crystal structures of human COX-1 (PBD:4O1Z), COX-2 (PBD: 4M11), 15-LOX (PDB:4NRE), has (2XW0), and AAG (PDB:3KQ0) were obtained from the Protein Data Bank (http://www.rcsb.org, accessed on 14 February 2023). All the ligands and water molecules (except the iron ion with the coordinated two waters in the 15-LOX structure) were removed, and then polar hydrogen atoms and Kollman charges were added to the protein structure using AutoDock Tools 1.5.6 [86]. To prepare the ligand molecules, partial charges were calculated, nonpolar hydrogens were merged, and rotatable bonds were assigned. The molecular docking study was conducted using AutoDockVina 1.1.2 [87]. Exhaustiveness values were set as 8, 16, 24, and 60. The center of the grid box was set according to crystallized ligands into the binding pocket site in the crystal structure. The docking protocol was first validated by self-docking of the crystallized ligands. The interactions between the studied compounds and COX-1, COX-2, 15-LOX, HSA, and AAG were further analyzed using Discovery Studio Visualizer v.20 (https://www.3ds.com/, accessed on 14 February 2023). The visualizations were conducted using the ChimeraX 1.4 software [88].

### 4.4. Spectroscopic Studies

#### 4.4.1. Fluorescence Spectroscopy

The fluorescence study was performed on a Cary Eclipse 500 spectrophotometer (Agilent, Santa Clara, CA, USA). The spectra were recorded in a cuvette with 10 mm path length at an emission range of 300–500 nm, and the excitation wavelength was 280 nm. The measurements were carried out at three temperatures 297, 303, and 308 K. The solutions of plasma proteins: humane serum albumin-HSA (Sigma-Aldrich Chemie GmbH, St. Louis, MO, USA) and human α-1-acid glycoprotein-AAG (Sigma-Aldrich Chemie GmbH, St. Louis, MO, USA) were prepared in phosphate buffer saline (Sigma-Aldrich Chemie GmbH, St. Louis, MO, USA) (pH = 7.4) with a concentration 1 × 10^−6^ M. The final solution concentration of compounds **5a**, **5b**, and **5c** was 1 × 10^−3^ M. The titration of protein solution by studied compounds was conducted with 0.4 increments in prior from 0 to 2 molar ratio.

Binding site studies were conducted using fluorescence measurements. Spectra were recorded on Cary Eclipse 500 spectrophotometer (Agilent, Santa Clara, CA, USA) at room temperature with a 10 mm path length. HSA and AAG solutions were prepared in phosphate buffer, giving concentrations equal to 1 × 10^−6^ M. The concentrations of dansylated-L-glycine (DanG), dansylated-L-phenylalanine (DanF), and quinaldine red (QR) were 1 × 10^−3^ M. The densylated amino acids and QR solutions were prepared in ethanol. For the HSA-dansylated amino acids studies, the excitation wavelength was equal to 350 nm, while for the AAG-QR system, it was 500 nm, respectively. Then, 3 mL of protein was mixed with 3 µL of marker solution, giving a molar ratio of 1 to 1. Next, successive portions of the analyzed compounds were added to the mixtures prepared in this way, obtaining the appropriate protein-to-compound molar ratios, amounting to 1:0, 1:0.5, 1:1, 1:2, 1:3, 1:4, 1:5, and 1:10 for the HSA-densylated amino acids systems, and 1:0, 1:0.5, 1:1, 1:2, 1:3, 1:4, 1:6, 1:8, and 1:10 for the AAG-QR systems.

#### 4.4.2. Circular Dichroism Spectroscopy

Circular dichroism (CD) spectra were measured on the Jasco J-1500 magnetic circular dichroism spectrometer (JASCO International CO., Tokyo, Japan) at room temperature with a 10 mm path length. All spectra for the HSA and AAG solutions in the absence and presence of 5a, 5b, and 5c were measured under simulated physiological conditions in pH 7.4, in phosphate buffer as a solvent, and were baseline corrected (the spectrum phosphate buffer was used as a baseline). The spectra were measured in the range of 205–250 nm at a scan rate speed of 50 nm/min, with a response time of 1 s. The concentration of HSA and AAG was 1·10^−6^ M, while for **5a**, **5b**, and **5c,** it was 1·10^−3^ M. Experiments were performed to obtain the appropriate protein to compound molar ratios equal to 1:0, 1:0.5, 1:1, 1:1, 1:3, and 1:5. They were respectively added into 3 mL of protein solution successive portions of the solutions of the analyzed compounds. The analysis of the obtained results was performed using the CD Multivariate Calibration Creation and CD Multivariate SSE programs (JASCO International CO., Tokyo, Japan). Protein concentrations were converted here for mean residue molar concentrations.

### 4.5. In Silico Pharmacokinetic, Physicochemical and Drug-Likeness Predictions

The title *N*-acylhydrazone derivatives **5a**-**c**–**7a**-**c** were predicted for their possible pharmacokinetic (ADME), physicochemical, and drug-likeness properties using the SwissADME server (http://www.swissadme.ch) (accessed on 25 May 2023).

## 5. Conclusions

The current manuscript presents the synthesis and preliminary in vitro, computational, and spectroscopic examination of biological activity and pharmacokinetic properties of novel *N*-acylhydrazone derivatives of pyrrolo[3,4-*d*]pyridazinone. Reported compounds revealed no cytotoxic activity in the conducted MTT assay. All studied derivatives appeared to be potent dual COX/LOX inhibitors. Their COX-1 and COX-2 inhibitory activity and COX-2/COX-1 selectivity index were superior to meloxicam. The title compounds’ ability to inhibit 15-LOX was also meaningful and comparable to zileuton. Such simultaneous anti-COX and anti-LOX activity suggests that new pyrrolo[3,4-*d*]pyridazinone derivatives may serve as effective and safe anti-inflammatory drug candidates. Moreover, according to the spectroscopic and molecular docking studies, these compounds form stable complexes with plasma proteins (HSA and AAG) which may impact their distribution and half-life in vivo. Additionally, computational simulations revealed that new molecules are characterized by promising bioavailability scores and good membrane permeability. Obtained results indicate that *N*-acylhydrazone derivatives of pyrrolo[3,4-*d*]pyridazinone are interesting compounds that could play a significant role in the development of new anti-inflammatory agents.

## Figures and Tables

**Table 1:** IC_50_ values were calculated for NHDF cell cultures after 24 h of incubation with the tested compounds **5a-c–7a-c** [mean (± SD); *n* = 5].

Compound	IC_50_ [µM]	Cell Morphology in Culture
**5a**	85.6 ± 2.5	Cells with an elongated shape characteristic of fibroblasts, the granularities were observed in 3–5 fields of view from 10 analyzed fields.
**6a**	88.7 ± 4.1	Cells with an elongated shape characteristic of fibroblasts, the granularities were observed in 3–5 fields of view from 10 analyzed fields.
**7a**	116.4 ± 3.6	Cells with an elongated shape characteristic of fibroblasts, the granularities were observed in 3–5 fields of view from 10 analyzed fields.
**5b**	486.5 ± 6.5	Normal morphology of fibroblasts-elongated cells, single granular cells in 1 of 10 assessed fields of view.
**6b**	80.7 ± 1.9	Cells with an elongated shape characteristic of fibroblasts, the granularities were observed in 3–5 fields of view from 10 analyzed fields.
**7b**	180.7 ± 8.7	Normal morphology of fibroblasts-elongated cells, single granular cells in 1 of 10 assessed fields of view.
**5c**	95.2 ± 1.5	Cells with an elongated shape characteristic of fibroblasts, the granularities were observed in 3–5 fields of view from 10 analyzed fields.
**6c**	210.6 ± 9.6	Normal morphology of fibroblasts–elongated cells, single granular cells in 1 of 10 assessed fields of view.
**7c**	81.6 ± 5.1	Cells with an elongated shape characteristic of fibroblasts, the granularities were observed in 3–5 fields of view from 10 analyzed fields.

**Table 2:** IC_50_ values calculated for COX-1, COX-2, and LOX-15 enzyme [mean (±SD)]; statistical significance of LOX-15 inhibition calculated compared to the control with zileuton (* *p* < 0.05), the statistical significance of COX-1 and COX-2 inhibition calculated compared to the control with meloxicam (**#** *p* < 0.05), celecoxib (^ *p* < 0.05), diclofenac (& *p* < 0.05).

Compound	IC_50_ [µM]			Selectivity Index (SI) ^b^
	15-LOX	COX-1	COX-2	
**5a**	12.9 ± 0.06 *	81.9 ± 2.1 ^# ^ &^	17.2 ± 1.4 ^# ^^	0.21
**6a**	13.6 ± 0.03 *	89.2 ± 4.1 ^# ^ &^	16.4 ± 1.1 ^# ^^	0.18
**7a**	15.3 ± 2.1	93.8 ± 4.2 ^^ &^	16.1 ± 1.8 ^# ^^	0.17
**5b**	13.5 ± 0.02	90.1 ± 1.8 ^# ^ &^	22.7 ± 1.2 ^# ^ &^	0.25
**6b**	13.7 ± 0.03 *	97.7 ± 6.8 ^^ &^	27.7 ± 2.5 ^# ^ &^	0.28
**7b**	13.7 ± 0.07 *	91.5 ± 5.1 ^^ &^	24.5 ± 2.1 ^# ^ &^	0.27
**5c**	12.7 ± 0.04 *	98.9 ± 1.6 ^^ &^	23.6 ± 2.5 ^# ^ &^	0.23
**6c**	14.7 ± 0.02 *	76.8 ± 6.0 ^# ^ &^	9.8 ± 3.1 ^# &^	0.13
**7c**	13.3 ± 0.02	N/A ^a^	22.7 ± 2.5 ^# ^ &^	-
Meloxicam	-	101.4 ± 1.3	60.1 ± 2.4	0.59
Celecoxib	-	53.2 ± 1.9	0.28 ± 2.5	0.005
Diclofenac	-	3.5 ± 2.2	15.4 ± 1.4	4.4
Zileuton	13.41 ± 0.04	-	-	0.21

^a^ N/A stands for “not applicable” ^b^ In vitro COX-2 selectivity index (SI) (COX-2 IC_50_/COX-1 IC_50_).

**Table 3:** The binding affinity (kcal/mol) for the interaction of compounds **5a**-**c–7a**-**c** with 15-LOX, COX-1, and COX-2.

Compound	15-LOX	COX-1	COX-2
**5a**	−8.0	−7.4	−8.7
**6a**	−8.6	−7.6	−9.1
**7a**	−8.4	−5.9	−8.0
**5b**	−10.3	−7.6	−9.3
**6b**	−8.7	−8.8	−10.9
**7b**	−10.2	−5.7	−9.1
**5c**	−9.9	−8.5	−9.6
**6c**	−8.4	−9.4	−10.4
**7c**	−9.8	−6.2	−9.5
Meloxicam	-	−8.8	−8.8
Celecoxib	-	−8.2	−9.5
Diclofenac	-	−7.3	−7.7
Nordihydroguaiaretic Acid (ndga)	−7.1	-	-
C8B	−5.6	-	-
Baicalein	−8.3	-	-

**Table 4:** The Stern–Volmer constants *K_sv_* and the quenching rate constants *k_q_*, binding constants *K_b_*, number of binding sites *n*, and thermodynamic parameters for the interaction of human serum albumin (HSA) with studied compounds at different temperatures.

		Quenching		Binding			Thermodynamic		
	T [K]	*K_sv_* × 10^5^ [dm^3^·mol^−1^]	*k_q_* × 10^13^ [dm^3^·mol^−1^·s^−1^]	*logK_b_*	*K_b_* × 10^5^ [dm^3^·mol^−1^]	n	Δ*G*° [kJ·mol^−1^]	Δ*H*° [kJ·mol^−1^]	Δ*S*° [J·mol^−1^·K^−1^]
**5a**	297 303 308	1.87 ± 0.04 1.71 ± 0.03 1.41 ± 0.03	1.87 1.71 1.41	5.10 ± 0.20 4.95 ± 0.10 4.68 ± 0.09	1.26 0.89 0.48	0.97 ± 0.04 0.95 ± 0.02 0.92 ± 0.02	−29.20	−67.55	−129.11
**5b**	297 303 308	5.55 ± 0.33 5.26 ± 0.28 4.75 ± 0.23	5.55 5.26 4.75	6.58 ± 0.31 6.31 ± 0.56 5.84 ± 0.30	38.02 20.42 6.92	1.15 ± 0.06 1.10 ± 0.10 1.02 ± 0.05	−37.66	−116.19	−264.38
**5c**	297 303 308	4.33± 0.25 3.86 ± 0.13 2.87 ± 0.12	4.33 3.86 2.87	5.21 ± 0.38 5.05 ± 0.14 4.58 ± 0.23	1.62 1.12 0.38	0.92 ± 0.06 0.90 ± 0.02 0.85 ± 0.04	−29.97	−98.113	−229.47

**Table 5:** The Stern–Volmer constants *K_sv_* and the quenching rate constants *k_q_*, binding constants *K_b_*, number of binding sites *n*, and thermodynamic parameters for the interaction of alpha-1-acid glycoprotein (AAG) with studied compounds at different temperatures.

		Quenching		Binding			Thermodynamic		
	T [K]	*K_sv_*·10^4^ [dm^3^·mol^−1^]	*k_q_*·10^12^ [dm^3^·mol^−1^·s^−1^]	*logK_b_*	*K_b_*·10^1^ [dm^3^·mol^−1^]	n	Δ*G*° [kJ·mol^−1^]	Δ*H*° [kJ·mol^−1^]	Δ*S*° [J·mol^−1^·K^−1^]
**5a**	297 303 308	9.04 ± 0.16 3.15 ± 0.41 1.11 ± 0.16	9.04 3.15 1.11	1.82 ± 0.12 1.73 ± 0.27 1.62 ± 0.26	6.61 5.37 4.17	0.44 ± 0.02 0.51 ± 0.05 0.57 ± 0.04	−10.38	−31.59	−71.42
**5b**	297 303 308	10.41 ± 1.08 11.41 ± 1.42 8.39 ± 1.24	10.41 11.41 8.39	2.71 ± 0.21 2.53 ± 0.15 2.35 ± 0.12	51.28 33.88 22.39	0.59 ± 0.04 0.55 ± 0.03 0.54 ± 0.03	−15.49	−57.20	−140.43
**5c**	297 303 308	15.87± 2.41 14.60 ± 2.05 9.99 ± 1.60	15.87 14.60 9.99	2.64 ± 0.16 2.47 ± 0.17 2.31 ± 0.20	43.65 29.51 20.42	0.55 ± 0.03 0.53 ± 0.03 0.52 ± 0.03	−15.04	−53.91	−130.86

**Table 6:** The percentage of the replacement of DanG and DanF from the complex with human serum albumin (HSA) by the analyzed compounds **5a**, **5b**, and **5c**.

HSA:Marker:Analyzed Compound Molar Ratio	DanG			DanF		
	5a	5b	5c	5a	5b	5c
1:1:0	-	-	-	-	-	-
1:1:0.5	0.7%	0%	2.9%	2.3%	5.2%	6.2%
1:1:1	0.7%	0.7%	2.9%	2.7%	9.1%	9.5%
1:1:2	3.7%	5.6%	4.3%	5.9%	15.7%	13.8%
1:1:3	5.2%	7.7%	5.0%	9.0%	20.4%	18.1%
1:1:4	6.7%	9.8%	5.7%	11.7%	23.5%	19.0%
1:1:5	8.5%	11.2%	6.4%	14.9%	27.4%	21.0%
1:1:10	15.7%	14.0%	11.4%	28.8%	32.2%	23.3%

**Table 7:** The percentage of the replacement of QR from the complex with alpha-1-acid glycoprotein (AAG) by the analyzed compounds **5a**, **5b**, and **5c**.

AAG:Marker:Analyzed Compound Molar Ratio	QR		
	5a	5b	5c
1:1:0	-	-	-
1:1:0.5	23.7%	17.2%	31.7%
1:1:1	35.5%	29.1%	39.7%
1:1:2	48.1%	40.3%	52.4%
1:1:3	55.0%	47.0%	54.0%
1:1:4	58.8%	52.2%	54.8%
1:1:6	65.6%	55.2%	55.6%
1:1:8	69.5%	56.7%	55.6%
1:1:10	72.5%	58.2%	55.6%

**Table 8:** The percentage of content of the secondary structure elements in human serum albumin (HSA) in the absence and presence of studied compounds **5a**, **5b**, and **5c** was calculated in the CD Multivariate SSE program.

HSA: Analyzed Compound Molar Ratio	% α-Helix	% β-Sheet	% β-Turn	% Other
**5a**				
1:0	68.1%	1.8%	9.2%	20.8%
1:0.5	67.3%	2.8%	9.2%	20.7%
1:1	66.9%	2.8%	9.3%	20.9%
1:2	66.3%	2.7%	9.4%	21.5%
1:3	66.2%	2.8%	9.5%	21.6%
1:5	66.1%	4.3%	9.3%	20.3%
**Δ = (1:0)–(1:5)**	**−2.0%**			
**5b**				
1:0	68.7%	2.0%	9.2%	20.1%
1:0.5	67.7%	2.2%	9.3%	20.9%
1:1	67.1%	2.5%	9.3%	21.0%
1:2	66.9%	3.2%	9.3%	20.6%
1:3	66.6%	3.7%	9.3%	20.5%
1:5	66.3%	4.3%	9.2%	20.2%
**Δ = (1:0)–(1:5)**	**−2.4%**			
**5c**				
1:0	68.9%	2.1%	9.2%	19.8%
1:0.5	68.4%	2.9%	9.1%	19.6%
1:1	68.0%	2.5%	9.2%	20.3%
1:2	67.8%	3.2%	9.2%	19.8%
1:3	67.8%	3.3%	9.1%	19.7%
1:5	67.3%	3.5%	9.2%	20.0%
**Δ = (1:0)–(1:5)**	**−1.6%**			

**Table 9:** The percentage of content of the secondary structure elements in alpha-1-acid glycoprotein (AAG) in the absence and presence of studied compounds **5a**, **5b**, and **5c** calculated in the CD Multivariate SSE program.

AAG: Analyzed Compound Molar Ratio	% α-Helix	% β-Sheet	% β-Turn	% Other
**5a**				
1:0	20.7%	35.7%	10.8%	32.8%
1:0.5	19.8%	35.6%	10.9%	33.7%
1:1	19.8%	36.4%	10.8%	33.0%
1:2	19.7%	36.8%	10.8%	32.7%
1:3	19.2%	36.5%	10.8%	33.4%
1:5	19.0%	36.4%	10.9%	33.7%
**Δ = (1:0)–(1:5)**	**−1.7%**	**+0.7%**		
**5b**				
1:0	21.2%	35.6%	10.8%	32.4%
1:0.5	20.7%	36.3%	10.7%	32.3%
1:1	20.5%	36.1%	10.7%	32.6%
1:2	20.4%	36.5%	10.7%	32.5%
1:3	20.1%	37.0%	10.6%	32.3%
1:5	19.9%	36.4%	10.8%	32.9%
**Δ = (1:0)–(1:5)**	**−1.3%**	**+0.8%**		
**5c**				
1:0	22.3%	35.6%	10.6%	31.4%
1:0.5	21.5%	36.2%	10.6%	31.7%
1:1	21.2%	36.3%	10.6%	31.9%
1:2	21.0%	37.4%	10.5%	31.2%
1:3	20.9%	37.0%	10.6%	31.6%
1:5	20.4%	36.7%	10.7%	32.3%
**Δ = (1:0)–(1:5)**	**−1.9%**	**+1.1%**		

**Table 10:** The binding affinity [kcal/mol] for interaction of compounds **5a**-**c**–**7a**-**c** with human serum albumin (HSA) and (AAG).

Compound	HSA IIIA (DanF)	AAG
**5a**	−8.2	−8.9
**6a**	−8.5	−8.7
**7a**	−8.5	−8.5
**5b**	−8.9	−9.7
**6b**	−9.0	−9.2
**7b**	−9.1	−9.7
**5c**	−8.9	−9.7
**6c**	−9.0	−9.3
**7c**	−9.3	−9.5

**Table 11:** Calculated physicochemical properties of derivatives **5a**-**c**–**7a**-**c** in relation to Lipinski’s rule of five (Ro5) according to the SwissADME server.

Compound	Physicochemical Properties–Lipinski’s Rule of Five (Ro5)				
	#*H*-Bond Acceptors	#*H*-Bond Donors	Log *P_o/w_* (MLOGP)	MW [g/mol]	#Violations
**5a**	5	1	2.62	409.48	0
**6a**	6	2	2.11	425.48	0
**7a**	5	1	2.84	423.51	0
**5b**	5	1	3.10	429.47	0
**6b**	6	2	2.59	445.47	0
**7b**	5	1	3.31	443.50	0
**5c**	5	1	3.58	463.93	0
**6c**	6	2	3.07	479.92	0
**7c**	5	1	3.78	477.94	0

**Table 12:** The exemplary ADME parameters of investigated derivatives **5a**-**c**–**7a**-**c** calculated by the SwissADME server.

Compound	Pharmacokinetics			
	GI Absorption	BBB Permeability	P-gp Substrate	Water Solubility (ESOL)
**5a**	High	No	No	Moderately soluble
**6a**	High	No	Yes	Soluble
**7a**	High	No	No	Moderately soluble
**5b**	High	No	No	Moderately soluble
**6b**	High	No	No	Moderately soluble
**7b**	High	No	No	Moderately soluble
**5c**	High	No	No	Moderately soluble
**6c**	High	No	No	Moderately soluble
**7c**	High	No	No	Moderately soluble

**Table 13:** Predicted drug-likeness features of the investigated derivatives **5a**-**c**–**7a**-**c** (according to the SwissADME server).

Compound	Drug-Likeness			
	Lipinski	Veber	Bioavailability Score	TPSA [Å^2^]
**5a**	Yes, 0 violation	Yes	0.55	90.51
**6a**	Yes, 0 violation	Yes	0.55	110.74
**7a**	Yes, 0 violation	Yes	0.55	90.51
**5b**	Yes, 0 violation	Yes	0.55	90.51
**6b**	Yes, 0 violation	Yes	0.55	110.74
**7b**	Yes, 0 violation	Yes	0.55	90.51
**5c**	Yes, 0 violation	Yes	0.55	90.51
**6c**	Yes, 0 violation	Yes	0.55	110.74
**7c**	Yes, 0 violation	Yes	0.55	90.51

## Data Availability

All calculations have been carried out in the Wroclaw Centre for Networking and Supercomputing (http://www.wcss.wroc.pl accessed on 14 February 2023) and using the SwissADME server (http://www.swissadme.ch/ accessed on 25 May 2023).

## Supplementary Materials

The following supporting information can be downloaded at: https://www.mdpi.com/article/10.3390/molecules28145479/s1, Table S1—Structures of the new compounds; Table S2—NMR spectra of the new compounds; Table S3—Mass spectra of the new compounds; Table S4—FT-IR spectra of the new compounds; Table S5—Molecular formula strings (CSV) of the reported compounds; Figure S1—Flowchart summarizing Section 4.

## Abbreviations

AA, Arachidonic Acid; ADME, Absorption Distribution Metabolism Excretion; AIA; Aspirin-Induced Asthma, Arg, Arginine; BBB, Blood Brain Barrier, CD, Circular Dichroism; CV, Cardiovascular; COX, Cyclooxygenase; DanF, Dansylated-L-Phenylalanine; DanG, dansylated-L-glycine DMEM, Dulbecco’s Modified Eagle Medium; DMSO, Dimethyl Sulfoxide; ER, Endoplasmic Reticulum; ESI-MS, Electrospray Ionization Mass Spectrometry; FT-IR, Fourier Transform Infrared (Spectroscopy); GI, Gastrointestinal; HMBC, Heteronuclear Multiple Bond Correlation; HSA, Human Serum Albumine; IC, Inhibitory Concentration; Leu, Leucine; LOX, Lipoxygenase; LT, Leukotriene; MPO, Myeloperoxidase; MTT 3-(4,5-dimethylthiazol-2-yl)-2,5-diphenyltetrazolium bromide; MW, Molecular Weight; NA, Not Applicable; NHDF, Normal Human Dermal Fibroblasts; NMR, Nuclear Magnetic Resonance; NSAIDs, Non-Steroidal and Anti-Inflammatory Drugs; PBS, Phosphate Buffered Saline; PG, Prostaglandin; PGE_2_, Prostaglandin E_2_; PUFA, Polyunsaturated Fatty Acid; QR, quinaldine red; Ro5, Rule of Five; RT, Room Temperature; SD, Standard Deviation; Ser, Serine; TLC, Thin Layer Chromatography; TMS, Tetramethylsilane; TNF-α, Tumor Necrosis Factor α; TPSA, Topological Polar Surface Area; Trp, Tryptophan; Tyr, Tyrosine.
