# Supplementary material for: Synthesis, Biological, Spectroscopic and Computational Investigations of Novel N-Acylhydrazone Derivatives of Pyrrolo[3,4-d]pyridazinone as Dual COX/LOX Inhibitors
Source: Molecules. 2023 Jul 18;28(14):5479. doi: 10.3390/molecules28145479 (PMC10383271; doi:10.3390/molecules28145479)

molecules

MDPI

Supplementary materials

Table of Contents

Table S1 - Structures of new compounds 1

Table S2 - NMR spectra of new compounds 2

Table S3 - Mass spectra of new compounds 6

Table S4 – FT-IR spectra of new compounds 12

Table S5 – Molecular formula strings (CSV) of reported compounds 17

Figure S1 - Flowchart summarizing the Materials and Methods section 18

Table S1 - Structures of new compounds

**2c**	**3c**	**4c**
**5a**	**6a**	**7a**
**5b**	**6b**	**7b**

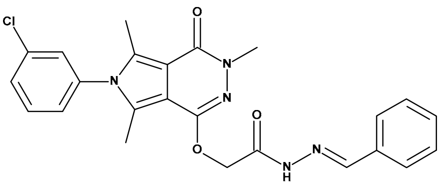	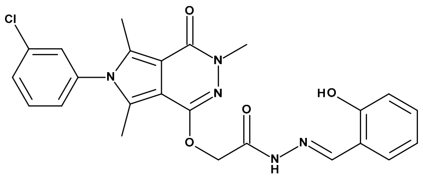	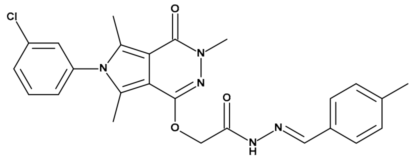
**5c**	**6c**	**7c**

Table S2 - NMR spectra of new compounds

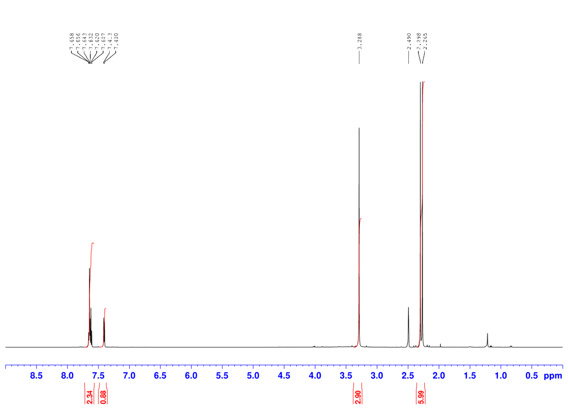

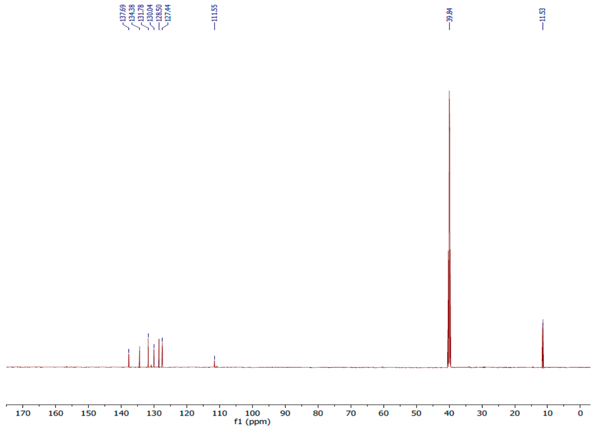

2c

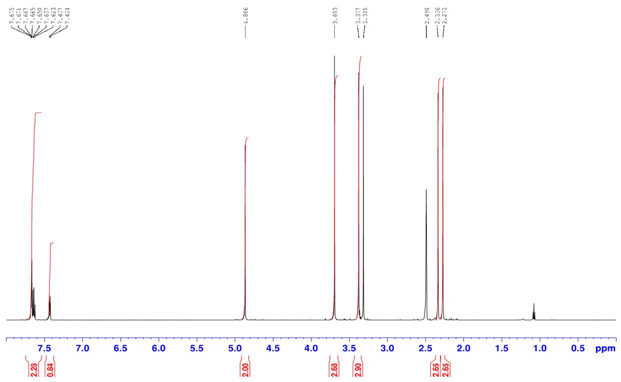

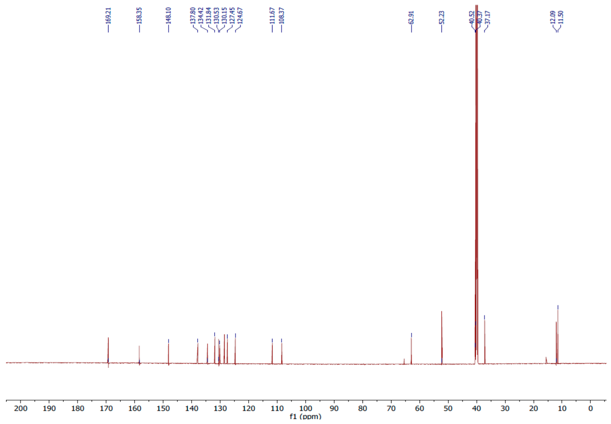

3c

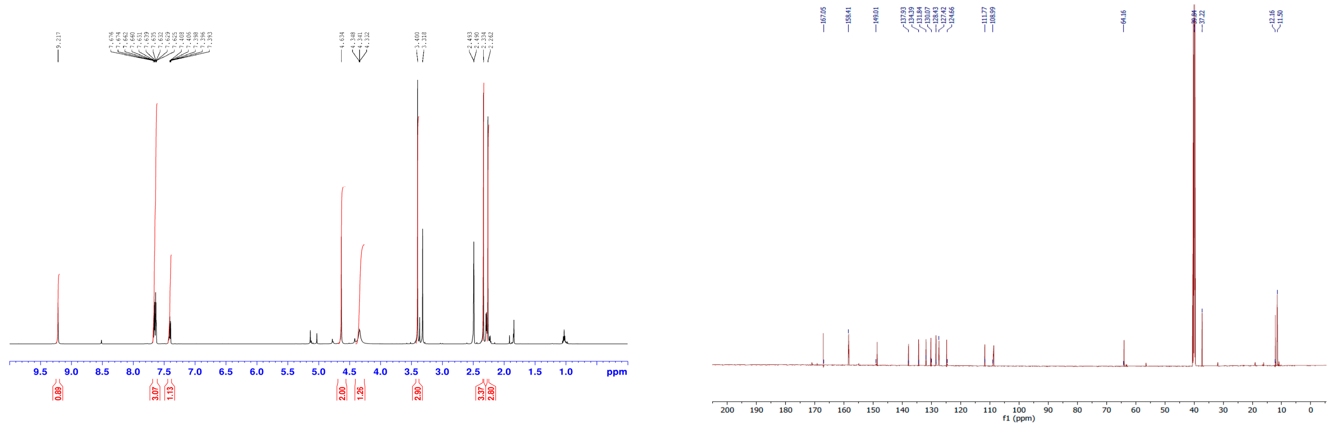

4c

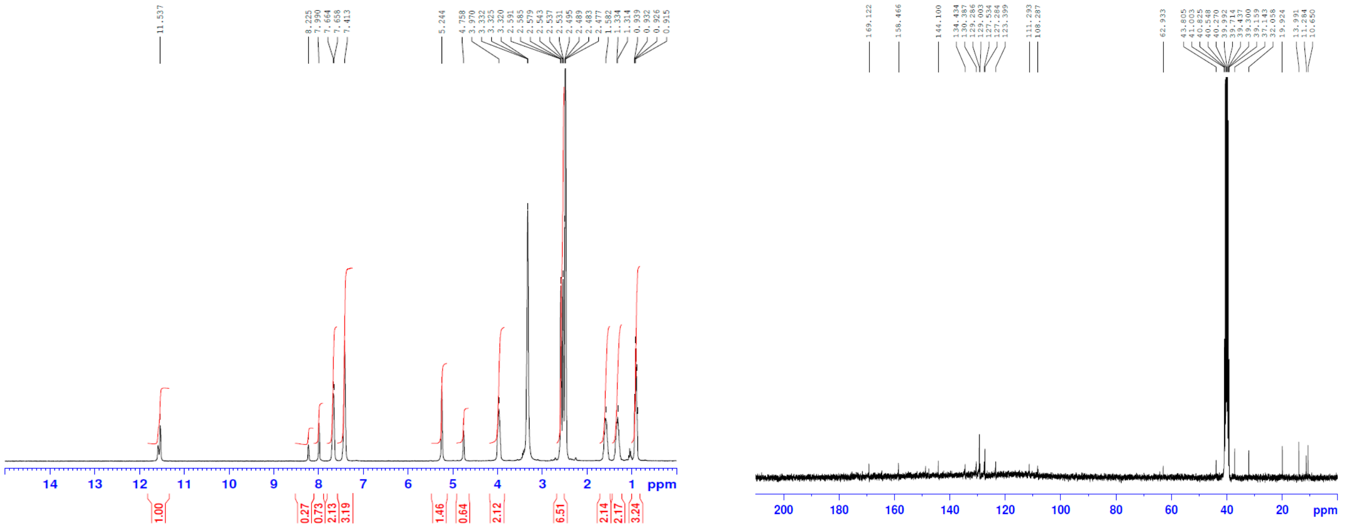

5a

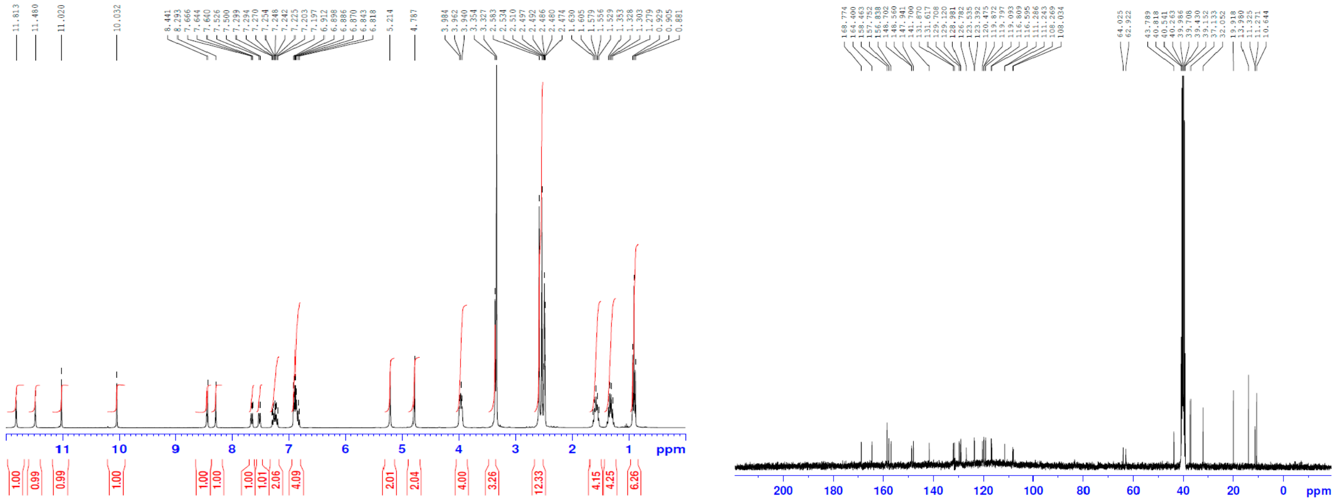

6a

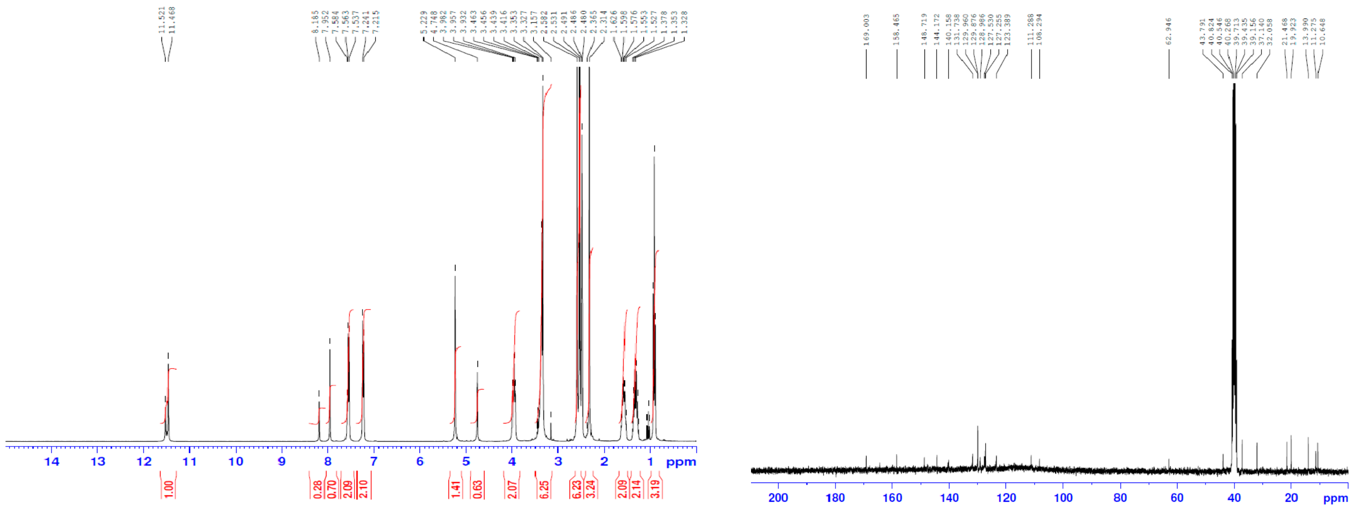

7a

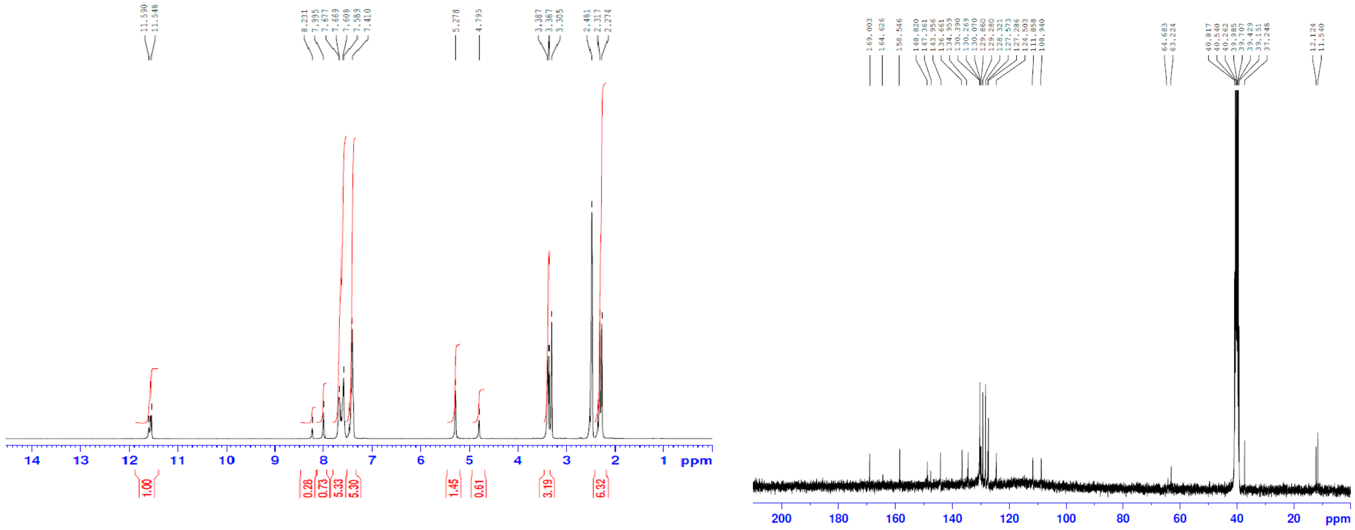

5b

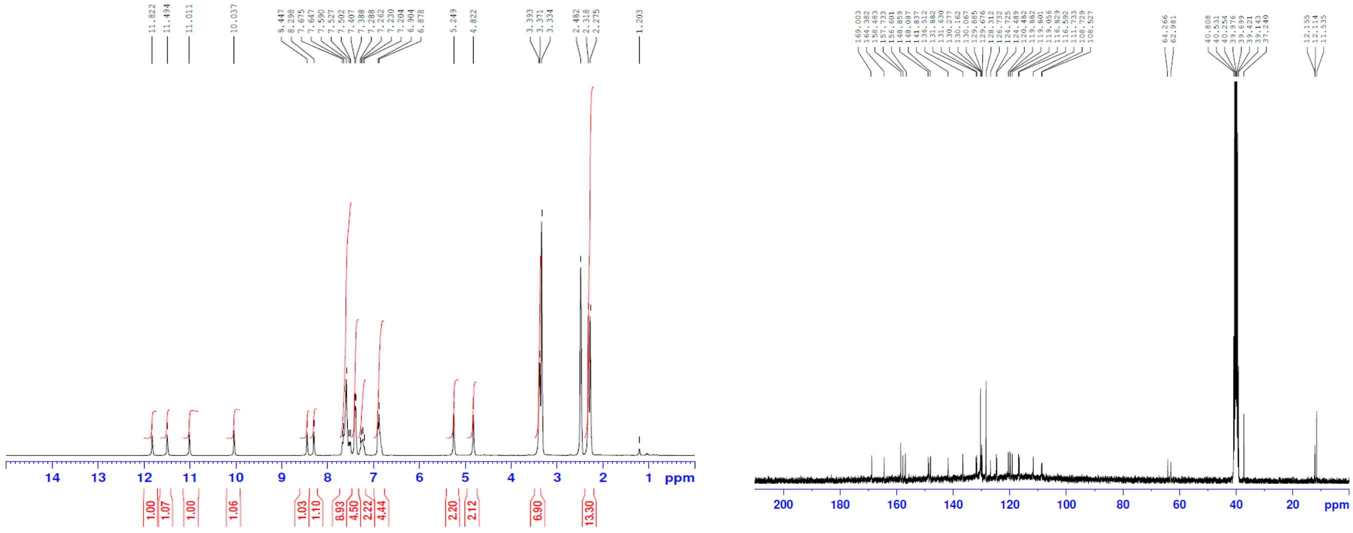

6b

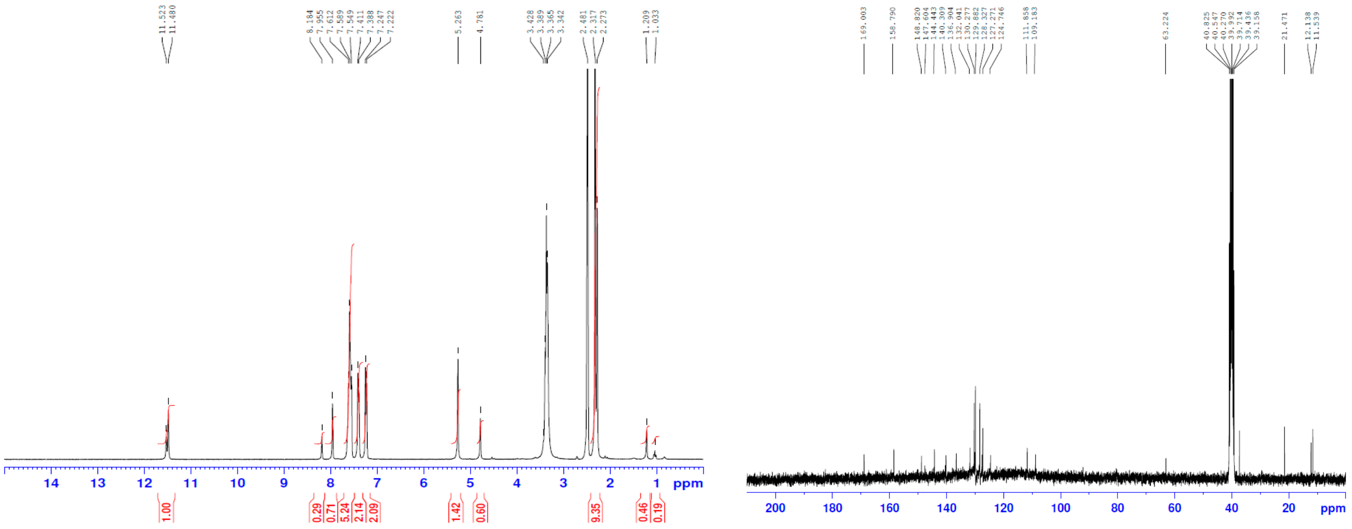

7b

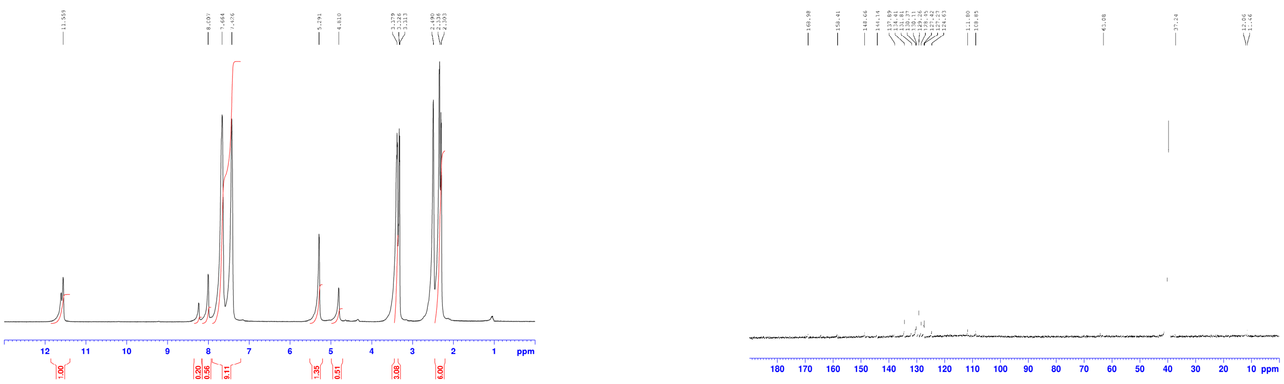

5c

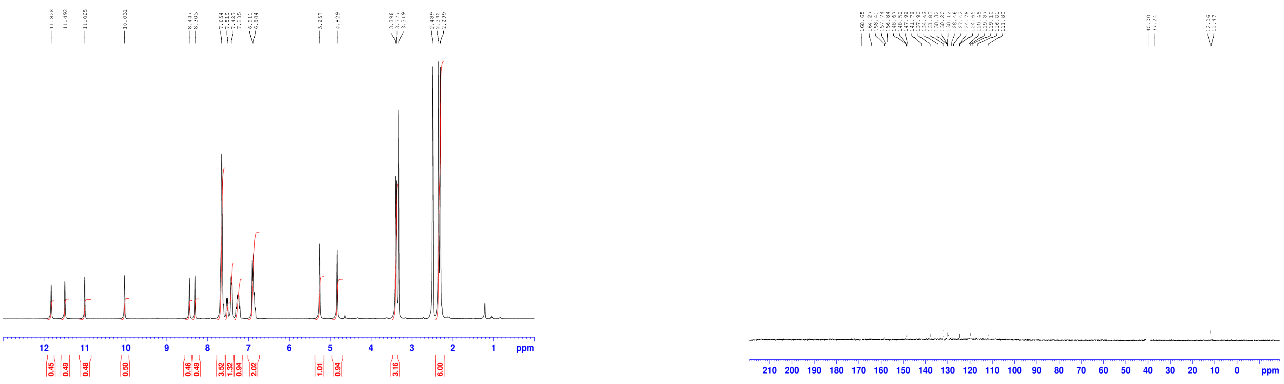

6c

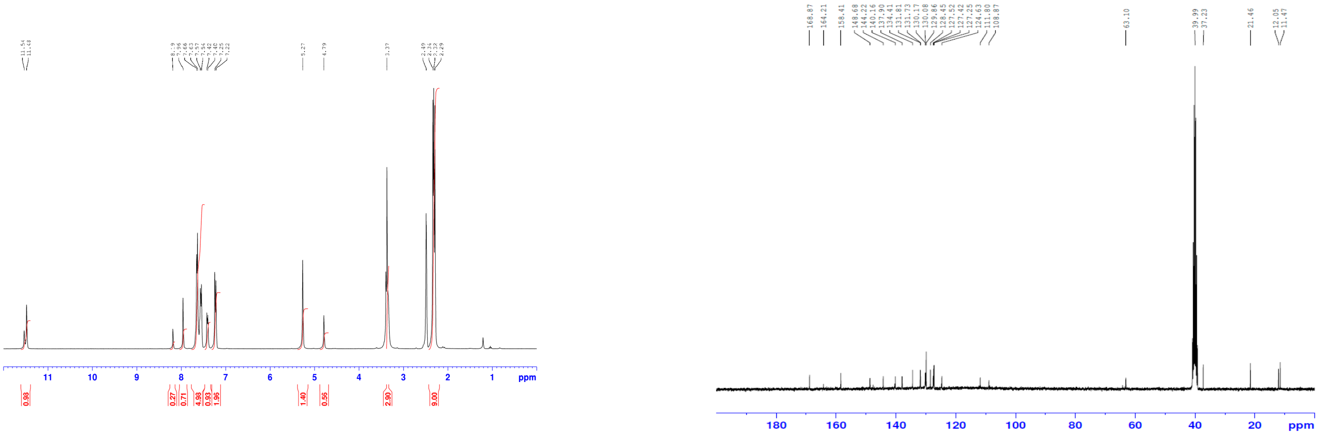

7c

Table S3 - Mass spectra of new compounds

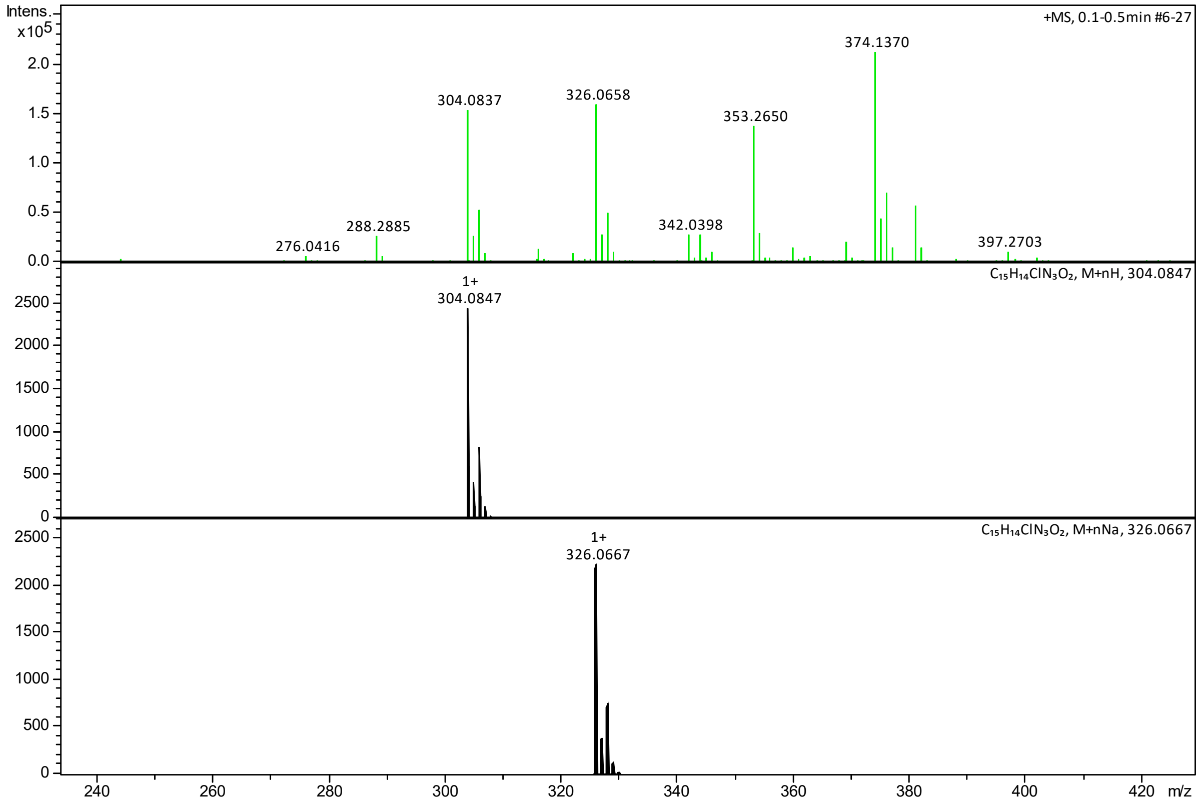

2c

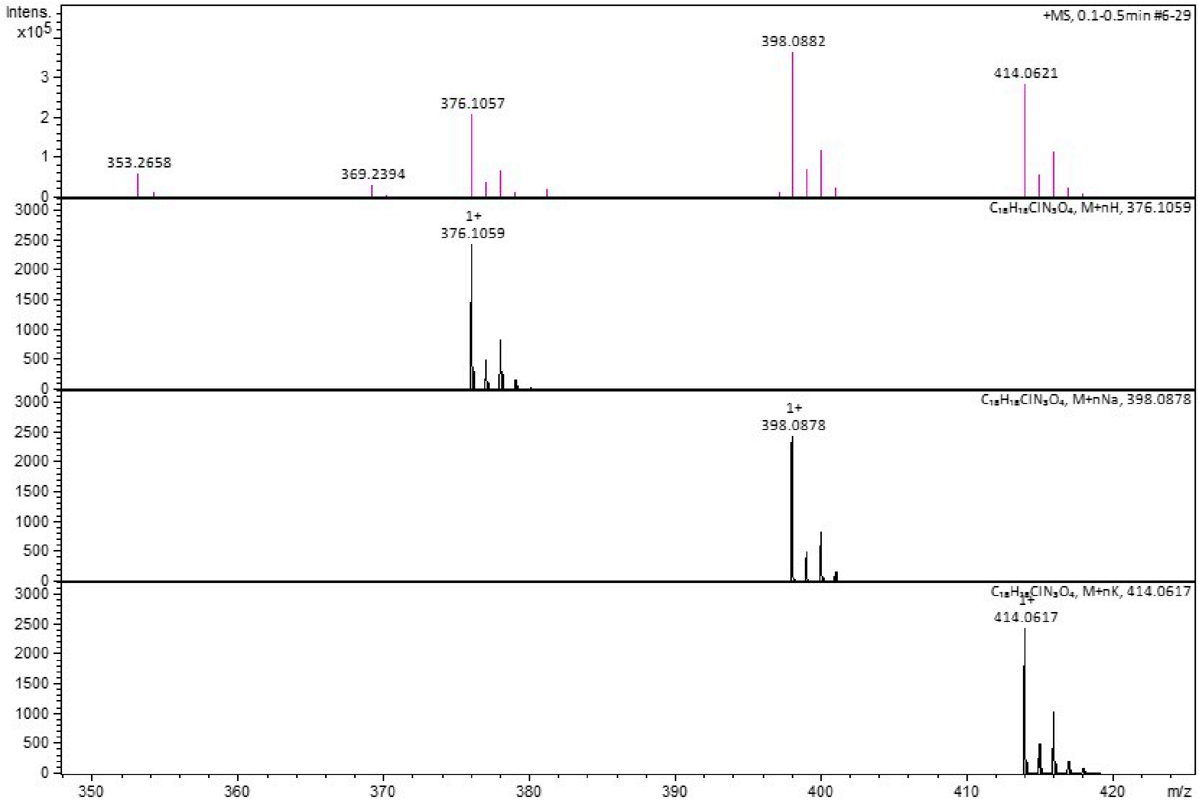
Intens.
x10^5
+MS, 0.1-0.5min #6-29
353.2658
369.2394
376.1057
398.0882
414.0621
C18H18ClN3O4, M+nH, 376.1059
1+
376.1059
C18H18ClN3O4, M+nNa, 398.0878
1+
398.0878
C18H18ClN3O4, M+nK, 414.0617
1+
414.0617
m/z

3c

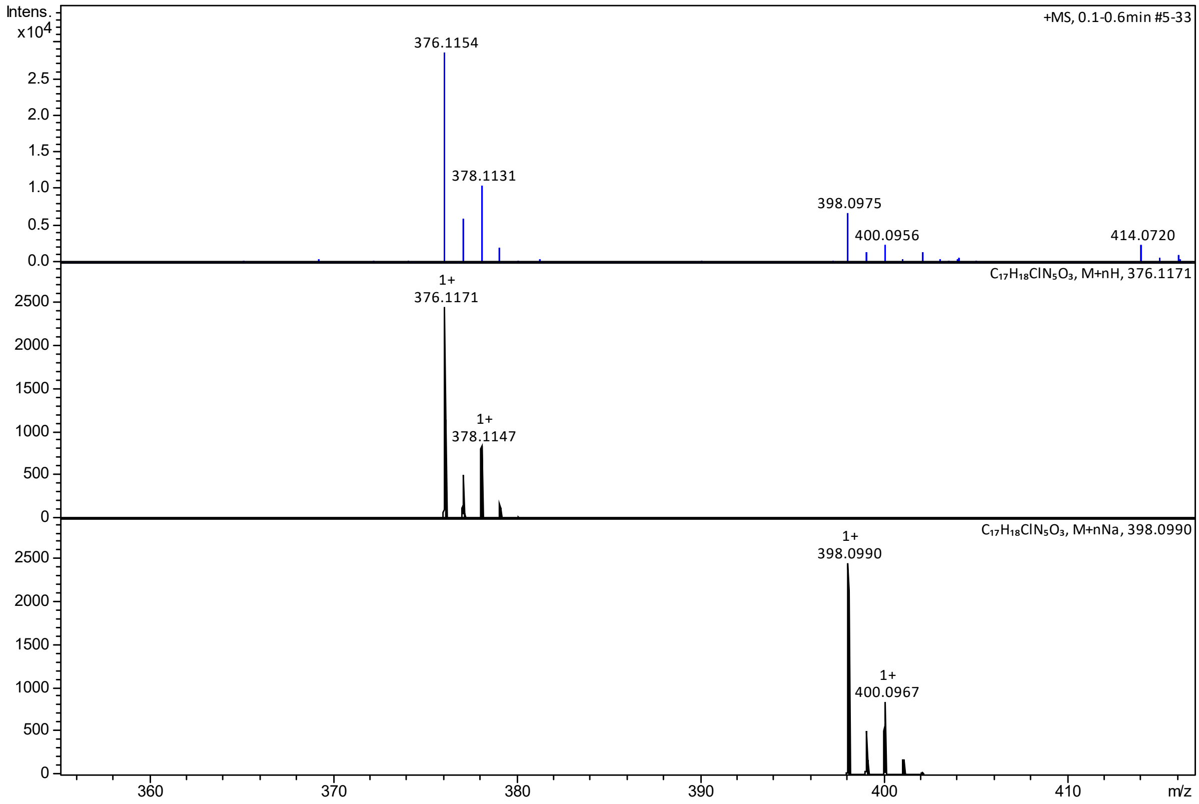
Intens.
x10^4
+MS, 0.1-0.6min #5-33
376.1154
378.1131
398.0975
400.0956
414.0720
C17H18ClN5O3, M+nH, 376.1171
1+
376.1171
1+
378.1147
C17H18ClN5O3, M+nNa, 398.0990
1+
398.0990
1+
400.0967
m/z

4c

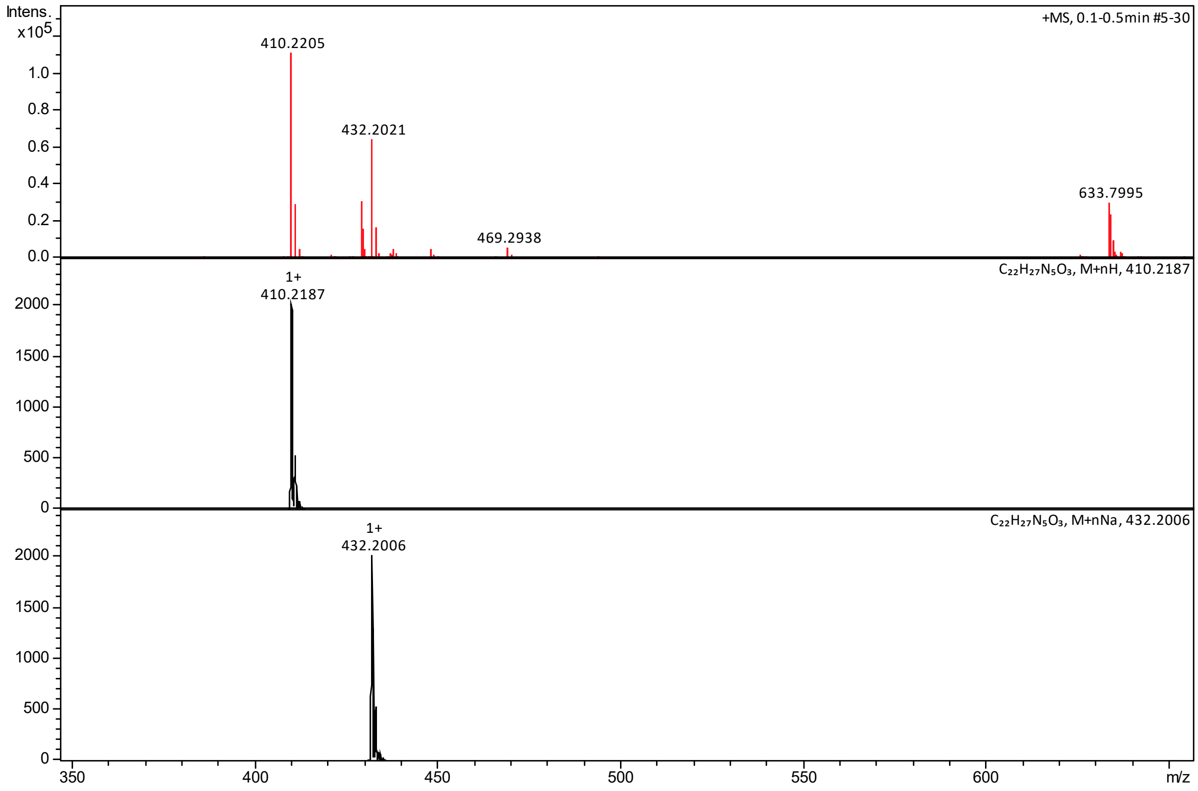
Intens.
x10^5
+MS, 0.1-0.5min #5-30
410.2205
432.2021
469.2938
633.7995
1+
410.2187
C22H27N5O3, M+nH, 410.2187
1+
432.2006
C22H27N5O3, M+nNa, 432.2006
m/z

5a

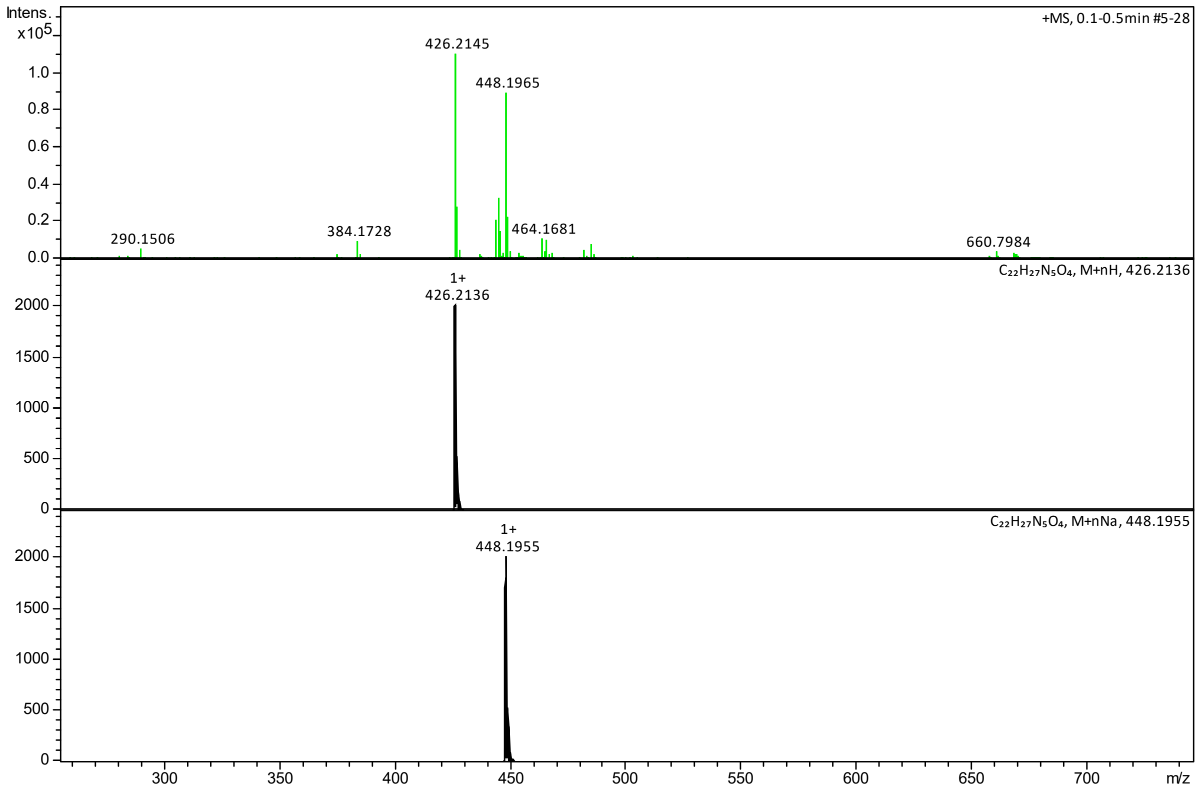
Intens.
x10^5
+MS, 0.1-0.5min #5-28
426.2145
448.1965
290.1506
384.1728
464.1681
660.7984
1+
426.2136
C22H27N5O4, M+nH, 426.2136
1+
448.1955
C22H27N5O4, M+nNa, 448.1955
m/z

6a

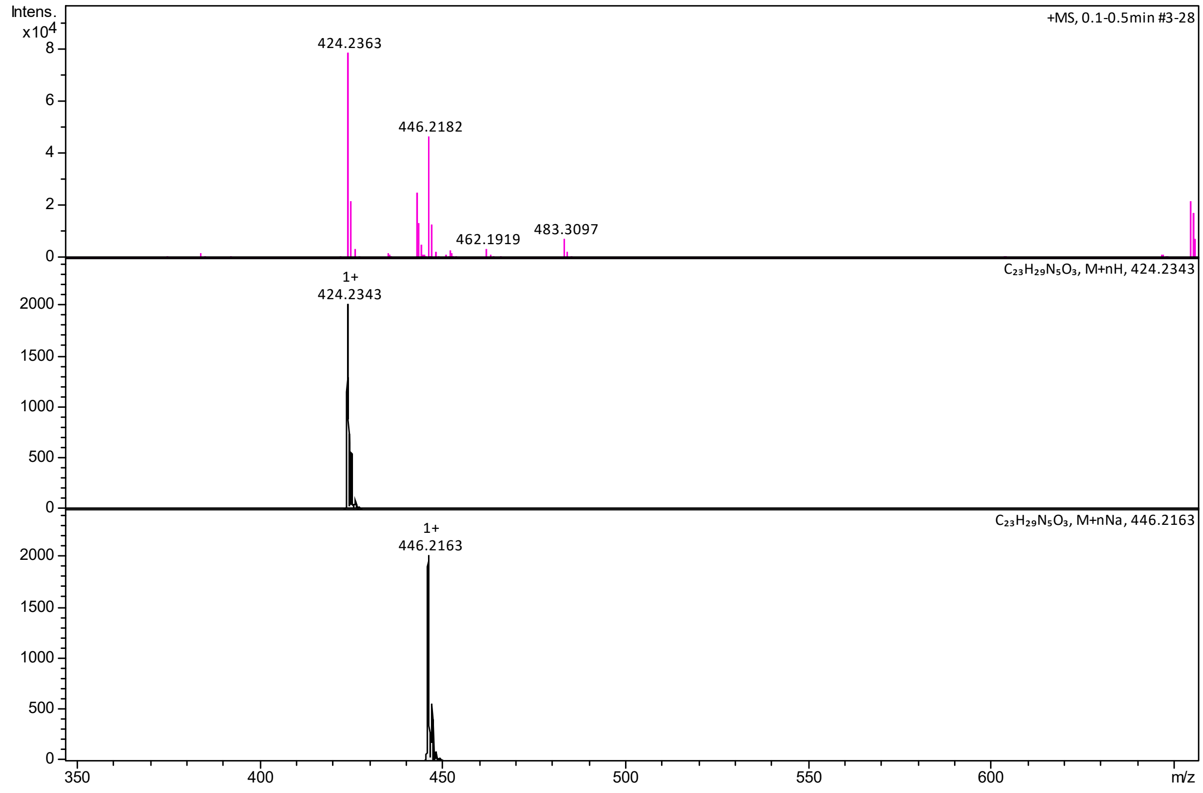

Intens.
x10^4
+MS, 0.1-0.5min #3-28
424.2363
446.2182
462.1919
483.3097
C₂₃H₂₉N₅O₃, M+nH, 424.2343
1+
424.2343
C₂₃H₂₉N₅O₃, M+nNa, 446.2163
1+
446.2163
m/z

7a

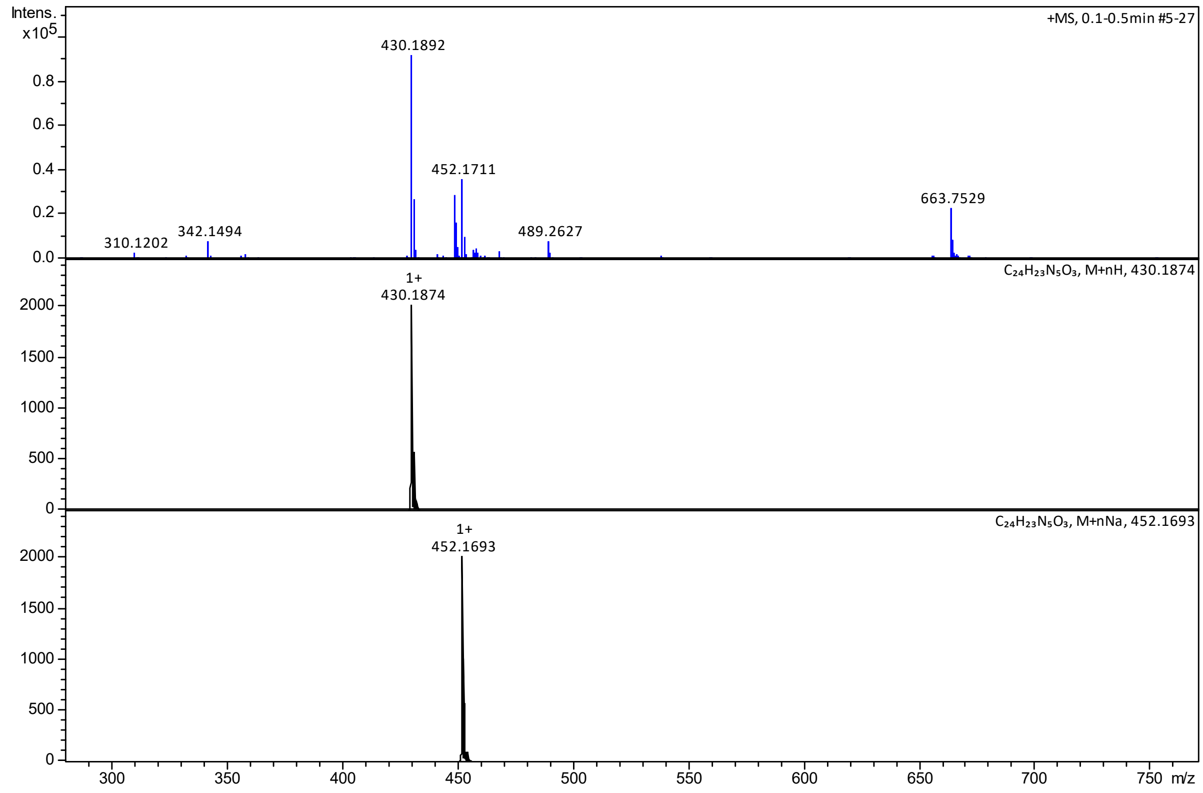

Intens.
x10^5
+MS, 0.1-0.5min #5-27
430.1892
452.1711
663.7529
342.1494
310.1202
489.2627
C₂₄H₂₃N₅O₃, M+nH, 430.1874
1+
430.1874
C₂₄H₂₃N₅O₃, M+nNa, 452.1693
1+
452.1693
m/z

5b

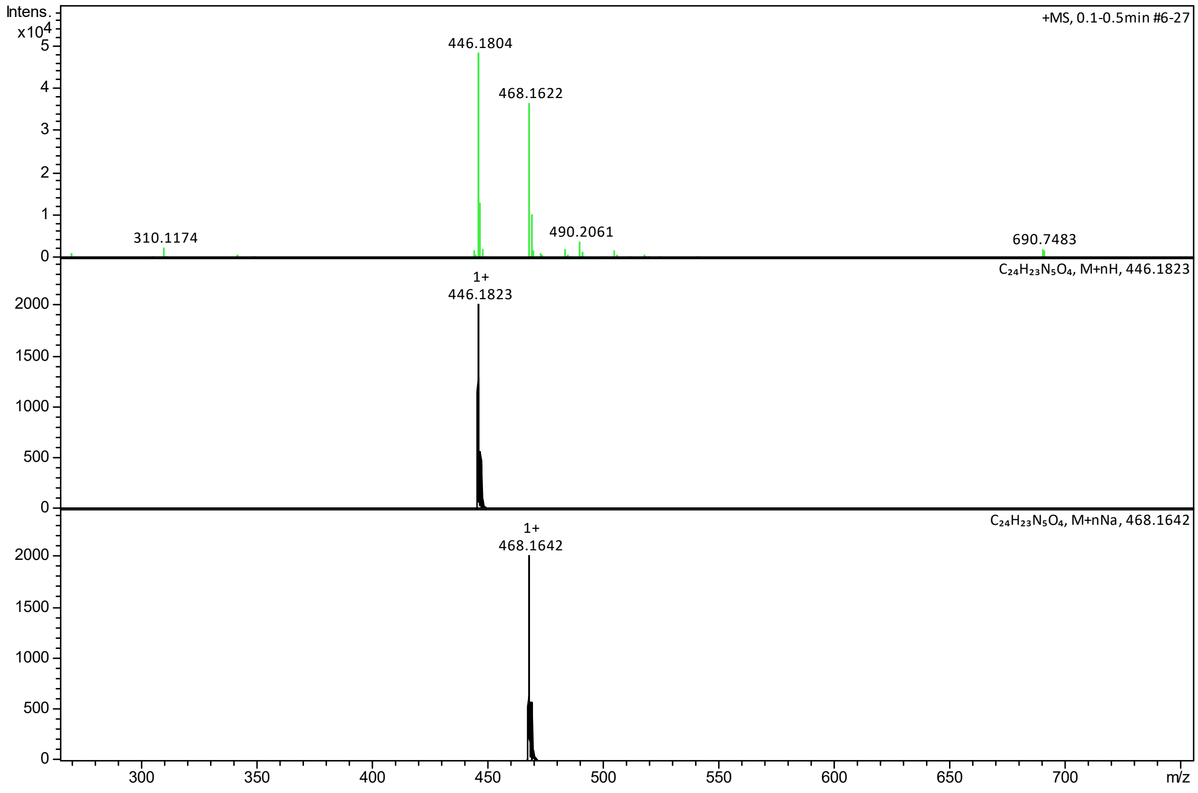
Intens.
x10^4
+MS, 0.1-0.5min #6-27
446.1804
468.1622
310.1174
490.2061
690.7483
C₂₄H₂₃N₅O₄, M+nH, 446.1823
1+
446.1823
C₂₄H₂₃N₅O₄, M+nNa, 468.1642
1+
468.1642
m/z

6b

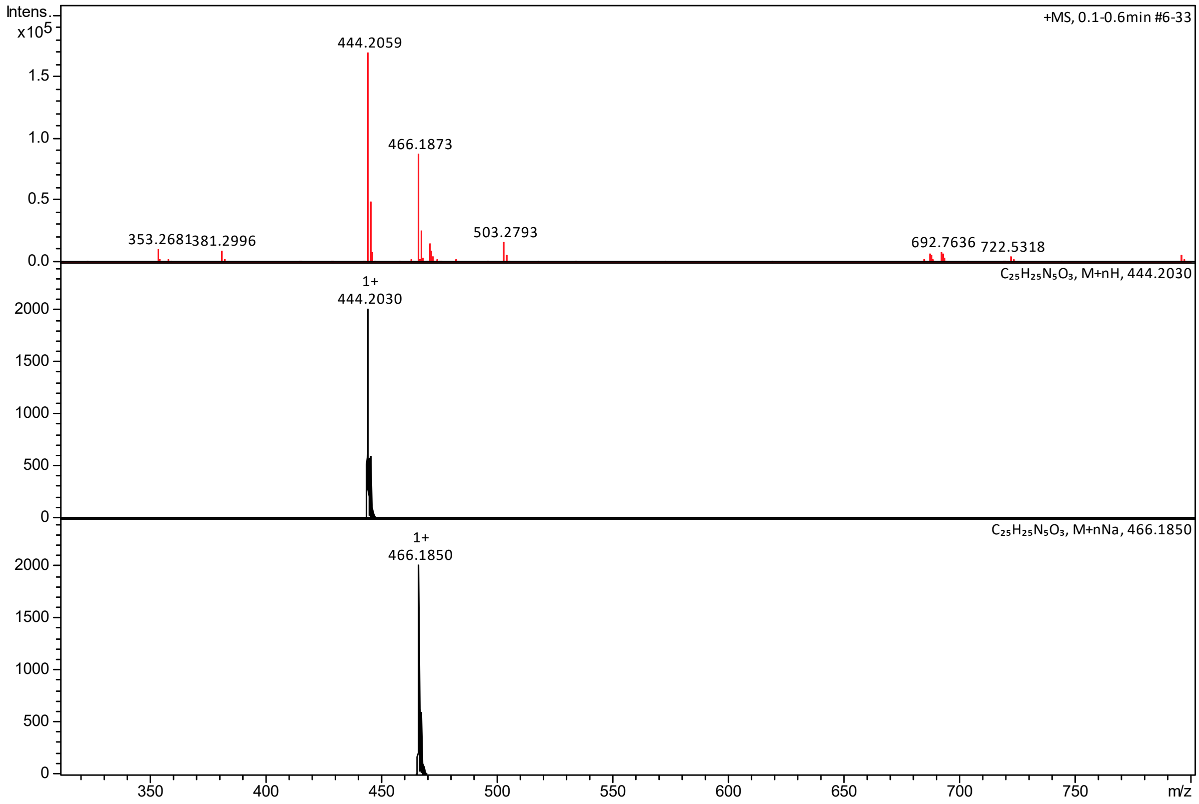
Intens.
x10^5
+MS, 0.1-0.6min #6-33
444.2059
466.1873
353.2681
381.2996
503.2793
692.7636
722.5318
C₂₅H₂₅N₅O₃, M+nH, 444.2030
1+
444.2030
C₂₅H₂₅N₅O₃, M+nNa, 466.1850
1+
466.1850
m/z

7b

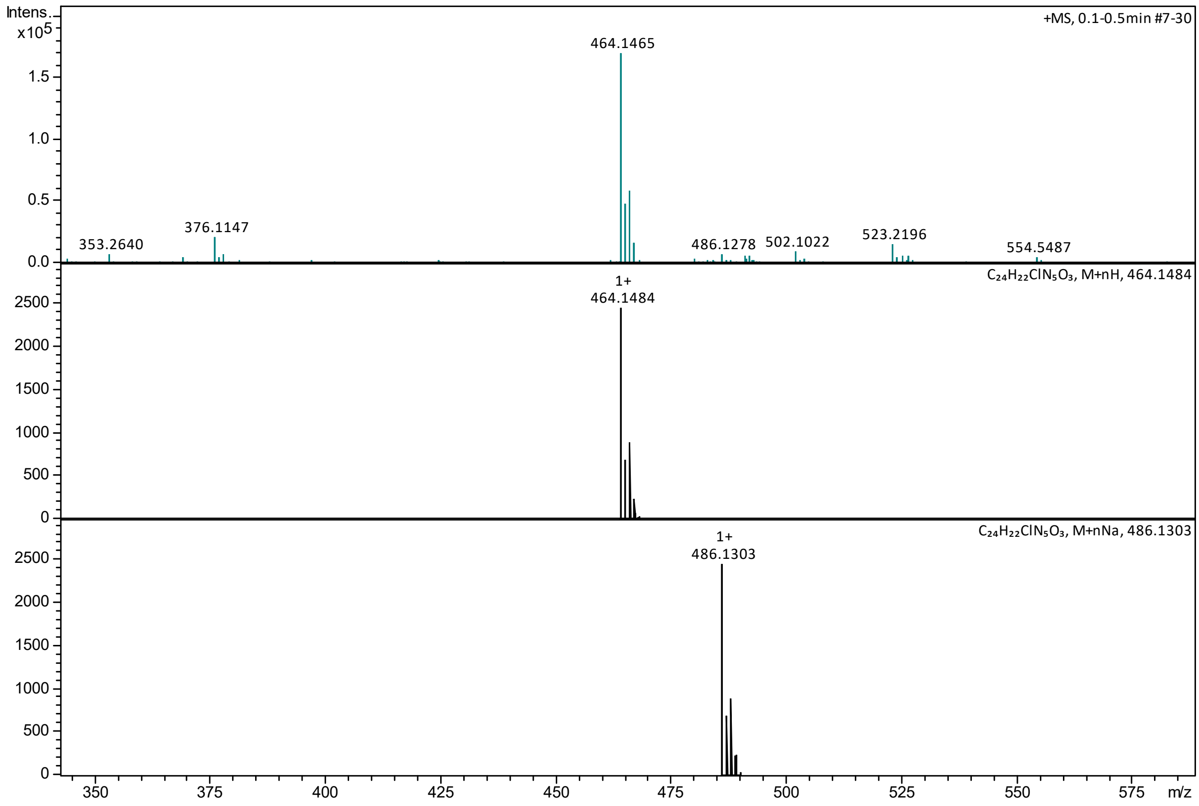
Intens.
x10^5
464.1465
376.1147
353.2640
486.1278
502.1022
523.2196
554.5487
+MS, 0.1-0.5min #7-30
C24H22ClN5O3, M+nH, 464.1484
1+
464.1484
C24H22ClN5O3, M+nNa, 486.1303
1+
486.1303
m/z

5c

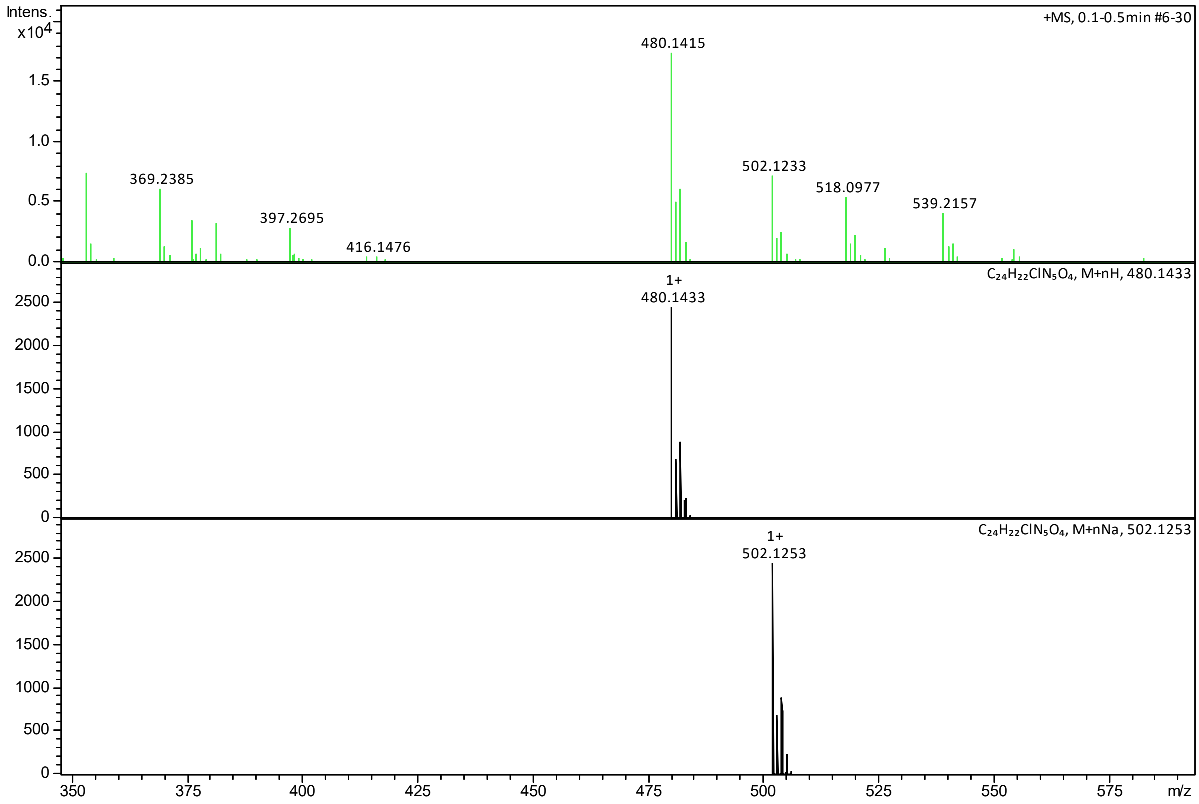
Intens.
x10^4
480.1415
369.2385
397.2695
416.1476
502.1233
518.0977
539.2157
+MS, 0.1-0.5min #6-30
C24H22ClN5O4, M+nH, 480.1433
1+
480.1433
C24H22ClN5O4, M+nNa, 502.1253
1+
502.1253
m/z

6c

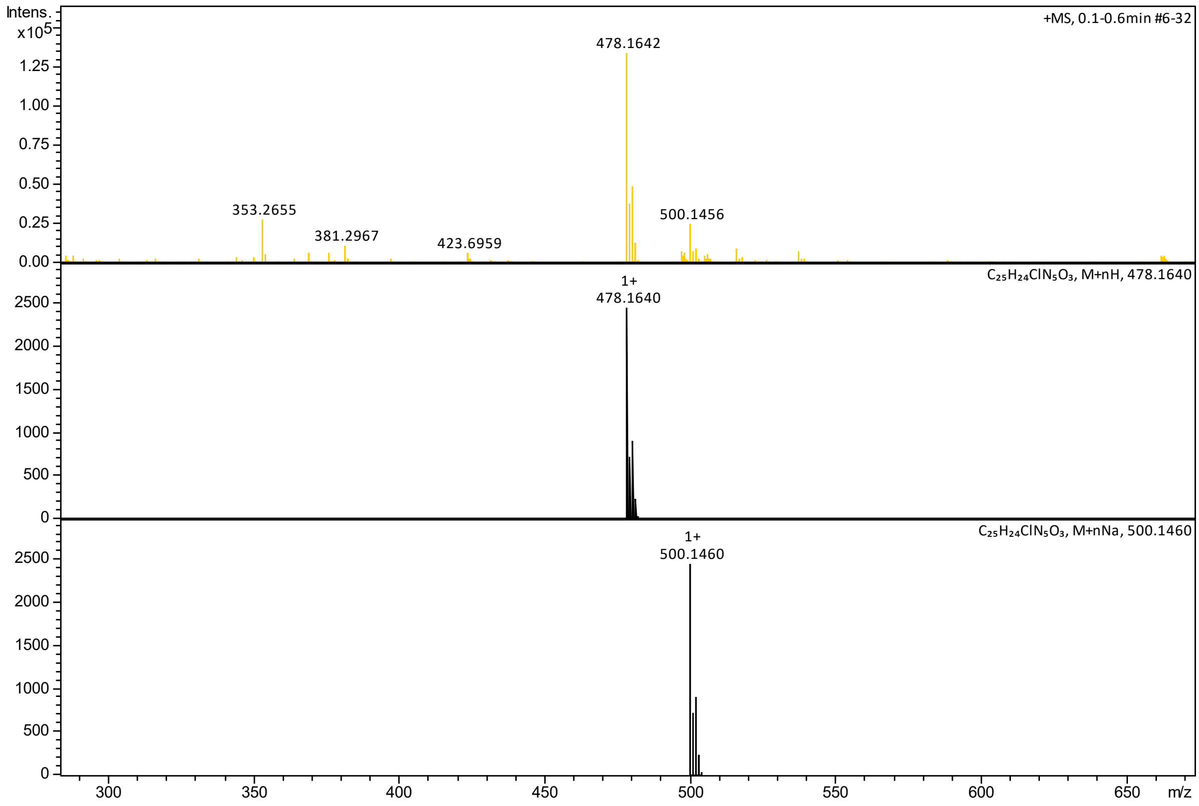

7c

Table S4 – FT-IR spectra of new compounds

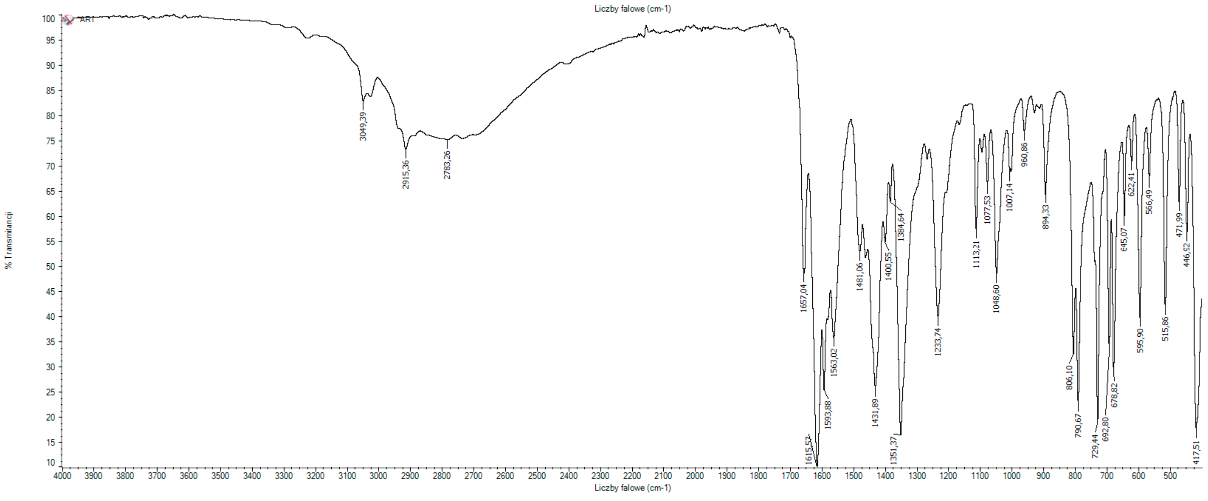

2c

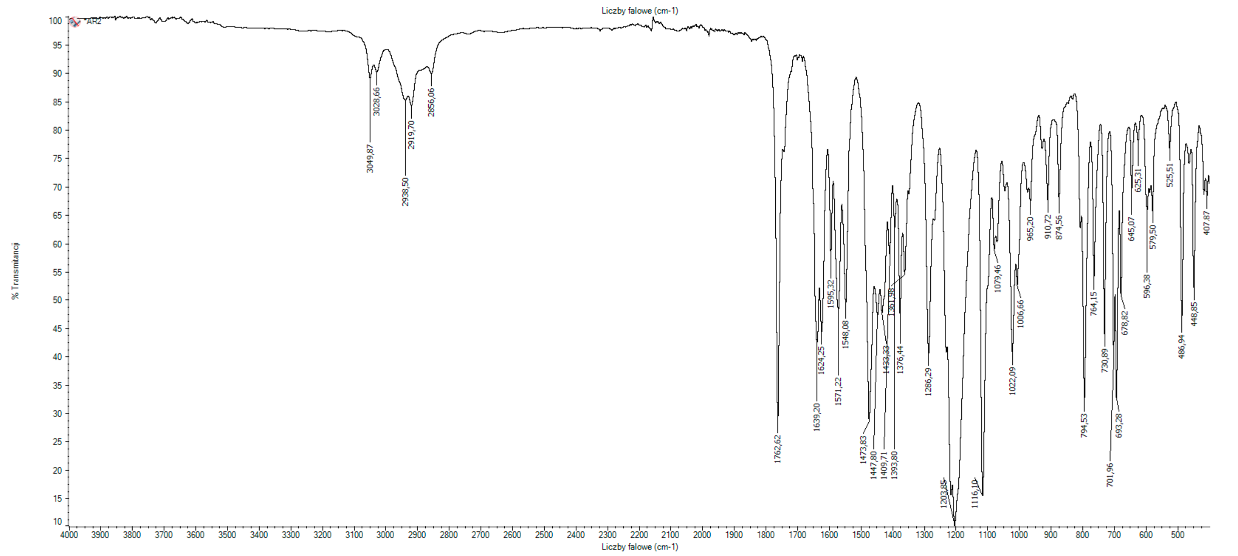
Liczby falowe (cm-1)
AR2
% Transmitancji
3049,87
3028,66
2938,50
2919,70
2856,06
1762,62
1639,20
1624,25
1595,32
1571,22
1548,08
1473,83
1447,80
1432,23
1409,71
1393,80
1381,88
1376,44
1286,29
1203,85
1116,19
1079,46
1022,09
1006,66
965,20
910,72
874,56
794,53
764,15
730,89
701,96
693,28
678,82
645,07
625,31
596,38
579,50
525,51
486,94
448,85
407,87
Liczby falowe (cm-1)

3c

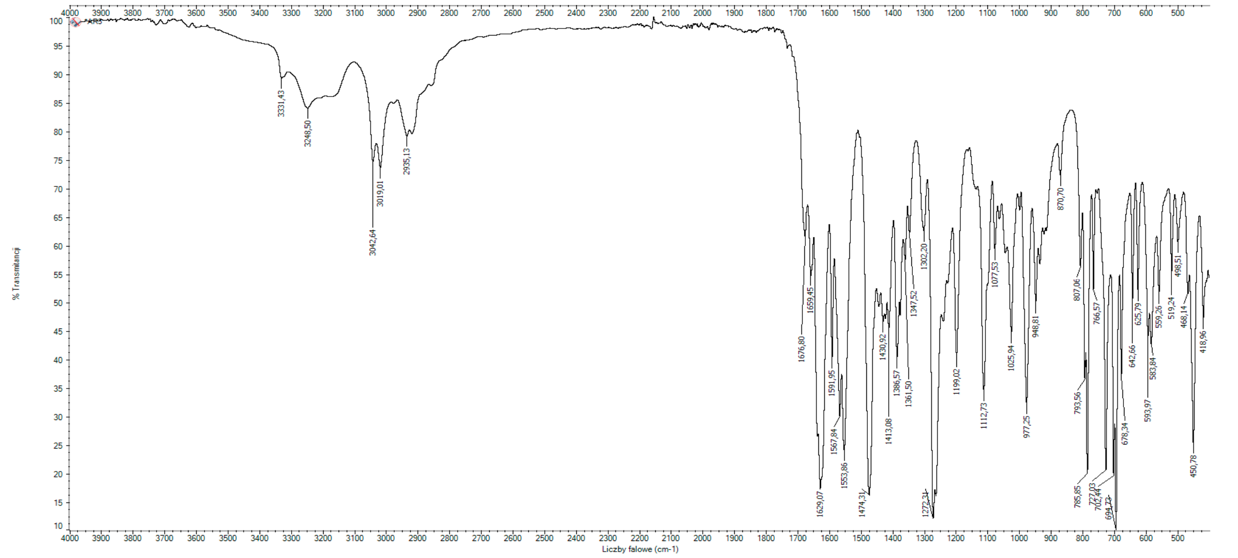
AR3
% Transmitancji
3331,43
3248,50
3042,64
3019,01
2935,13
1676,80
1659,45
1629,07
1591,95
1567,84
1553,86
1474,31
1430,92
1413,08
1386,57
1361,50
1347,52
1302,20
1277,31
1199,02
1112,73
1077,53
1025,94
977,25
948,81
870,70
807,06
793,56
785,85
766,57
722,03
702,44
694,73
678,24
642,66
625,79
593,97
583,84
559,26
519,24
498,51
468,14
450,78
418,96
Liczby falowe (cm-1)

4c

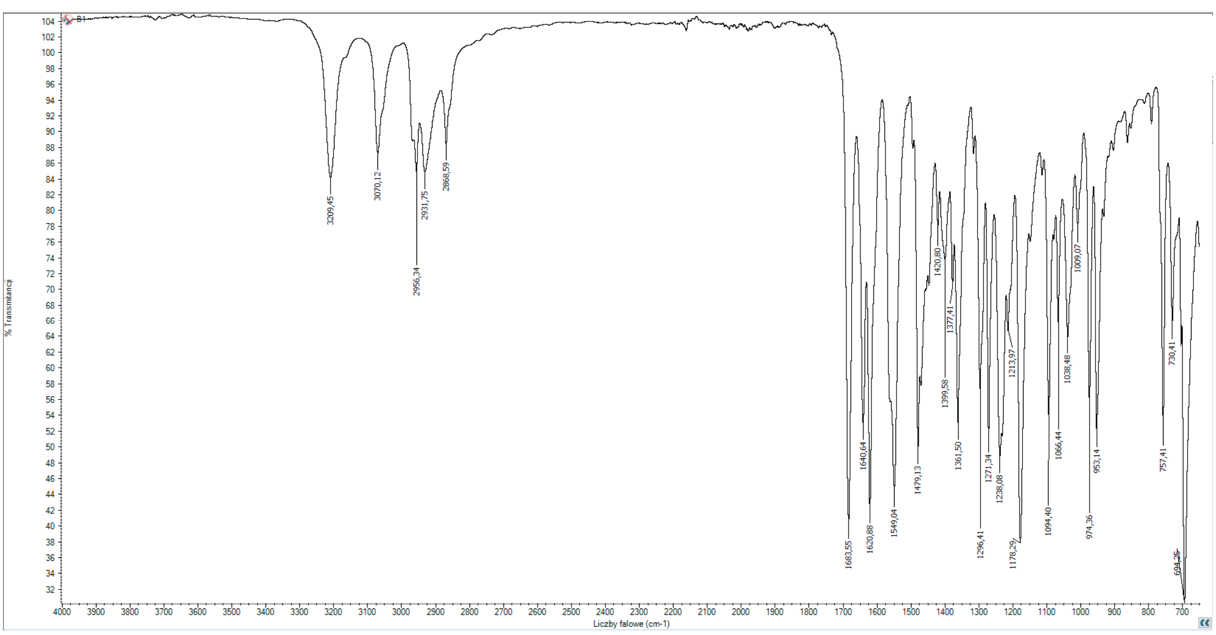
B1
% Transmitancji
3209,45
3070,12
2956,34
2931,75
2868,59
1683,55
1640,64
1620,88
1549,04
1479,13
1420,80
1399,58
1377,41
1361,50
1296,41
1271,34
1238,08
1213,97
1178,29
1094,40
1066,44
1038,48
1009,07
974,36
953,14
757,41
730,41
694,35
Liczby falowe (cm-1)

5a

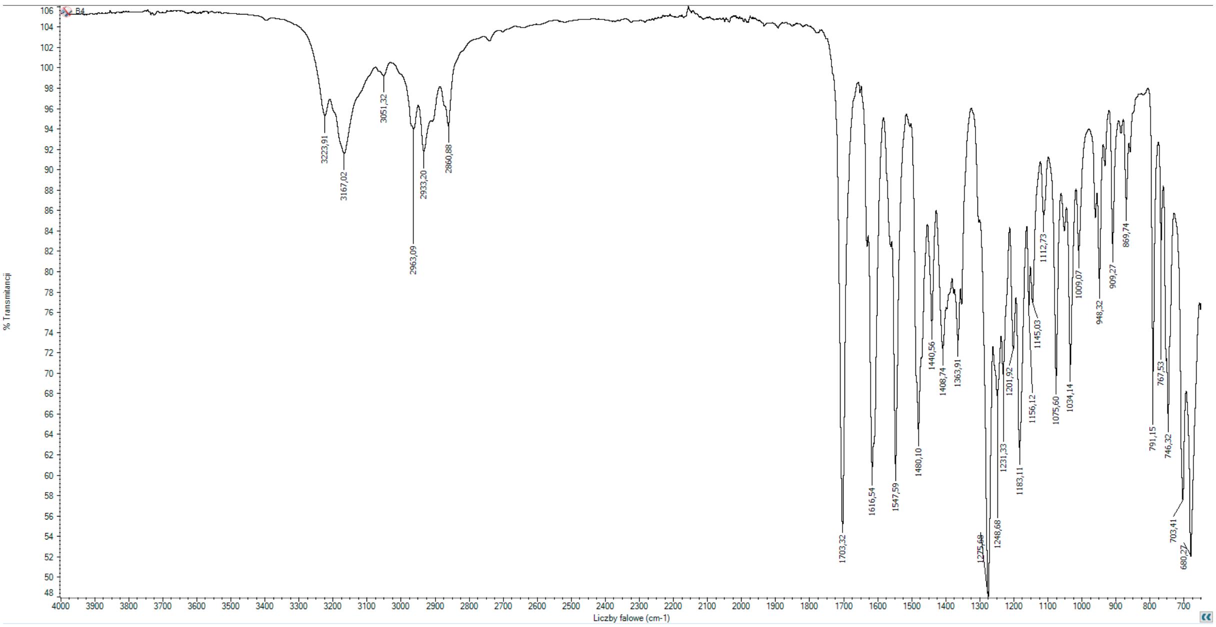

B4
% Transmitancji
Liczby falowe (cm-1)
3223,91
3167,02
3051,32
2963,09
2933,20
2860,88
1703,32
1616,54
1547,59
1480,10
1440,56
1408,74
1363,91
1275,69
1248,68
1231,33
1201,92
1183,11
1156,12
1145,03
1112,73
1075,60
1034,14
1009,07
948,32
909,27
869,74
791,15
767,53
746,32
703,41
680,27

6a

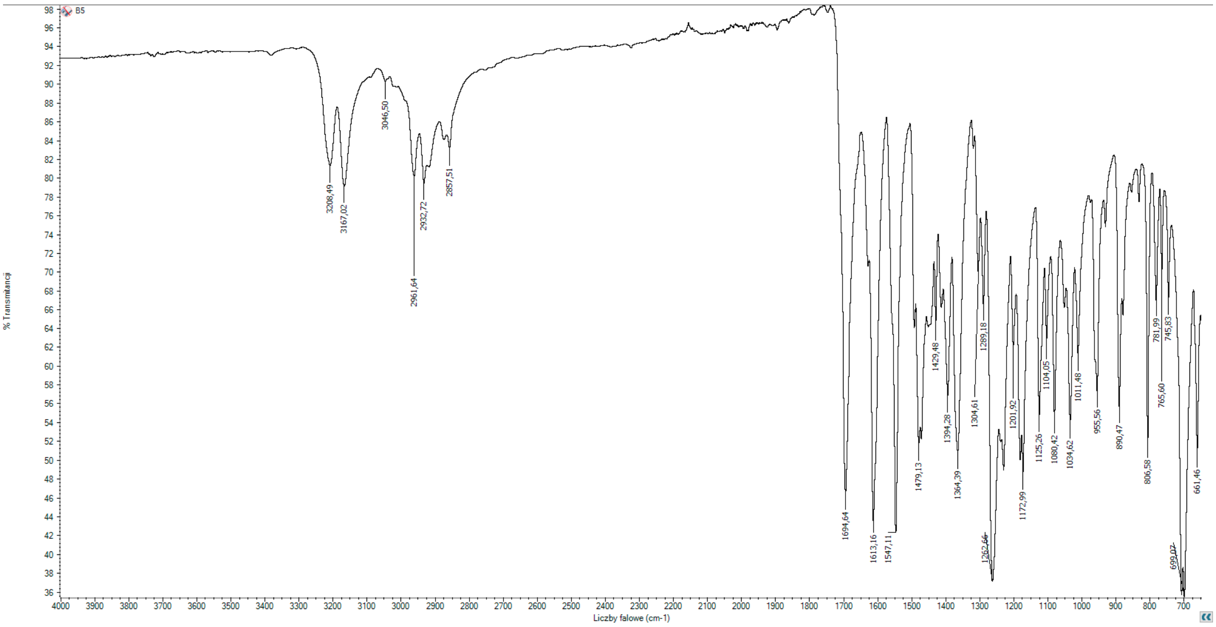

B5
% Transmitancji
Liczby falowe (cm-1)
3208,49
3167,02
3046,50
2961,64
2932,72
2857,51
1694,64
1613,16
1547,11
1479,13
1429,48
1394,28
1364,39
1304,61
1289,18
1262,66
1201,92
1172,99
1125,26
1104,05
1080,42
1034,62
1011,48
955,56
890,47
806,58
781,99
765,60
745,83
699,07
661,46

7a

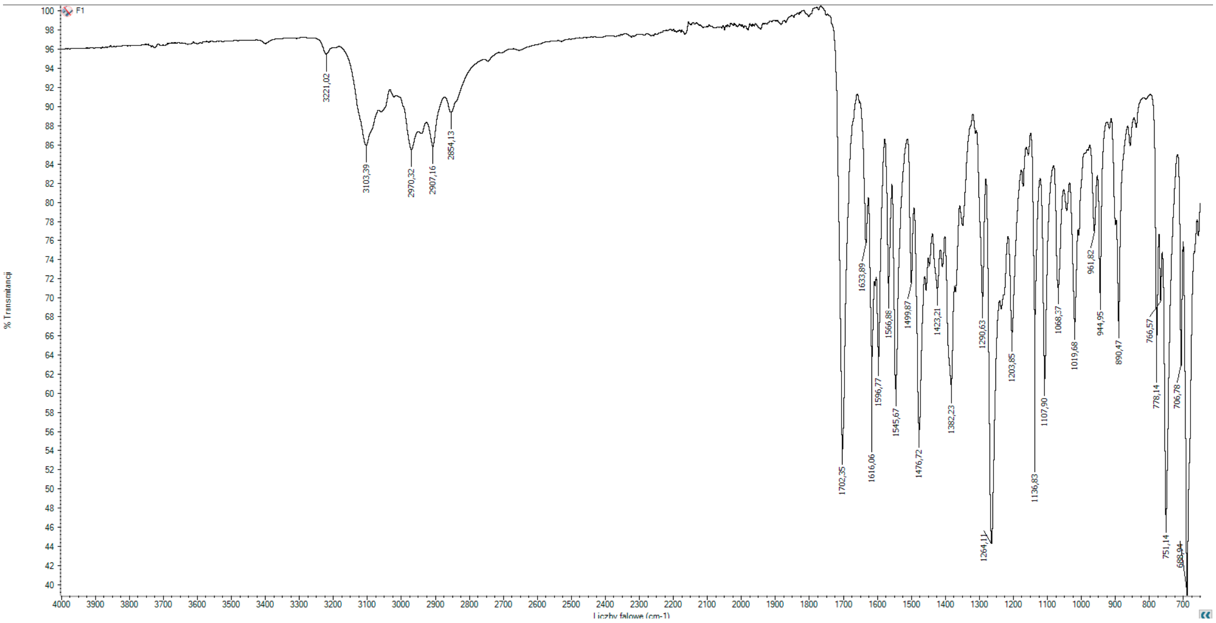
F1
% Transmitancja
Liczby falowe (cm-1)
3221,02
3103,39
2970,32
2907,16
2854,13
1702,35
1633,89
1616,06
1596,77
1566,88
1545,67
1499,87
1476,72
1423,21
1382,23
1290,63
1264,11
1203,85
1136,83
1107,90
1068,37
1019,68
961,82
944,95
890,47
778,14
766,57
751,14
706,78
688,94

5b

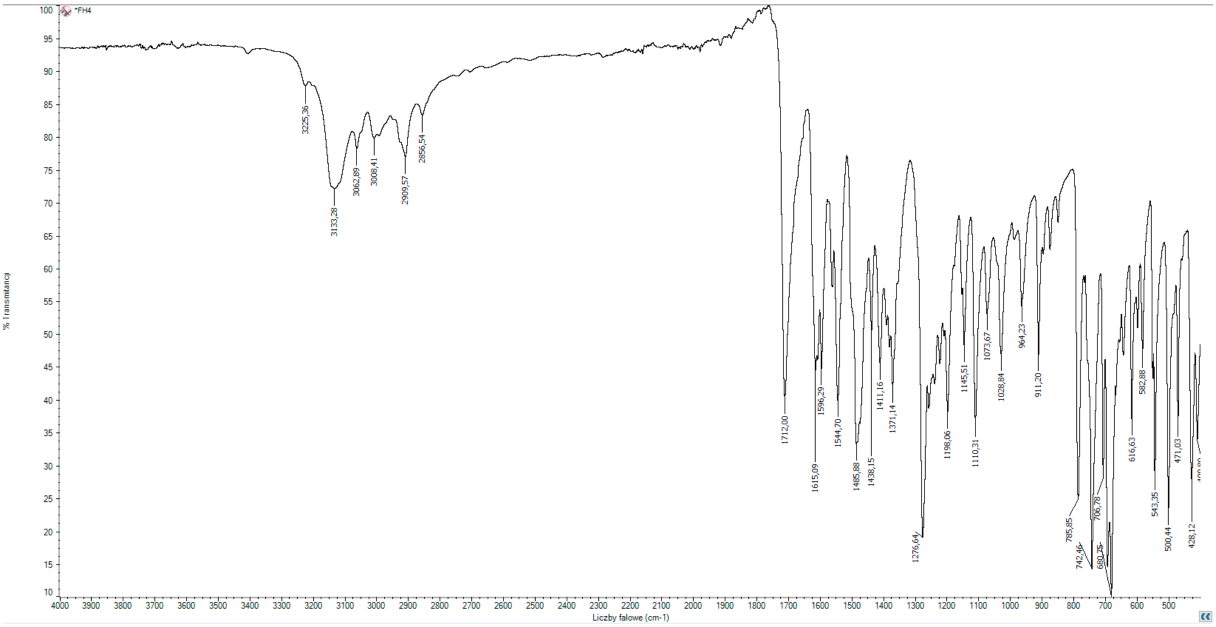
*FH4
% Transmitancja
Liczby falowe (cm-1)
3225,36
3133,28
3062,89
3008,41
2909,57
2856,54
1712,00
1615,09
1596,29
1544,70
1485,88
1438,15
1411,16
1371,14
1276,64
1198,06
1145,51
1110,31
1073,67
1028,84
964,23
911,20
785,85
742,46
706,78
680,75
616,63
582,88
543,35
500,44
471,03
428,12

6b

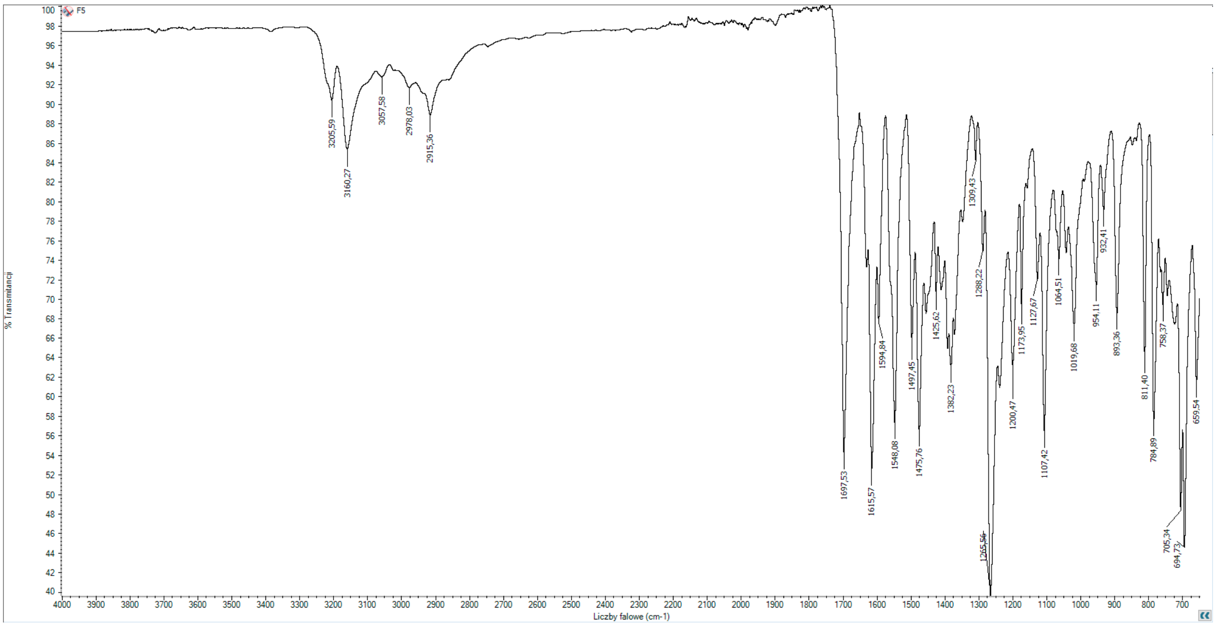
F5
% Transmitancji
Liczby falowe (cm-1)
3205,59
3160,27
3057,58
2978,03
2915,36
1697,53
1615,57
1594,84
1548,08
1497,45
1475,76
1425,62
1382,23
1309,43
1288,22
1265,56
1200,47
1173,95
1127,67
1107,42
1064,51
1019,68
954,11
932,41
893,36
811,40
784,89
758,37
705,34
694,73
659,54

7b

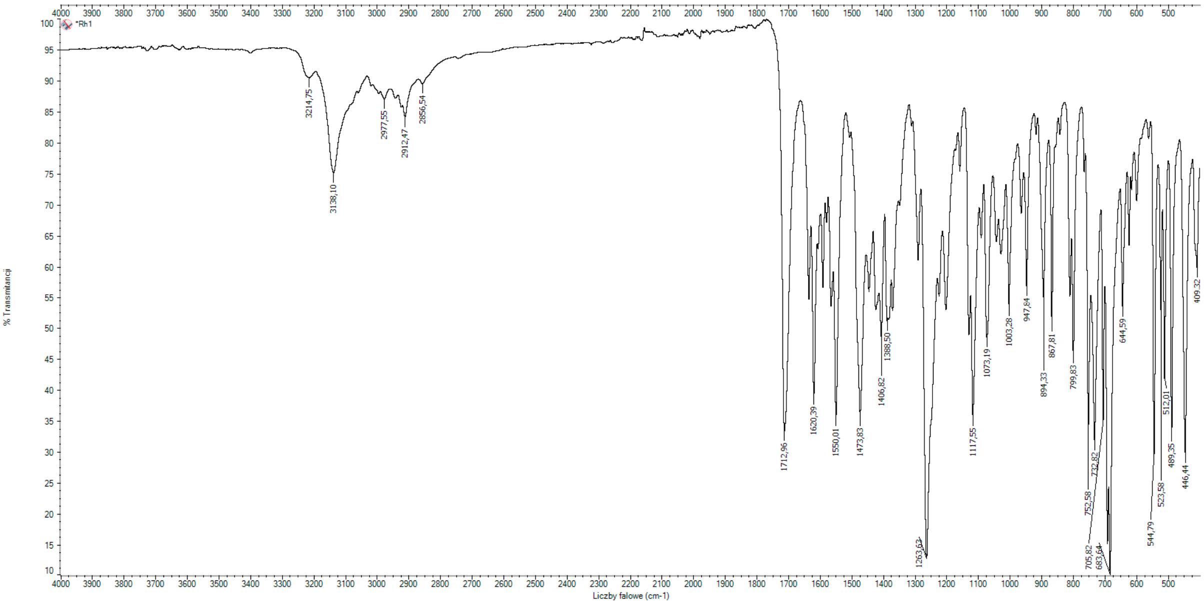
*Rh1
% Transmitancji
Liczby falowe (cm-1)
3214,75
3138,10
2977,55
2912,47
2856,54
1712,96
1620,39
1550,01
1473,83
1406,82
1388,50
1263,63
1117,55
1073,19
1003,28
947,84
894,33
867,81
799,83
752,58
732,82
705,82
683,64
644,59
544,79
523,58
512,01
489,35
446,44
409,32

5c

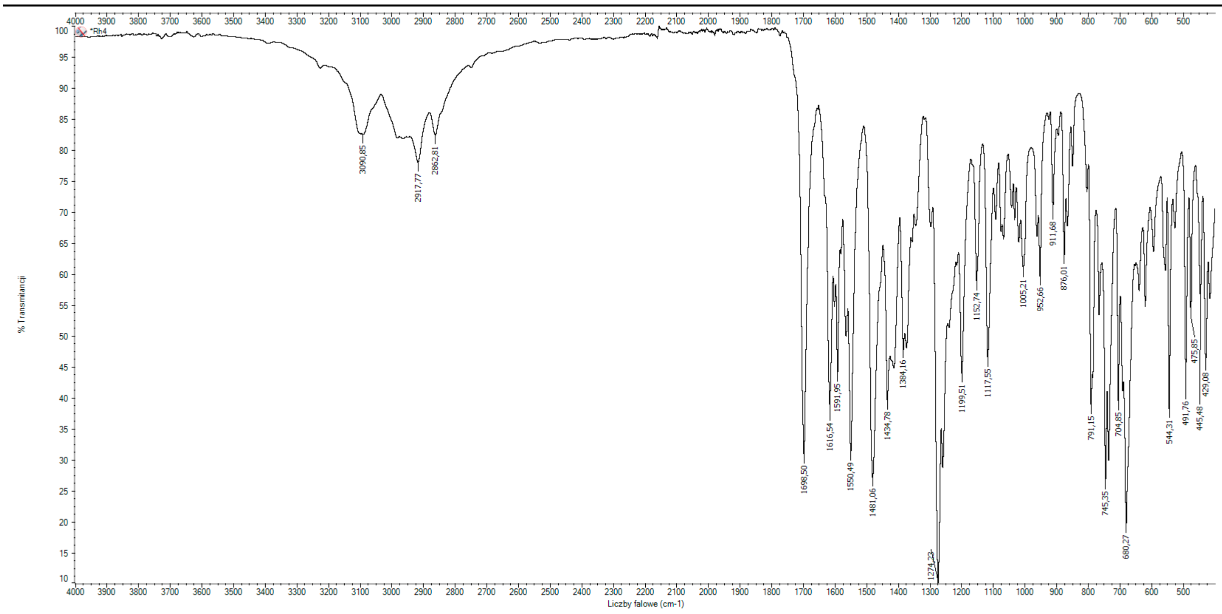

6c

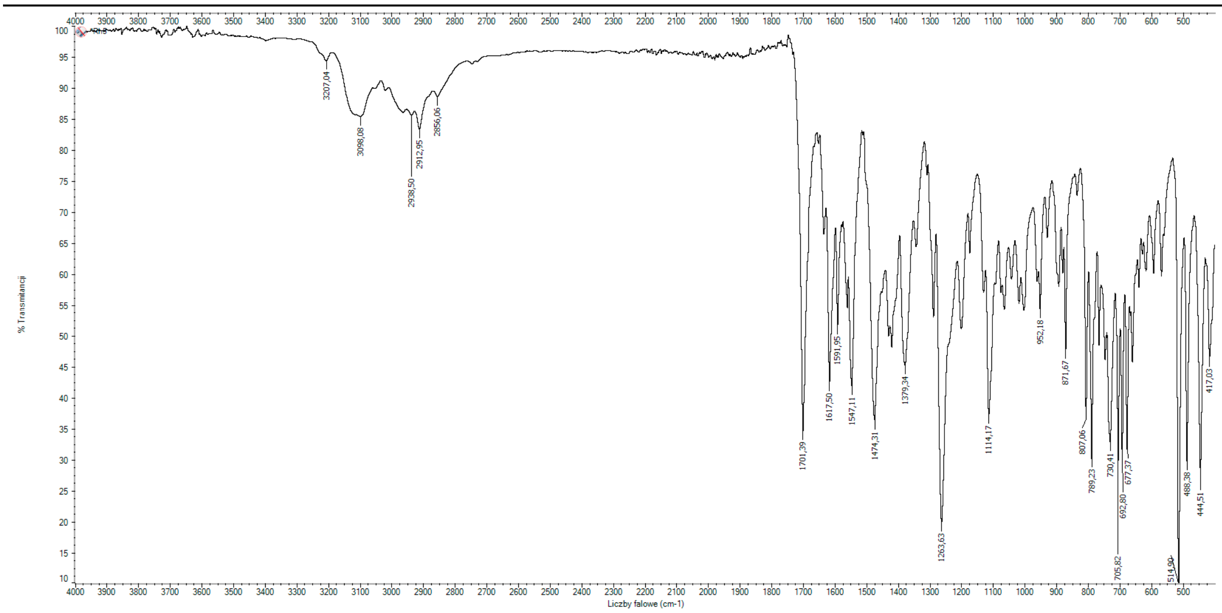

7c

Table S5 – Molecular formula strings (CSV) of reported compounds

	SMILE
**2c**	CN1NC(=O)c2c(C)n(c(C)c2C1=O)c3cccc(Cl)c3
**3c**	COC(=O)COC1=NN(C)C(=O)c2c(C)n(c(C)c12)c3cccc(Cl)c3
**4c**	CN1N=C(OCC(=O)NN)c2c(C)n(c(C)c2C1=O)c3cccc(Cl)c3
**5a**	CCCCn1c(C)c2C(=O)N(C)N=C(OCC(=O)N\N=C\c3ccccc3)c2c1C
**6a**	CCCCn1c(C)c2C(=O)N(C)N=C(OCC(=O)N\N=C\c3ccccc3O)c2c1C
**7a**	CCCCn1c(C)c2C(=O)N(C)N=C(OCC(=O)N\N=C\c3ccc(C)cc3)c2c1C

**5b**	CN1N=C(OCC(=O)N\N=C\c2ccccc2)c3c(C)n(c(C)c3C1=O)c4ccccc4
**6b**	CN1N=C(OCC(=O)N\N=C\c2ccccc2O)c3c(C)n(c(C)c3C1=O)c4ccccc4
**7b**	CN1N=C(OCC(=O)N\N=C\c2ccc(C)cc2)c3c(C)n(c(C)c3C1=O)c4ccccc4
**5c**	CN1N=C(OCC(=O)N\N=C\c2ccccc2)c3c(C)n(c(C)c3C1=O)c4cccc(Cl)c4
**6c**	CN1N=C(OCC(=O)N\N=C\c2ccccc2O)c3c(C)n(c(C)c3C1=O)c4cccc(Cl)c4
**7c**	CN1N=C(OCC(=O)N\N=C\c2ccc(C)cc2)c3c(C)n(c(C)c3C1=O)c4cccc(Cl)c4

Figure S1 - Flowchart summarizing the Materials and Methods section

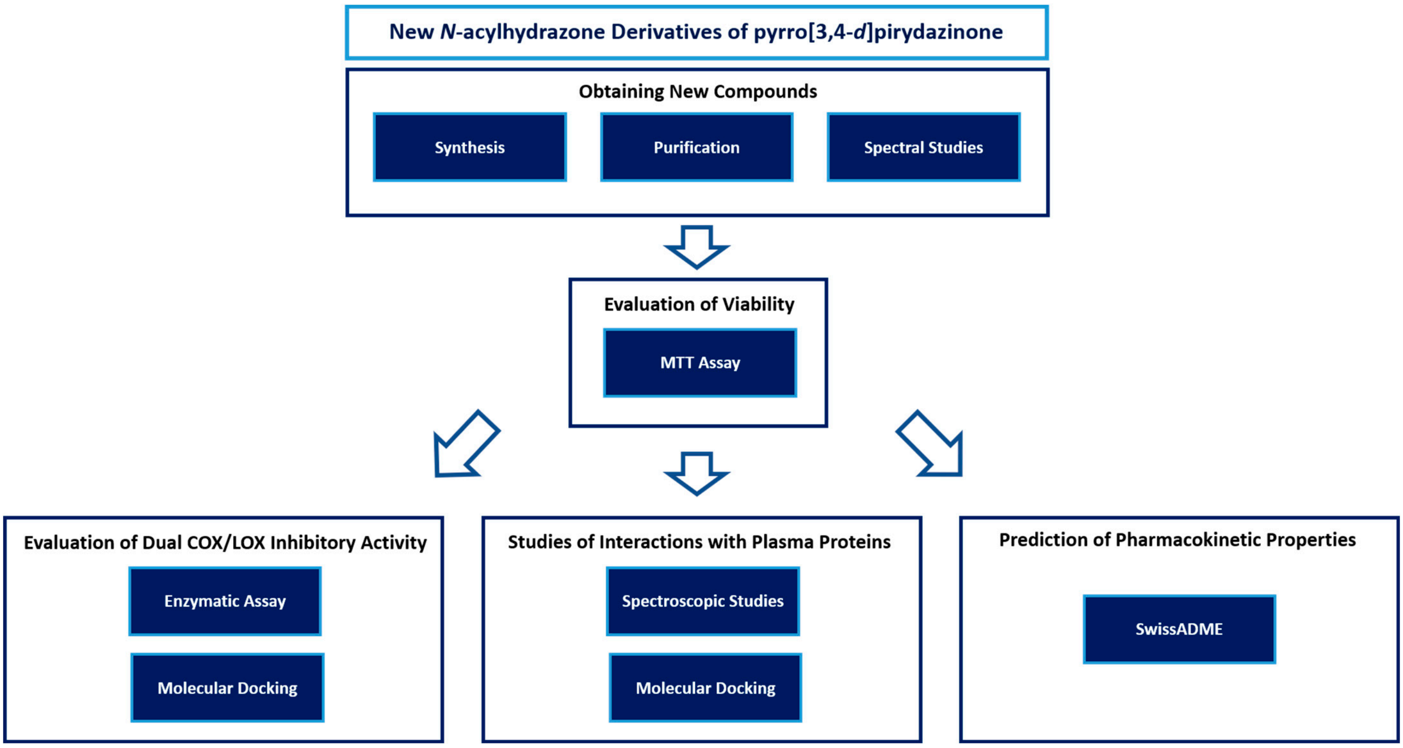

Supplement: Supplementary file 1 [file molecules-28-05479-s001.zip › molecules-2482564-supplementary.pdf]
